# Reducing CETP activity prevents memory decline in an Alzheimer’s disease mouse model

**DOI:** 10.1038/s44321-026-00482-w

**Published:** 2026-08-03

**Authors:** Jasmine Phénix, Isabel Sarty, Megan S Katz, Antonio Vázquez Cobá, Aarón Ernesto Marure Rojano, Hannah Nie, Anja Kerksiek, Indie Jerry, Clara S Berger, Robert S Kiss, Dieter Lütjohann, William A Pastor, Judes Poirier, Lisa Marie Munter, The PREVENT-AD Research Group, Isabel Sarty, Judes Poirier, Lisa Marie Munter

**Affiliations:** 1 Department of Pharmacology and Therapeutics, Faculty of Medicine and Health Sciences, McGill University, Montreal, QC Canada; 2 Cell Information Systems group, Bellini Life Sciences Complex, Montreal, QC Canada; 3 Centre de Recherche en Biologie Structurale (CRBS), 3649 Promenade Sir William Osler, Montreal, QC Canada; 4 Integrated Program in Neuroscience, McGill University, Montreal, QC Canada; 5 Centre for Studies on the Prevention of Alzheimer’s Disease, Verdun, QC Canada; 6 Molecular Neurobiology Unit, Douglas Mental Health University Institute, Research Centre, Verdun, QC Canada; 7 Department of Psychiatry, Faculty of Medicine and Health Sciences, McGill University, Montreal, QC Canada; 8 Department of Biochemistry, Faculty of Medicine and Health Sciences, McGill University, Montreal, QC Canada; 9 The Rosalind & Morris Goodman Cancer Institute, McGill University, Montreal, QC Canada; 10 Institute of Clinical Chemistry and Clinical Pharmacology, University Hospital Bonn, Bonn, Germany; 11 Division of Cardiology, Department of Medicine, Faculty of Medicine and Health Sciences, Research Institute of the McGill University Health Centre, McGill University, Montreal, QC Canada

**Keywords:** Neuroscience

## Abstract

Epidemiological studies have shown that lower activity of the cholesteryl ester transfer protein (CETP) correlates with reduced Alzheimer’s disease (AD) risk. While small-molecule CETP inhibitors like evacetrapib have previously been assessed for cardiovascular diseases, their involvement in AD has not been investigated. Here, we establish CETP as a novel pharmacological target for AD treatment. Using CETP transgenic mice crossed to a mouse model of amyloidosis and administering evacetrapib, we provide evidence that CETP inhibition maintained memory independent of classic AD markers, likely through maintained vascular health, while increasing hippocampal cholesterol and altering plasma lipoproteins. Using proteomic data of cerebrospinal fluid (CSF) from cognitively unimpaired individuals at risk for AD in the PResymptomatic EValuation of Experimental or Novel Treatments for AD (PREVENT-AD) cohort, we confirm that our mouse model reflects physiological changes in pre-symptomatic human subjects. We propose the repurposing of CETP inhibitors as an effective therapeutic strategy to delay or prevent cognitive impairment in AD.

## Synopsis

Drug repurposing offers a rapid strategy to identify small molecules that delay or prevent Alzheimer’s disease. In this study, inhibition of the cholesteryl ester transfer protein (CETP) emerges as a promising preventative therapy.

- Pharmacological CETP inhibition preserved cognitive performance in a mouse model of amyloidosis (APP-expressing mice).
- Mechanistically, CETP inhibition benefits cognition, at least in part, by enhancing brain endothelial health.
- Findings from the PREVENT-AD cohort in humans are consistent with the observations made in the mouse model.

## Abstract (web summary)

Drug repurposing offers a rapid strategy to identify small molecules that delay or prevent Alzheimer’s disease. In this study, inhibition of the cholesteryl ester transfer protein (CETP) emerges as a promising preventative therapy.

## Graphical abstract

**Figure:**
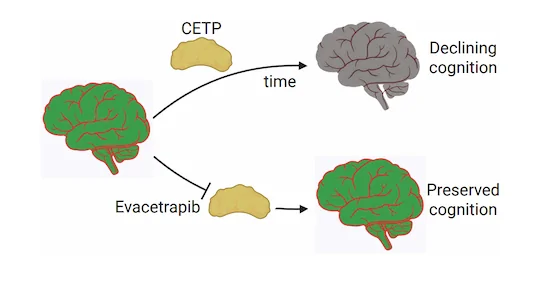

**Figure:**
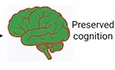

The paper explained **Problem**

Alzheimer’s disease is the most prevalent neurodegenerative disorder affecting about 35 million people worldwide, a number projected to rise with population aging. It is characterized by progressive memory loss and cognitive decline, ultimately leading to loss of independence. Despite its high burden, safe and effective preventive therapies remain unavailable. Alzheimer’s disease also imposes a substantial economic burden, with global costs estimated at close to one trillion USD annually.

Drug repurposing represents a rapid and cost-effective strategy to identify preventive treatments. Genetic studies have shown that low-activity variants of the cholesteryl ester transfer protein (CETP) are associated with protection against Alzheimer’s disease, particularly in early disease stages and in carriers of the apolipoprotein E4 (APOE4), the strongest genetic risk factor for the disease. Notably, pharmacological CETP inhibitors have already been developed for cardiovascular indications; however, their potential impact on Alzheimer’s disease remains largely unexplored.

**Results**

To assess the effect of CETP inhibition on cognition in a mouse model of Alzheimer’s disease, we crossed CETP transgenic mice with amyloid precursor protein (APP) transgenic mice. Animals were treated with the CETP inhibitor evacetrapib from 3 to 5.5 months of age. CETP inhibition preserved cognitive performance in both CETP transgenic and CETP×APP double transgenic mice, indicating that the beneficial effects of CETP inhibition outweigh APP-associated impairments.

Mechanistically, CETP inhibition improved endothelial function and promoted a redistribution of cholesterol within the hippocampus. Importantly, analyses from the human PREVENT-AD cohort support the CETP-associated effects observed in our mouse model, highlighting the translational relevance of these findings.

**Impact**

Our preclinical findings suggest that pharmacological CETP inhibition may delay—or potentially halt—the progression of Alzheimer’s disease. Importantly, several CETP inhibitors have already been developed by multiple pharmaceutical companies and advanced to phase III clinical trials, where they were shown to be safe and well-tolerated in older populations, although none have yet received regulatory approval.

Notably, the BROADWAY trial, conducted in patients with familial hypercholesterolemia, included a substudy with measurements of plasma Alzheimer’s disease biomarkers and reported stabilization or reduction of these markers, providing preliminary human evidence consistent with our preclinical findings.

Together, these data support the concept that pharmacological CETP inhibition represents a promising, rapid, and cost-effective strategy for Alzheimer’s disease prevention. This approach may be particularly beneficial for APOE4 carriers, who account for over 60% of sporadic Alzheimer’s disease cases.

## Introduction

The cholesteryl ester transfer protein (CETP) is a plasma glycoprotein primarily secreted by the liver but may also be present in the central nervous system (CNS) in humans (Albers et al, 1992; Barter et al, 2003; Siletti et al, 2024). CETP facilitates the bidirectional exchange of neutral lipids—cholesteryl esters and triglycerides—between lipoproteins in plasma, including pro-atherogenic low-density lipoproteins (LDL) and anti-atherogenic high-density lipoproteins (HDL), ultimately increasing LDL-cholesterol levels (Pattnaik et al, 1978; Qiu et al, 2007). Consequently, reduced CETP activity protects against atherosclerosis, and cardiovascular events have been extensively investigated over the past decades (Ansell and Hobbs, 2006; Nicholls et al, 2022; Schmidt et al, 2021; Tall et al, 1997). This line of research led to the development of small-molecule CETP inhibitors (Bagdade et al, 2017; Group et al, 2017; Lincoff et al, 2017; Nicholls et al, 2015; Schwartz et al, 2012). Despite the safety and effectiveness of novel CETP inhibitors in raising HDL-cholesterol, clinical trials failed to demonstrate superior cardiovascular benefits when compared to statins, causing a halt in research on this class of compounds (Group et al, 2017; Lincoff et al, 2017; Nicholls et al, 2015; Schwartz et al, 2012). However, new evidence has highlighted the impact of CETP activity on several other diseases distinct from cardiovascular diseases, such as type 2 diabetes mellitus, cancer, and neurodegenerative diseases, including Parkinson’s disease, Lewy body dementia, and Alzheimer’s disease (AD) (Dangas et al, 2022; Esau et al, 2016; Schmidt et al, 2024). In addition, we and others have shown that CETP inhibitors enter the brain, demonstrating versatility in targeting both peripheral and brain tissue (Phenix et al, 2023; Takubo et al, 2014). As such, these findings have reignited interest in the therapeutic potential of CETP inhibitors.

Several lines of evidence support the hypothesis that reducing CETP activity will preserve memory in AD. Firstly, epidemiological studies comparing centenarians to the general population have revealed that *CETP* variants with reduced activity are linked to longevity, preserved cognitive function, and a lower risk of dementia (Barzilai et al, 2006; Barzilai et al, 2003; Sun et al, 2013). Intriguingly, low CETP activity overrides detrimental effects of the apolipoprotein E4 allele (*APOE ε4* describes the allele, APOE4 the protein) (Chen et al, 2008; Lythgoe et al, 2015; Murphy et al, 2012; Rodriguez et al, 2006; Sundermann et al, 2016), the greatest genetic risk factor for AD (Poirier et al, 1993; Strittmatter et al, 1993). A recent pre-specified clinical substudy of the BROADWAY trial showed that CETP inhibitor obicetrapib significantly slowed AD biomarker progression with the most pronounced impact on phosphorylated-tau (p-tau)217 in APOE4 carriers (Davidson et al, 2026). Given that approx. 65% of all AD patients are APOE4 carriers (Strittmatter et al, 1993), CETP inhibition holds great potential as a novel therapeutic target in AD research (Mehta et al, 2023). Secondly, CETP has a specific, non-promiscuous function and thus constitutes an excellent candidate for pharmacological inhibition (Barter et al, 2003; Nicholls et al, 2015; Tsutsumi et al, 2001). This notion is supported by rare cases of individuals with low or no *CETP* expression, who are overall healthy, consequently demonstrating that CETP is not essential for living long and healthy lives (Inazu et al, 1993; Nordestgaard et al, 2022; Yamashita et al, 1990). Lastly, epidemiological studies investigating high mid-life LDL and total cholesterol serum levels showed an association with a greater risk of dementia up to three decades before symptom onset (Solomon et al, 2007; Solomon et al, 2009). Considering that CETP raises LDL-cholesterol in plasma, its inhibition will reduce this established AD risk factor. Thus, repurposing of safe CETP inhibitors as a therapeutic option for AD holds significant promise, especially in individuals at risk who are still asymptomatic.

Studying the effects of CETP on AD pathology using mice presents a challenge, as mice do not naturally express CETP or homologous proteins (Agellon et al, 1991; Jiang et al, 1992; Tsutsumi et al, 2001). Consequently, their lipoprotein profile differs from that of humans. Mouse plasma predominantly contains HDL particles, with lower levels of LDL and very low-density lipoprotein (VLDL) particles, unlike humans, who have a higher proportion of LDL and VLDL (Steenbergen et al, 2010). To address this challenge, a mouse strain expressing the human *CETP* gene under its own human endogenous promoter was previously generated (CETPtg), resulting in a human-like lipid profile distinct from wild-type (WT) mice and has since been widely used in cardiovascular research (Jiang et al, 1992). In this study, we crossed an AD mouse model of amyloidosis, the McGill-APP-Thy1 (APPtg) mice (Ferretti et al, 2012), which display age-associated memory deficits, with CETPtg mice and administered the selective CETP inhibitor evacetrapib to investigate the role of CETP in AD as well as determine whether CETP inhibition preserves memory in double transgenic mice (APPtg/CETPtg). Our findings confirm that CETP inhibition preserves memory function in both CETPtg and APPtg/CETPtg mice, suppressing the negative effects of the amyloid precursor protein (APP). We attributed this maintenance to an altered plasma lipoprotein landscape, differences in brain lipid distribution, and maintained vascular health in the brain. Most importantly, results from our mouse model were replicated in the human PResymptomatic EValuation of Experimental or Novel Treatments for AD (PREVENT-AD) cohort, consisting of asymptomatic individuals at high risk of developing AD due to a parental history, establishing CETP as a powerful target in AD prevention.

## Results

### Evacetrapib successfully inhibits CETP in mice

**Figure 1:**
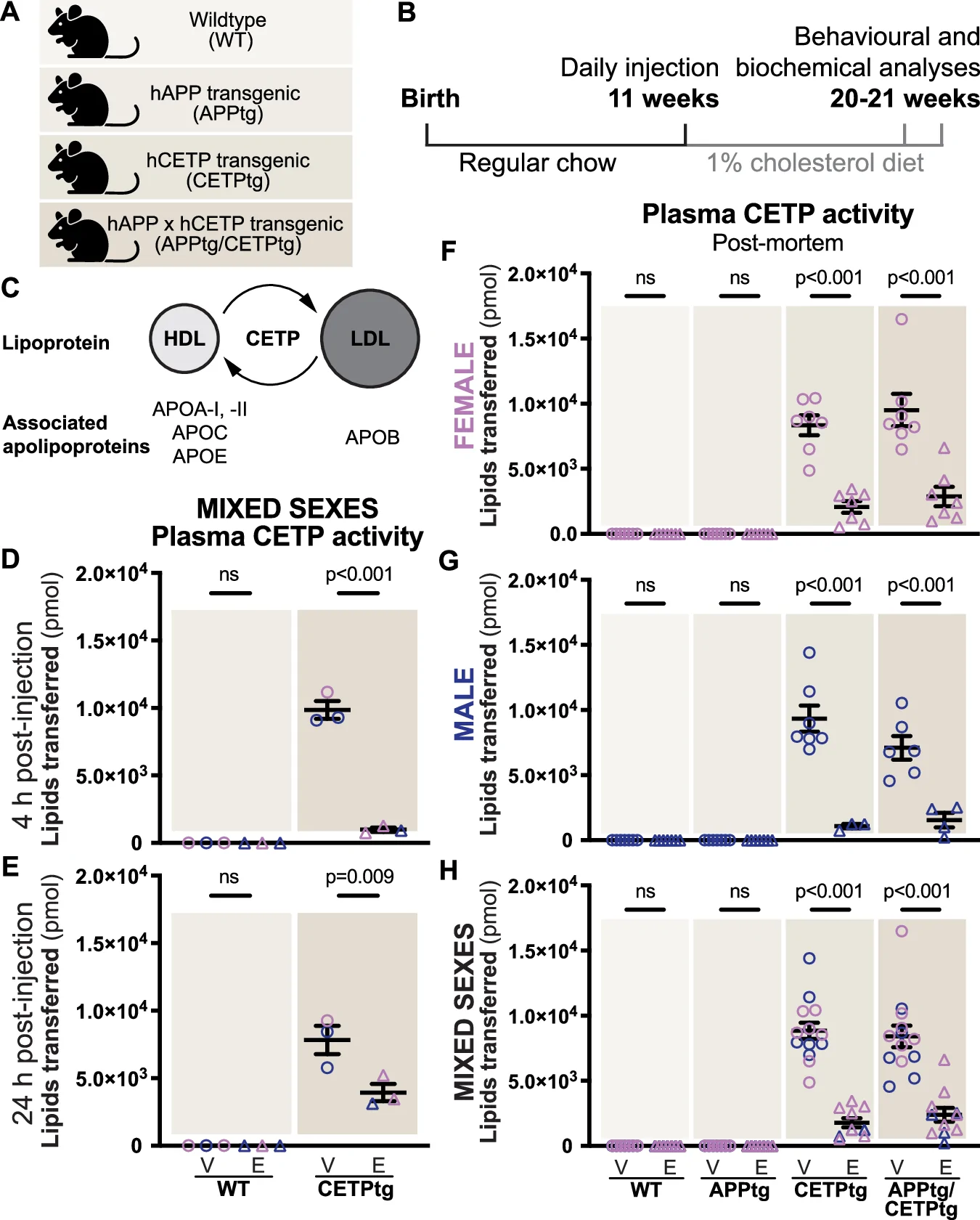
Evacetrapib treatment decreases CETP activity. (**A**) Scheme of the experimental design created with BioRender.com. (**B**) Timeline of the mouse study, including behavioral assays and organ collection. (**C**) Scheme of how plasma CETP transfers neutral lipids between ApoA-I-containing HDL and ApoB-containing LDL particles. (**D**, **E**) CETP activity quantification at 4 h or 24 h post-injection after two weeks of treatment in a subset of mice; two-way ANOVA followed by Tukey’s post hoc test, *n* = 3. Exact *P* value for CETPtg in (**D**) *P* < 0.001. (**F**, **G**) CETP activity measured at the time of sacrifice 4 h post-injection. Data are shown stratified by sex (*n* = 6–8). Statistical inference was based on a three-way ANOVA including sex (*P* = 0.12), genotype (*P* < 0.001), and treatment (*P* < 0.001), followed by Tukey’s post hoc test. Exact *P* values for (**F**) CETPtg *P* < 0.001; APPtg/CETPtg *P* < 0.001; for (**G**) CETPtg *P* < 0.001; APPtg/CETPtg *P* < 0.001. (**H**) Data from (**F**, **G**) shown combined for visualization only (*n* = 12–14) analyzed by a two-way ANOVA test and Tukey’s post hoc test. Exact *P* values CETPtg *P* < 0.001; APPtg/CETPtg *P* < 0.001. (**D**–**H**) Data is shown as a scatter plot, indicating mean ± SEM with each point depicting a sample from one mouse. Circles: vehicle (V); triangles: evacetrapib (E); female: violet; male: indigo. Source data are available online for this figure.

To explore the impact of CETP on AD pathophysiology in a preclinical model, APPtg mice and CETPtg mice were crossed to generate double transgenic APPtg/CETPtg. APPtg, CETPtg, and WT mice were used as littermate controls (Fig. 1A). Mice were fed a regular chow from birth until week 11, and then switched to a 1% cholesterol diet based on Oestereich et al to induce CETP expression via nuclear LXR receptor elements and increase plasma LDL levels in CETP-expressing mice (Luo and Tall, 2000; Oestereich et al, 2022). Mice received either vehicle or evacetrapib at 30 mg/kg/day intraperitoneally for 11 weeks (Fig. 1B). To assess drug-target engagement, we measured CETP activity in plasma during the trial and at endpoint (Fig. 1C–H). CETP activity was significantly reduced 4 h after evacetrapib treatment compared to the vehicle group (Fig. 1D). At 24 h, CETP activity rebound while still being reduced showing a transient inhibition of our dosage (Fig. 1E). As expected, no CETP activity was detected in WT mice. Similarly, terminal plasma collection 4 h after evacetrapib administration showed that CETPtg and APPtg/CETPtg mice exhibited significantly lower CETP activity than their vehicle-treated counterparts (Fig. 1F–H), confirming effective drug-target engagement in our mouse model.

### Evacetrapib remodels the lipoprotein profile in mice

**Figure 2:**
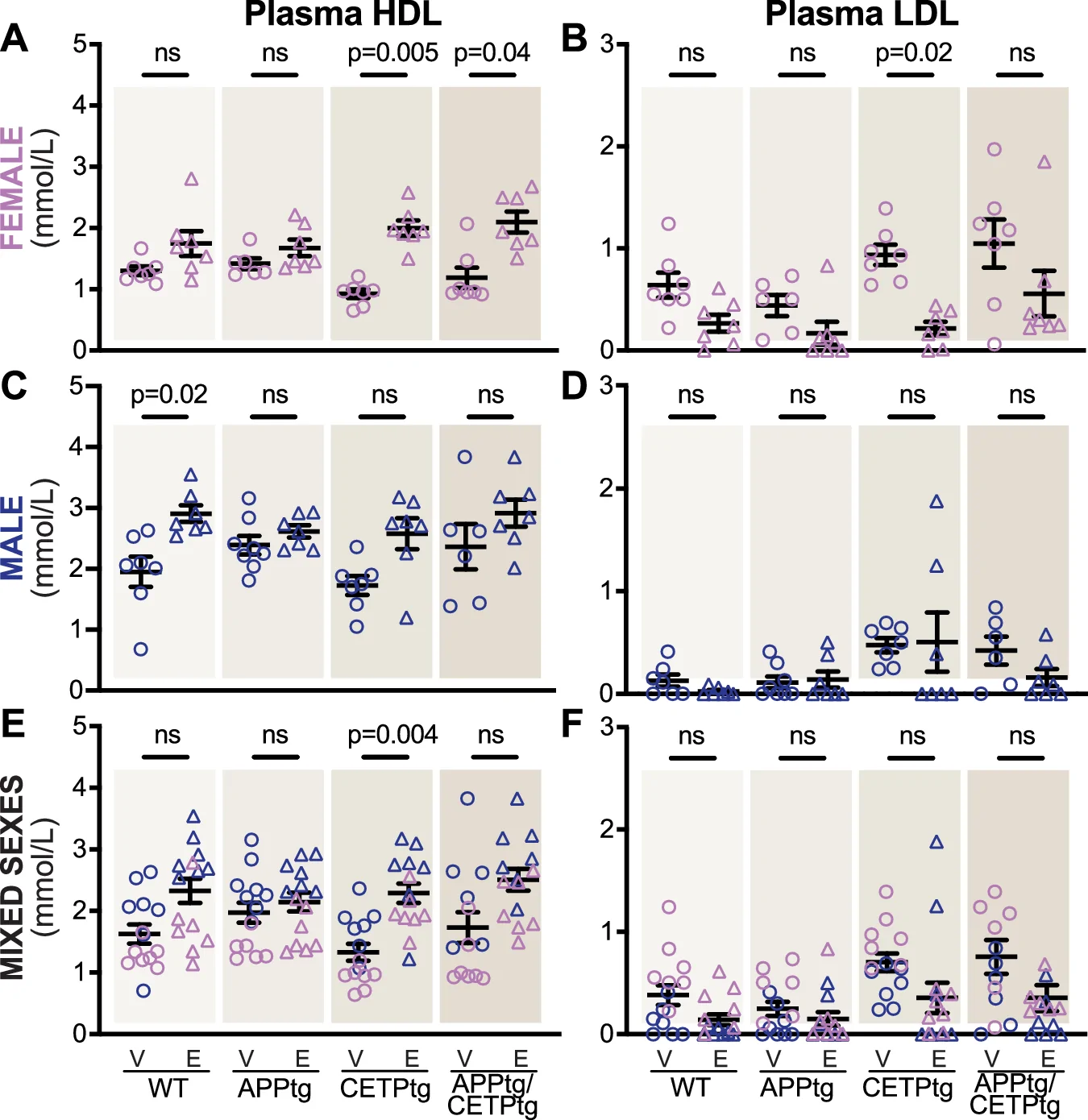
Evacetrapib treatment changes the lipoprotein profile in CETP-expressing mice. (**A**–**D**) Plasma lipoproteins HDL and LDL were quantified. Data are shown stratified by sex (*n* = 6–8). Statistical inference was based on a three-way ANOVA including sex, genotype, and treatment, followed by Tukey’s post hoc test (genotype HDL *P* = 0.05, LDL *P* < 0.001; sex HDL/LDL *P* < 0.001; treatment HDL/LDL *P* < 0.001). (**E**, **F**) Data from (**A**, **C**) or (**B**, **D**) shown and with sexes combined for visualization only (*n* = 12–14)*;* two-way ANOVA followed by Tukey’s post hoc test. Scatter dot plots with indicated mean ± SEM with each point depicting a sample from one mouse. Circles: vehicle (V); triangles: evacetrapib (E); female: violet; male: indigo. Source data are available online for this figure.

As a result of CETP activity and inhibition, the plasma lipoprotein profile is expected to change. As in previous reports (Agellon et al, 1991; Jiang et al, 1992), CETP activity decreased HDL and increased LDL levels by trend in vehicle-treated CETP-expressing mice as compared to WT or APPtg mice (Fig. 2A–F). Using a three-way ANOVA test on the combined data, we found that sex is a major driver of HDL and LDL differences, in addition to genotype and treatment (Fig. 2A–D). Accordingly, CETP inhibition in female CETPtg mice showed statistically significant changes in HDL and LDL levels as compared to their respective vehicle controls (Fig. 2A,B). A correlation analysis showed a positive correlation between CETP activity and LDL, and a negative correlation with HDL, except for HDL in males (Fig. S1A–F). Total plasma cholesterol, triglyceride, and VLDL levels varied significantly between sexes. However, they remained consistent across treatments and genotypes (Fig. S1G–O).

### Evacetrapib maintains memory in CETPtg and APPtg/CETPtg mice

**Figure 3:**
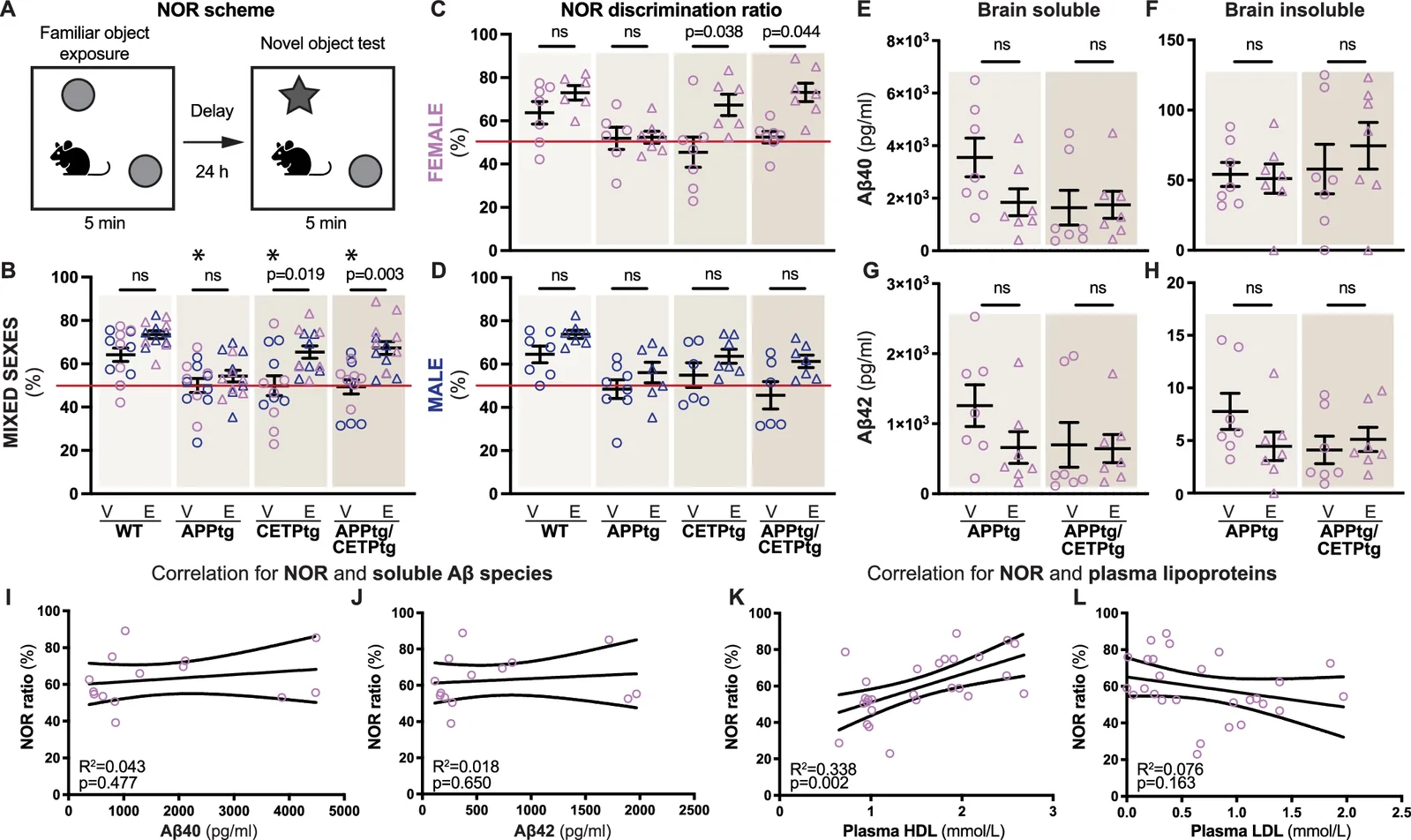
Memory in CETP-expressing mice is preserved with evacetrapib. (**A**) Scheme of the NOR test created with BioRender.com. (**B**) NOR scores combined for both sexes (*n* = 12–14). Red line indicates the 50% discrimination ratio at which mice cannot differentiate between the familiar and the novel object, suggesting memory impairment. Two-way ANOVA followed by Tukey’s post hoc test. *depict significant differences to the WT vehicle group (for APPtg-V *P* = 0.03; CETPtg-V *P* = 0.035; APPtg/CETPtg-V *P* = 0.025). (**C**, **D**) NOR scores for each sex for visualization. Two-way ANOVA followed by Tukey’s post hoc test. (**E**–**H**) Soluble and insoluble Aβ40 and Aβ42 peptides were quantified in brain homogenates of female mice by ELISA. (**B**–**H**) Scatter dot plots with indicated mean ± SEM with each point as a biological replicate. (**I**–**L**) Linear correlations of Aβ40 and Aβ42 versus cognition (NOR), *n* = 14, (**I**, **J**) as well as HDL and LDL versus cognition (NOR), *n* = 27–28 (**K**, **L**) in female APPtg and APPtg/CETPtg mice on evacetrapib or vehicle. Data are shown as scatter plots with a simple linear regression line. Pearson’s correlation assuming normality, and the 95% confidence interval. R-squared (*R*^2^) and *P* values (*P*) are indicated. Source data are available online for this figure.

To reveal whether CETP activity affects cognitive performance in our new mouse model similarly to previously described human epidemiological studies, we assessed recognition memory using the novel object recognition (NOR) test. Mice were assessed at 21 weeks of age, which is the earliest stage at which cognitive impairment becomes detectable in APPtg mice (Fig. 3A–D). WT mice showed no impairment, regardless of sex or treatment, demonstrating that evacetrapib had no negative impact on memory. APPtg mice displayed expected memory impairment, independent of treatment, showing that APP abundance perturbs memory through a mechanism that is not affected by evacetrapib. Interestingly, CETP expression alone induced memory impairment, which was not reported to our knowledge thus far (Fig. 3B). However, memory was sustained with evacetrapib, demonstrating a pharmacological approach to prevent memory loss. The double transgenic APPtg/CETPtg mice exhibited memory impairment as well, due to a superadditive effect of both transgenes (*P* = 0.04, two-way ANOVA test). Strikingly, evacetrapib treatment preserved memory function in APPtg/CETPtg mice despite APP abundance (Fig. 3B) showing that CETP inhibition dominates over the APP-mediated memory impairment. This result establishes CETP inhibition as a viable strategy to delay memory impairment. Since female mice showed a pronounced cognitive effect with evacetrapib, and because CETP remodels lipoprotein particles with sex as the major driver of lipoprotein differences, we show further analyses for female mice (and results from male mice are presented in the Appendix). We further assessed memory performance using the Y-maze test, which evaluates spatial memory. No changes, including no impairment in APPtg mice, were observed, indicating that spatial memory remains unaffected at this comparably young age of the mice (Fig. S2A–C) (Kraeuter et al, 2019).

### CETP transgenicity does not exacerbate the amyloidogenic pathway

According to the amyloid hypothesis, APPtg mice develop cognitive impairment due to the generation of Aβ peptides by proteolytic cleavages of APP (Selkoe and Hardy, 2016). To reveal whether CETP activity impacts Aβ production, we quantified Aβ40 and Aβ42 peptides from brain lysates at 21 weeks of age by multiplex ELISA. No significant differences were observed in either soluble or insoluble (plaque-deposited) Aβ species across conditions (Fig. 3E–H). Similar results were found in males (Fig. S3A–D). Thus, we infer that the memory rescue by evacetrapib in APPtg/CETPtg is not due to altered Aβ peptide production. Further, we quantified Aβ peptides from female mouse plasma and found no changes between groups, indicating that evacetrapib did not change the Aβ clearance in our mouse model (Fig. S3E,F). Accordingly, neither plasma to brain Aβ ratios nor brain Aβ42/Aβ40 ratios showed differences (Fig. S3G–J). Likewise, correlation analyses did not find associations between memory and Aβ in our model (Fig. 3I,J). Overall, the absence of significant differences in Aβ production or clearance between evacetrapib- and vehicle-treated mice in the APPtg/CETPtg group shows that evacetrapib preserves memory independent of the amyloidogenic pathway.

### Good memory correlates with high HDL levels

Previous studies have shown that high HDL levels protect from memory decline (Zuliani et al, 2010). Consequently, we correlated memory performance with lipoprotein levels. We found a significant positive correlation of good memory performance with HDL levels in female mice (Fig. 3K). LDL levels showed a negative trend with cognitive performance, though not significant (Fig. 3L). Thus, one explanation for the maintained memory in females could be the high HDL levels.

### CETP expression does not increase cerebral cholesterol synthesis or excretion

**Figure 4:**
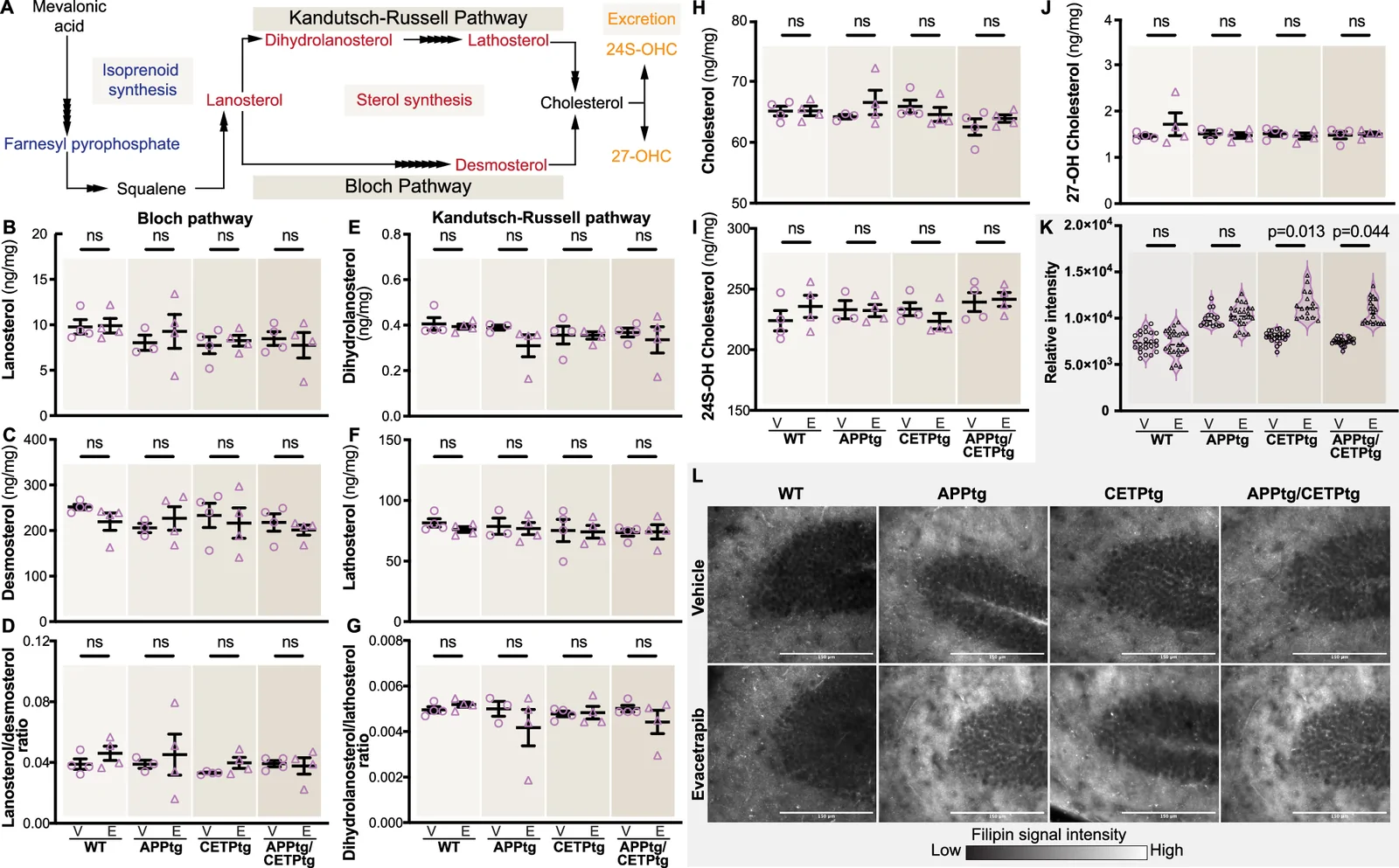
Evacetrapib treatment changes lipid distribution in the hippocampus without affecting overall sterols, sterol precursors, or sterol derivatives in the brain. (**A**) Scheme of the mevalonate pathway, highlighting key steps of isoprenoid and cholesterol synthesis with cholesterol metabolites. (**B**, **C**, **E**, **F**) Lanosterol and desmosterol were detected in brain tissue, along with dihydrolanosterol and lathosterol. (**D**, **G**) Ratios of lanosterol to desmosterol, and 24.25-dihydrolanosterol to lathosterol. (**H**–**J**) Total brain cholesterol was quantified along with cholesterol derivatives 24(S)-OH cholesterol (24S-OHC) and 27-OH cholesterol (27-OHC). (**B**–**J**) Scatter dot plots with indicated mean ± SEM with one point depicting a sample from one mouse, *n* = 3–4, female data points: violet. (**K**, **L**) Filipin III staining in the dentate gyrus of the hippocampus in female mice. Data is shown as violin plots with individual measurements (4–8 technical replicates) from each biological replicate *n* = 3 represented as a data point with the median (full line), first and third quartile (dotted lines, **K**). (**L**) Representative images of sagittal sections of three biological replicates at ×40 magnification. Signal intensity is depicted using a scale bar. Scale 150 µm. (**B**–**K**) Statistical significance for all analyses was measured using two-way ANOVA followed by Tukey’s post hoc test. Source data are available online for this figure.

Most of the brain cholesterol is synthesized locally by oligodendrocytes and astrocytes (Petrov et al, 2016; Rhea and Banks, 2021; Saher et al, 2005). Therefore, cerebral CETP activity may affect local brain cholesterol synthesis independently of plasma cholesterol. We tested the possibility that CETP affects either (1) cholesterol synthesis, (2) cholesterol excretion, or (3) cholesterol distribution in the brain (Fig. 4A). Using mass spectrometry (MS), we measured levels of cholesterol precursors, cholesterol derivatives, and total cholesterol in bulk brain tissue (Fig. 4B–J). First, we examined the abundance of cholesterol precursors lanosterol and desmosterol from the Bloch pathway in female brains and noted no variations between the groups (Fig. 4B–D). Similar results were observed for cholesterol precursors in the Kandutsch–Russell pathway (Fig. 4E–G). The precursor ratios demonstrate that there are no blockages in the respective pathways (Fig. 4D,G). Next, we measured the amount of total cholesterol in the brains of female mice with no changes reported (Fig. 4H). Further, previous studies have shown that defects in prenylation, a process related to cholesterol synthesis, impact memory (Bjorkhem, 2006; Kotti et al, 2006). We investigated mRNA and protein amounts for enzymes involved in the mevalonate/isoprenoid pathway, specifically farnesyl diphosphate synthase (FDPS), farnesyl-diphosphate farnesyltransferase 1 (FDFT1), and geranylgeranyl diphosphate synthase 1 (GGPS1), and found no differences between evacetrapib and vehicle-treated mice, therefore excluding their involvement in memory maintenance in our model (Fig. S4A–G). We conclude that cholesterol synthesis is not affected by CETP activity in our model. To assess cholesterol excretion, cholesterol derivatives 24(S)-hydroxycholesterol (-OH cholesterol) and 27-OH cholesterol were quantified in both brain and plasma, as well as brain cholestanol. However, no differences were found, excluding an effect of CETP on cholesterol excretion (Fig. 4I,J; Fig. S5A). Blood–brain barrier (BBB) integrity was indirectly assessed by measuring diet-derived plant sterols both in the brain and plasma, which showed no changes indicating that the BBB had comparable functionality between groups (Figs. S5B–D and S6A–C). Further, no significant differences were found in plasma and liver samples for cholesterol, cholesterol metabolites, or cholesterol precursors (Figs. S6A–Z and S7A–F). Thus, we concluded that the memory preservation associated with CETP inhibition does not derive from altered cholesterol synthesis or excretion.

### CETP expression changes the lipid distribution in the hippocampus of CETP-expressing mice

To complement the bulk biochemical cholesterol analyses, we next examined the spatial distribution of cholesterol and neutral lipids in brain sections. To detect free cholesterol, we used filipin staining (Gimpl and Gehrig-Burger, 2007). When comparing hippocampal cholesterol content across the different genotypes treated with the vehicle, we observed that APPtg mice exhibited approximately 50% more free cholesterol than WT mice, a finding consistent with previous reports (Chan et al, 2012; Tajima et al, 2013). CETPtg and APPtg/CETPtg mice showed an average increase of ~10% in free cholesterol compared to WT mice, in line with our previous findings (Oestereich et al, 2022). Notably, evacetrapib treatment in CETPtg and APPtg/CETPtg mice further increased total cholesterol levels by up to 60% as compared to WT, specifically in the dentate gyrus (Fig. 4K,L). This result was unexpected as APPtg mice, regardless of treatment, showed higher free cholesterol but exhibited memory impairment. In contrast, evacetrapib-treated CETPtg and APPtg/CETPtg mice maintained their memory despite having higher cholesterol levels. We conclude that the absolute amount of hippocampal cholesterol alone does not determine memory performance. Next, we evaluated the abundance of neutral lipids in the hippocampus using BODIPY665/676 staining, which mostly stains lipid droplets (triglycerides and cholesteryl esters) (Qiu and Simon, 2016). A significant decrease was observed for APPtg/CETPtg mice on evacetrapib as compared to vehicle, showing lower neutral lipid levels compared to all other groups (Fig. S8A,B). The ratio of free cholesterol to neutral lipids was significantly elevated in evacetrapib-treated CETPtg and APPtg/CETPtg mice (Fig. S8C). Thus, while we detected no lipid changes with bulk GC–MS analysis, which was most likely due to the naturally uneven distribution of cholesterol in the brain (Oestereich et al, 2022) and variability during brain dissections. However, the spatial information gained by analyzing brain sections revealed that evacetrapib leads to a significant redistribution of cholesterol and a remodeling of lipid stores, at least in the hippocampus, providing an additional potential explanation for the maintenance of memory by CETP inhibition.

### Expression of genes involved in blood vessel remodeling is altered by CETP inhibition

**Figure 5:**
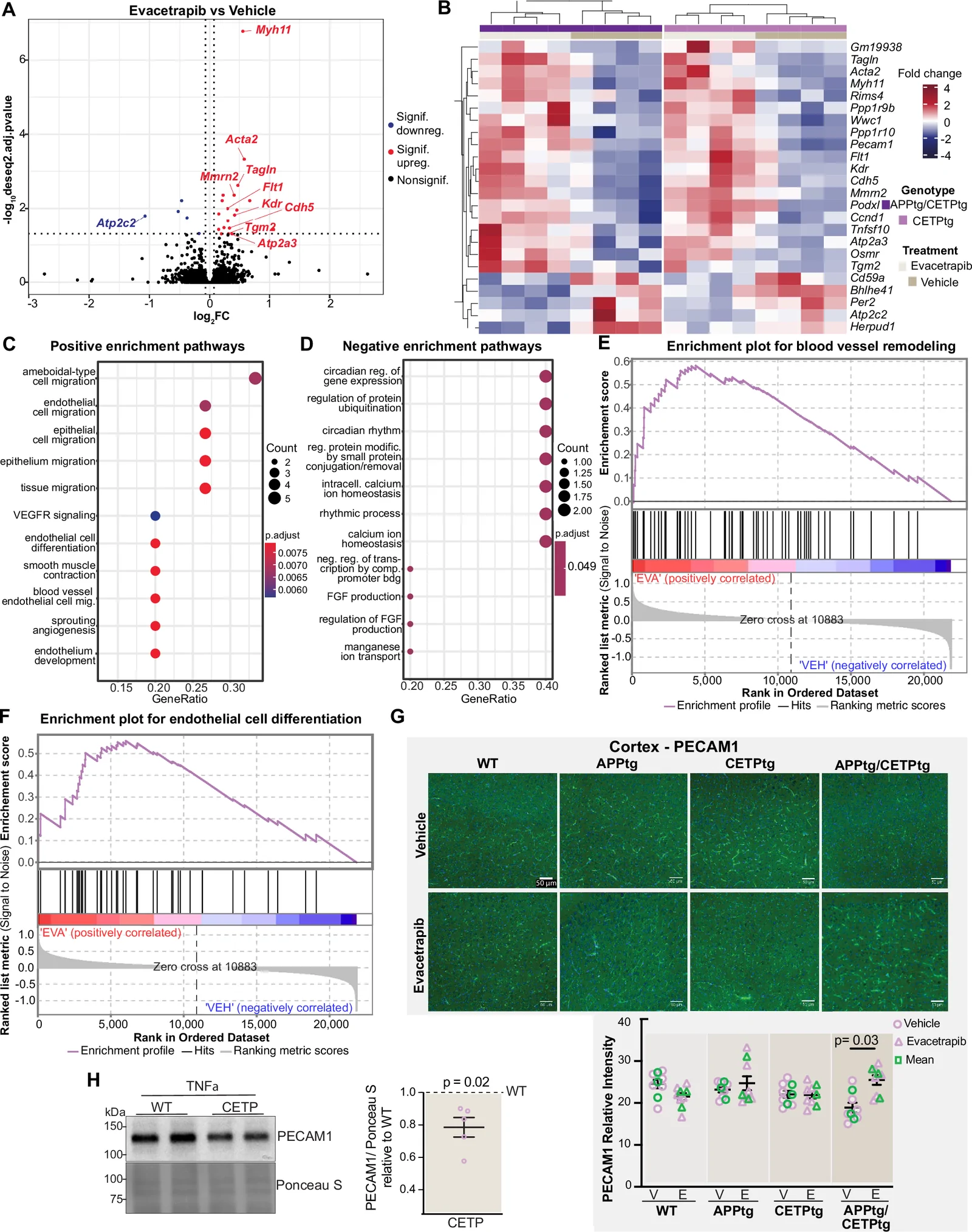
Evacetrapib upregulated genes linked to brain barrier functions. (**A**) Volcano plot of differential gene expression analysis comparing evacetrapib- or vehicle-treated female mice, *n* = 8, by RNAseq. Significant DEGs with |fold change | ≥ 1.05 and *q* value ≤ 0.05 (*P*adj values from DESEQ2, calculated using Benjamini–Hochberg) are shown in red, indicating upregulation, or blue, indicating downregulation. (**B**) Heatmaps and hierarchical clustering of DEGs based on normalized expression. Panel shade approaching red indicates high expression, while blue shade indicates low expression. (**C**, **D**) Bubble plots showing upregulated and downregulated GO enrichment terms of DEGs. The size of the circle at each individual GO term represents the number of genes enriched. The dot shade approaching red indicates higher significance, Benjamini–Hochberg. (**E**, **F**) GSEA enrichment plots using the GO-term database were generated for evacetrapib- or vehicle-treated CETPtg and APPtg/CETPtg female mice, *n* = 4. The analysis runs along the ranked list of genes (left to right) with the running enrichment score (violet line) representing the enrichment of the gene set across the ranked list. (**G**) PECAM1 staining in the cortex of female mice. Representative images of sagittal sections at ×20 magnification; scale 50 µm. Signal intensity was quantified, and plotted with individual measurements (2 sections were stained per mouse (pink symbols) for a total of three biological replicates (mean values indicated in green); Two-way ANOVA followed by Holms–Sidak test on biological replicates in the double transgenic group only. (**H**) PECAM1 immunoblot of bEnd.3 cell lysates after stimulation with TNF-α in treated with WT or CETPtg plasma. Two technical replicates per experiment were averaged for a total of five biological replicates; a one-sample *t* test was performed to compare the effects of CETPtg plasma relative to WT plasma. Scatter dot plot with indicated mean ± SEM. Source data are available online for this figure.

To narrow down the mechanism by which CETP inhibition maintains memory in our mouse model, we performed an unbiased analysis of gene expression comparing evacetrapib- and vehicle-treated groups of CETP-expressing female mice. We identified DEGs and found an upregulation of 19 genes and downregulation of five genes (fold change ≥ |1.05 | , *q* value ≤ 0.05) (Fig. 5A). Hierarchical clustering within each genotype (APPtg/CETPtg and CETPtg) showed that samples cluster by treatment based on their expression profiles across the DEGs, confirming that the effects of evacetrapib on gene expression are consistent within each genotype (Fig. 5B). Out of the 24 DEGs, 11 (*Tagln, Acta2, Myh11, Pecam1, Flt1, Kdr, Cdh5, Mmrn2, Podxl, Tgm2, Cd59a*) are expressed in cells involved in brain barriers, with six (*Pecam1, Flt1, Cdh5, Mmrn2, Podxl, Cd59a*) specifically expressed in brain endothelial cells, four (*Tagln, Acta2, Myh11, Kdr*) in vascular smooth muscle cells (VSMCs) at the choroid plexus, and one (*Tgm2*) expressed both in endothelial cells and VSMCs (Coudert et al, 2022; Pinilla et al, 2021; Sjostedt et al, 2020; Uhlen et al, 2015). Furthermore, although five of the 24 genes (*Ppp1r10, Ccnd1, Tnfsf10, Atp2a3, Osmr*) are not specifically expressed in brain barrier cells, they are documented to play important roles in vascular function (Liu et al, 1997; Lozano-Vidal et al, 2024; Patel et al, 2023; Sato et al, 2006; Tsai et al, 2013). Unbiased GO term analysis revealed a positive enrichment for terms related to blood vessel maintenance and angiogenesis (Fig. 5C,D). In a second approach, we used GSEA analysis to confirm the significant dysregulation of a gene set associated with a particular biological process (Fig. 5E,F). Among the remaining DEGs that are neither expressed in brain barriers nor involved in vascular functions, four genes (*Rims4, Ppp1r9b, Wwc1, Atp2c2*) are associated with neuronal function, memory, and synaptic plasticity, while two genes (*Per2, Herpud1*) are linked to circadian rhythms (Cao et al, 2023; Coudert et al, 2022; Kim et al, 2018; Mi et al, 2017; Newbury et al, 2009; Ryu et al, 2024a; Ryu et al, 2024b; Sjostedt et al, 2020; Uhlen et al, 2015). A principal component analysis was performed for quality assessment (Fig. S9A). Considering that endothelial cells make only about 0.2–7% of all cerebral cells (Kimble et al, 2022), the detection of endothelial-specific genes in this bulk analysis was unexpected. To substantiate this, we quantified PECAM1 protein levels using immunohistochemistry (Fig. 5G). Indeed, double transgenic APPtg/CETPtg mice on evacetrapib exhibited increased PECAM1 signal intensity as compared to the vehicle group (Fig. 5G). To validate the regulation of PECAM1 by CETP activity in vitro, mouse endothelial bEND.3 cells were activated with tumor necrosis factor-α (TNF-α) in the absence of lipoproteins. Then plasma from WT or CETPtg mice was added for 2 h, and PECAM1 was detected by western blot. Plasma from CETPtg mice downregulated PECAM1 compared to WT plasma (Fig. 5H). This finding is consistent with the in vivo observation that PECAM1 increased following CETP inhibition in mouse brains. Given that PECAM1 is a well-established marker of endothelial health (Privratsky and Newman, 2014), these results suggest CETP inhibition maintains endothelial health, which may be a key mechanism underlying the memory benefits of CETP inhibition.

### Association of CETP abundance in the CSF with other proteins in the PREVENT-AD cohort

**Figure 6:**
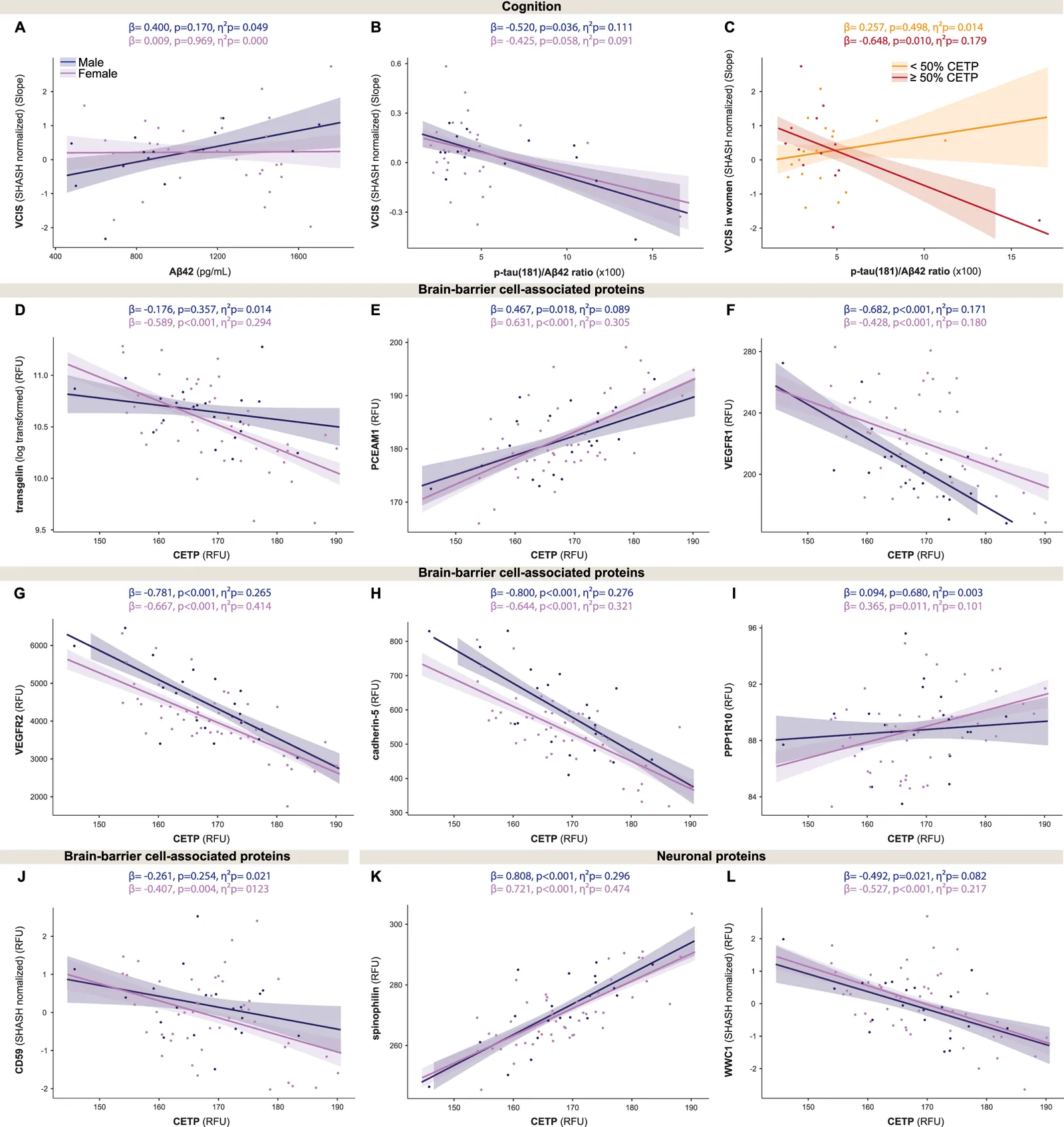
CSF analysis in human subjects corroborates findings from the murine model. SOMAscan proteomics on human CSF of the PREVENT-AD cohort were performed, and relationships were analyzed with cognitive scores and proteins of DEG identified by RNAseq in the mouse model. (**A**, **B**) VCIS slopes in relation to classic AD biomarker Aβ42 (**A**), and p-tau(181)/ Aβ42 ratio (**B**). (**C**) Relationship between three parameters: p-tau(181)/Aβ42, VCIS, and CETP showing either bottom <50% or top ≥50% CETP expressors at baseline in women. (**D**–**J**) CFS proteins from the SOMAscan data. Brain barrier proteins including transgelin, PECAM1, VEGFR1, VEGFR2, cadherin-5, PPP1R10, and CD59 were plotted over CSF CETP at baseline. (**K**, **L**) In addition, the effect of baseline CSF CETP on neuronal protein spinophilin and WWC1 was analyzed. Data are shown as scatter plots with a linear regression line for the main effect of interest. Relationships were analyzed using general linear models (GLMs) and stratified by sex, controlling for participant age, *APOE* ε4 status, and years of education (cognition analyses only). The standard error of the regression line is shown on the plot as full lines surrounding the linear regression. (**A**–**L**) Data points from female subjects are represented in violet and from male subjects in indigo, with the regression line with the corresponding shade, *n* = 19–46. β-coefficient (β), *P* value (*P*), and partial eta squared (ηp^2^) are denoted for each analysis. Source data are available online for this figure.

To assess whether the findings of our mouse model reflect disease changes in humans, we tested if key findings of our mouse model are observed in the CNS of individuals recruited in the PREVENT-AD cohort (Tremblay-Mercier et al, 2021). The PREVENT-AD cohort recruits pre-symptomatic subjects before the appearance of mild cognitive impairment (MCI), but with a strong parental predisposition to developing AD. Over time, some participants (~ 20%) have progressed to MCI, allowing longitudinal cognitive analyses and identifying biochemical changes that occur in the early stages of AD. Cognitive assessment was done with the RBANS over the course of 13 years. The visuospatial constructional index score (VCIS) is one of the RBANS subcategories, combined for a Total RBANS Index Score. Individual trajectories (slopes) of cognitive performance were calculated using cognitive assessment scores from baseline to their most recent follow-up within a set seven-year period. VCIS showed no relationship with Aβ42 alone, suggesting CSF Aβ42 levels do not impact cognition at this stage similarly to APPtg/CETPtg mice (Fig. 6A). However, we observed a significant effect of the CSF p-tau(181)/Aβ42 ratio on VCIS suggesting that this ratio is a strong predictor of worsening cognition and indicates conversion to MCI and potentially AD in these individuals (Fig. 6B). Taking the relationship of the p-tau(181)/Aβ42 ratio over cognition, and separating the top 50% CETP levels from the bottom 50%, the data revealed that low CETP levels were significantly associated with better cognitive performance (VCIS) like in our mouse model (McPherson et al, 1991) (Fig. 6C). Further analyses of the Total Index Score in these asymptomatic subjects failed to detect associations with CSF Aβ42, CETP and p-tau(181)/Aβ42 suggesting that in the presymptomatic stage of the disease, only certain specific cognitive domains such as VCIS are affected by the emerging AD pathology (Fig. S10A–E). Notably, in men, higher CETP levels were associated with improved cognitive performance, though additional data may be required to validate this finding in humans (Fig. S10D,E).

A subset of CSF samples from the PREVENT-AD cohort underwent SOMAscan proteomic analysis at baseline, including CETP protein levels, allowing us to examine the effect of CSF CETP levels on the DEG products identified by the RNA sequencing from mouse brain tissue. Out of 24 DEGs, 23 are protein-coding genes (excluding Gm19938), of which SOMAscan has probes for 19 proteins. 14 proteins were successfully quantified in the CSF of PREVENT-AD participants by SOMAscan (TAGLN (also known as transgelin), PECAM1 (CD31), FLT1 (vascular endothelial growth factor receptor 1, VEGFR1), KDR (vascular endothelial growth factor receptor 2, VEGFR2), CDH5 (cadherin-5), PPP1R10, CD59, PPP1R9B (spinophilin), WWC1, TGM2 (transglutaminase-2), ATP2A3, TNFSF10, OSMR, MMRN2). These proteins were then contrasted with CETP concentrations in the CSF, stratified by sex (Figs. 6D–L and S10F–J). Out of the 14 detected proteins, nine were significantly regulated in relation to CSF CETP levels in women (Fig. 6D–L), showing that changes in our CETP mouse model are relevant in presymptomatic AD women. The cohort demonstrated no significant change in CSF CETP protein levels as a function of the CSF/plasma albumin ratio or the CSF/serum immunoglobulin G (IgG) ratio, showing that the BBB was intact in these asymptomatic subjects (Fig. S10K,L). We conclude that the majority of DEG identified in the mouse model are found with differential abundance in the CSF in at-risk humans as a function of CETP levels, demonstrating that our mouse model replicates aspects of human disease progression.

## Discussion

Conventionally studied in cardiovascular research, CETP has now gained attention in neurodegenerative diseases. Several epidemiological studies have shown that low CETP activity reduces the risk of AD (Barzilai et al, 2006; Barzilai et al, 2003; Chen et al, 2008; Lythgoe et al, 2015; Murphy et al, 2012; Rodriguez et al, 2006; Schmidt et al, 2024; Sun et al, 2013; Sundermann et al, 2016). Our research aimed to determine whether cognitive decline can be prevented by pharmacological CETP inhibition in an AD mouse model, and compared these findings to the at-risk asymptomatic PREVENT-AD human cohort. Focusing on early disease development, we assessed the impact of CETP on cognition, AD biomarkers, as well as cholesterol regulation in relatively young mice (21 weeks of age). Our findings showed that evacetrapib preserved memory in female mice independent of Aβ accumulation, aligning with human data (Aizenstein et al, 2008; Bennett et al, 2006; Jung et al, 2016; Morris et al, 2010). Our findings are supported by the BROADWAY study, a recent AD plasma biomarker study with CETP inhibitor obicetrapib showing that plasma Aβ levels did not change, except in APOE4 carriers (Davidson et al, 2026). As our mouse model does not recapitulate the different APOE isoforms, this may explain why we did not observe changes in Aβ levels. Future studies will have to address the interaction of CETP with APOE isoforms in mouse models. Whether CETP inhibition in the periphery, the brain, or both contributed to memory preservation remains to be determined as well (Bjorkhem and Meaney, 2004; Jiang et al, 1992; Oestereich et al, 2022). Our results, together with the clinical AD biomarker BROADWAY study of CETP inhibition (Davidson et al, 2026), strongly indicate that inhibiting CETP activity during the early stages of AD has significant potential to prevent or delay memory decline (Phenix et al, 2023).

In line with our observations, several other mouse studies investigating lipid changes reported that alterations lead to more profound responses in female mice (Mansukhani et al, 2017; Paigen et al, 1987). Moreover, sex was recently identified as a modifier of CETP effects on lipid biomarkers and cholesterol efflux (Legault et al, 2023). Additionally, sex differences in AD are well-established, with women being twice as likely to develop the condition (Ferretti et al, 2018). Most if not all women affected by AD are post-menopausal, and are particularly at risk, often exhibiting lower HDL and higher LDL levels compared to men (Freedman et al, 2004; Matthews et al, 1989; Mosconi and Brinton, 2018; Solomon et al, 2009). This disparity in risk can be attributed to the influence of estrogen: elevated estrogen levels enhance HDL, and both estrogen and HDL play protective roles in the vasculature, which diminish with the onset of menopause (Beazer and Freeman, 2022; Nie et al, 2022; Tikkanen et al, 1978). Collectively, these observations suggest that CETP inhibition may be beneficial, especially for women after menopause.

A notable observation in our study is that the CETP-mediated effects on the p-tau181/Aβ42 ratio on cognition are weakly associated with Aβ but strongly modulated by sex, indicating that other factors contribute to the causes of memory impairment in AD. Similarly, Tong et al demonstrated in a preclinical study investigating statins that lipid modulation improved cognitive function in 6-month-old APPJ20 mice independent of Aβ or plaque load (Tong et al, 2012). Notably, the demonstrated efficacy of lipid-modifying agents on cognition in humans has primarily been shown in younger subjects (Lin et al, 2015), a phenomenon also observed in mice (Tong et al, 2012; Tong et al, 2009). Data from the PREVENT-AD human cohort investigating the early progression into dementia support these findings, showing no relationship between the AD marker Aβ42 with cognition replicating the findings in our mouse model. Furthermore, in asymptomatic PREVENT-AD subjects, decreased levels of CSF Aβ42 alone did not yet demonstrate a relationship with poorer cognition in either VCIS or overall Total Index Scores slopes, which was previously shown in other studies performed in symptomatic MCI or AD patients (Aizenstein et al, 2008; Bennett et al, 2006; Jung et al, 2016; Morris et al, 2010). While reduced CSF Aβ42 is recognized as a marker of AD, our study focuses on the subjects at their pre-clinical pre-MCI stage (Blennow and Hampel, 2003). Importantly, our results, consistent with our young mouse model, show that cognitive slopes over time, more specifically the prediction of cognitive impairment, were associated with the p-tau181/Aβ42 ratio with high CETP abundance in the CSF in female subjects before disease onset, corroborating the importance of CETP in AD and the relevance of our mouse model to human physiology.

The involvement of lipid metabolism and the potential of pharmacological lipid interventions as disease modulators in dementia is not new (Kivipelto et al, 2005; Olmastroni et al, 2022; Petek et al, 2023; Yadav and Tiwari, 2014). A meta-analysis investigating the impact of statin treatment concluded a potential favorable role of statins on AD and dementia (Olmastroni et al, 2022). While statins may offer overall benefits for AD, their effectiveness is not universal. Women who carry the *APOE ε4* genotype are particularly impacted by the limitations of statins, as they typically exhibit a less favorable response to treatment compared to men (Cai et al, 2021; Wroolie et al, 2020). Furthermore, side effects associated with statin use have previously been noted, such as memory loss and confusion (Jamshidnejad-Tosaramandani et al, 2022). Mechanistically, these adverse effects may stem from the inhibition of 3-Hydroxy-3-Methylglutaryl-CoA Reductase (HMGCR), the rate-limiting enzyme in the mevalonate pathway (Attardo et al, 2022; Goldstein and Brown, 1990; Stancu and Sima, 2001). Although this pathway is primarily responsible for sterol synthesis, it also produces isoprenyl intermediates, which serve as critical posttranslational modifications for the function of small GTPases (Stancu and Sima, 2001). Emerging evidence suggests that prenylated proteins, particularly small GTPases, play significant roles in cognition and AD pathogenesis (Bjorkhem, 2006; Zhang et al, 2019). Consequently, while statins have a positive impact on AD, they are not the ideal choice for standard treatment, especially once the diagnosis is formally established. Therefore, pharmacological CETP inhibition is a promising alternative, particularly for presymptomatic prevention. New-generation CETP inhibitors like evacetrapib and obicetrapib have been proven to be safe, well-tolerated, and effective in lowering CETP activity in cardiovascular clinical trials (Group et al, 2017; Lincoff et al, 2017; Nicholls et al, 2015; Schwartz et al, 2012). Epidemiological studies have highlighted the benefits of low CETP activity, especially at early AD stages in *APOE ε4* carriers, providing a significant advantage over statins (Barzilai et al, 2003; Chen et al, 2008; Lythgoe et al, 2015; Murphy et al, 2012; Rodriguez et al, 2006; Sun et al, 2013; Sundermann et al, 2016). The above-mentioned recent clinical data are the first trial results demonstrating a delay in AD plasma biomarker progression, especially in APOE4 carriers, providing first human evidence of the benefits of CETP inhibition in AD (Davidson et al, 2026). Given that CETP is mainly active only in plasma, adverse effects associated with statins are unlikely to occur with CETP inhibition (Barter et al, 2003; Barter et al, 1982; Tall, 1993). In addition, CETP inhibitors reduce the risk of type 2 diabetes by increasing HDL-cholesterol, which is inversely related to diabetes risk (Dangas et al, 2022; Masson et al, 2018), and notably type 2 diabetes increases the risk for AD (Athanasaki et al, 2022). Ultimately, as outlined in the introduction, decreased CETP activity is linked to various health benefits such as protection from hypercholesterolemia, Parkinson’s disease, amyotrophic lateral sclerosis, sepsis, and cancer, along with AD (Nicholls et al, 2026; Schmidt et al, 2024). Thus, CETP inhibitors as preventive treatments hold significant promise for AD and may additionally offer protective benefits against several other conditions, enhancing their potential as a novel therapeutic approach.

We observed significant but modest transcriptional changes between evacetrapib- and vehicle-treated CETP-expressing mice (24 genes), which could be attributable to using bulk mRNA extracted from total brain tissue. The majority of DEGs are expressed by endothelial cells or VSMCs and are involved in angiogenesis and vascular homeostasis (Coudert et al, 2022; Sjostedt et al, 2020; Uhlen et al, 2015). Notably, single-cell RNA sequencing of the human brain has revealed that CETP is almost exclusively expressed in brain endothelial cells (Siletti et al, 2024). Brain endothelial cells only constitute 0.2–7% of all brain cells, thus the observed changes indicate that evacetrapib has profound impacts on the brain vasculature (Kimble et al, 2022). Leakiness of the blood–brain barrier is another established pathology in AD. However, its absence in our cohort of relatively young mice suggests that we are observing an earlier stage of pathology, whereby more detailed analyses will be needed. Rather, our findings suggest that endothelial dysfunction, which we detect here, precedes overt blood–brain-barrier breakdown. For example, PECAM-1 levels serve as an indicator of endothelial integrity through several pathways (Privratsky & Newman, 2014). Previous mouse studies have linked CETP to endothelial dysfunction through nitric oxide synthase mechanisms, suggesting that CETP inhibition by evacetrapib does, in fact, maintain cerebrovascular integrity (Chu et al, 2016; Wanschel et al, 2021). Further projects will investigate the functionality of endothelial cells and angiogenesis with CETP inhibition. In the PREVENT-AD cohort, our murine results align with the human data, where several endothelial-specific and endothelial-related proteins in the CSF showed significant relationships with CETP levels, especially in women. While these proteins promote vascular stability and angiogenesis, the significance of their levels between CSF and brain tissue remains to be investigated (DeLisser et al, 1997; Liu et al, 1997; Pellicani et al, 2020; Pinilla et al, 2021; Sauteur et al, 2014; Shibuya, 2011; Tsuji-Tamura et al, 2021). Nevertheless, CSF changes for several proteins have been characterized (Kalinowska and Losy, 2006; Kant et al, 2018; Vijayalakshmi et al, 2015; Zhang et al, 2023). In addition, decreased CSF levels of transgelin-2, a protein encoded by a gene paralog to *TAGLN*, and VEGFR2 have been associated with conditions such as AD and amyotrophic lateral sclerosis, while elevated CSF PECAM-1 levels have been reported in multiple sclerosis, emphasizing the importance of endothelial protein changes in neurodegeneration and early AD (Kalinowska and Losy, 2006; Vijayalakshmi et al, 2015; Zhang et al, 2023). The overlap between regulated genes and their protein products suggests that similar mechanisms might be at play in both our mouse model and at-risk asymptomatic humans, emphasizing the role of CETP in cognitive function, endothelial health, and brain barrier functions independent of traditional AD biomarkers.

Limitations of our study include the focus on CETP in an amyloidogenic AD mouse model. Future work could explore the impact of CETP on tau, particularly given the clinical observation that obicetrapib lowers p-tau217 in humans (Davidson et al, 2026). In addition, conducting a longitudinal study would help determine if memory preservation continues after the onset of cognitive impairment, as lipid changes are known to influence AD in the early stages but may have less of an effect as the disease progresses (Lin et al, 2015; Tong et al, 2012; Tong et al, 2009). Similarly, it will be important to investigate if CETP inhibition will benefit memory performance if inhibitors are administered after the onset of cognitive impairment. Furthermore, it is currently unclear whether CETP crosses the blood–brain barrier. While brain and CSF lipids are primarily transported by HDL-like particles, the exact role of CETP in brain lipid transfer remains unclear. Since we did not focus our analyses on cholesteryl esters, further investigation is required to establish if CETP activity alters the cholesteryl ester content or quality in the brain and/or plasma. Other studies observed that cholesteryl esters change during early AD stages and track with the progression of the disease, potentially serving as plasma biomarkers (Cunnane et al, 2012; Proitsi et al, 2015; Tajima et al, 2013). Lastly, diving deeper into the molecular mechanisms of how CETP activity influences the functionality of the vasculature and memory may reveal new insight on how memory becomes impaired in AD.

In summary, these findings position CETP as a highly promising drug target in AD, with significant potential for repurposing CETP inhibitors like evacetrapib or obicetrapib (Davidson et al, 2026). Our research demonstrates that increased CETP expression impairs cognition, while pharmacological inhibition of CETP preserves cognitive function. The beneficial effects of CETP inhibition appear to be two-fold: in the brain, it increases cholesterol levels in the hippocampus potentially maintaining neuronal function; in the periphery, it modifies plasma lipoproteins thereby improving cerebrovascular health. Importantly, we established that our findings in our mouse model are highly relevant to human physiology, enhancing the scientific validity of our work. We assert that prophylactically administered CETP inhibitors such as evacetrapib before disease onset could effectively preserve memory and delay or prevent the progression of AD.

## Methods

**Table:** Reagents and tools table

Reagent/resource	Reference or source	Identifier or catalog number
**Experimental models**		
APP transgenic mice (APP751 Swedish/Indiana mutations, Thy1 promoter)	Dr. Claudio Cuello Lab	N/A
CETP transgenic mice	Jackson Laboratory	Strain #003904
bEnd.3 brain endothelial cell line	ATCC	CRL-2299
**Antibodies**		
Rabbit anti-PECAM1/CD31	Abcam	ab28364
Goat anti-rabbit Alexa Fluor 488	Thermo Fisher Scientific	A-11008
Rabbit anti-FDFT1	Abcam	ab195046
Rabbit anti-FDPS	Abcam	ab189874
Rabbit anti-GGPS1	Abcam	ab167168
Horseradish peroxidase-conjugated anti-rabbit secondary antibody	Promega	W4011
**Oligonucleotides and other sequence-based reagents**		
qPCR primers FDPS	IDT	GGTGGTTCAGTGTCTGCTACGA, CGCCTCATACAGTGCTTTCACC
qPCR primers FDFT1	IDT	GGATGTGACCTCCAAACAGGAC, CAGACCCATTGAGTTGGCACAC
qPCR primers GGPS1	IDT	CACAGGCATTTAATCACTGGCTG, CGTCGGAGCTTTGAACTGTCTTC
qPCR primers GAPDH	IDT	CATCACTGCCACCCAGAAGACTG, ATGCCAGTGAGCTTCCCGTTCAG
qPCR primers RSP18	IDT	CGGAAAATAGCCTTCGCCATCAC, ATCACTCGCTCCACCTCATCCT
**Chemicals, enzymes, and other reagents**		
Evacetrapib (LY2484595)	Selleckchem	S2925
Kolliphor EL	Sigma-Aldrich	C5135-500G
D-(+)-Glucose	Sigma-Aldrich	G7021-1kg
Filipin III	Millipore Sigma	F4767-1MG
BODIPY665/676	Invitrogen	B3932
CETP Activity Assay Kit	Millipore Sigma	MAK106
High-Capacity cDNA Reverse Transcription Kit	Applied Biosystems	4368814
RNeasy Plus Universal Mini Kit	Qiagen	73404
NEBNext Poly(A) mRNA Magnetic Isolation Module	New England Biolabs	E7490
NEBNext Ultra Directional RNA Library Prep Kit	New England Biolabs	E7760
Human Aβ40/Aβ42 Multiplex ELISA Kit (6E10)	Meso Scale Discovery	K15200E
Hoechst 33342	Abcam	ab228551
Teklad Global Diets	Envigo	T.2920X, D16121201
4% Paraformaldehyde	Fisher	AAJ61899AP
Aqueous Fluoroshield mounting medium	Abcam	ab104139
DMEM	Wisent	319-005-cl
FBS	Gibco	12484028
TNF-α	Thermo Fisher	315-01A-50UG
SYBR green qPCR mastermix	BioRad	1725274
**Software**		
GraphPad Prism	GraphPad Software Inc.	v.9, v.10.6.1
Fiji (ImageJ)	National Institutes of Health	1.53
ZEN Pro	Zeiss	v3.0
LAS X	Leica Microsystems	v3.0.14
STAR aligner	Dobin et al	v2.7.8a
Trimmomatic	Bolger et al	v0.39
StringTie	Pertea et al	v1.3.5
DESeq2	Bioconductor	Release 3.19
clusterProfiler	Bioconductor	Release 3.19
GSEA	Broad Institute	v4.3.3
ODLog	Macropod Software	v2.7.2
Picard	Broad Institute	v2.9.0
ggplot2	Wickham, 2016	version 3.5.1
Jamovi	Love et al, 2024	version 2.5
**Other**		
Amersham Imager 600	GE Healthcare Life Sciences	
BioTek Cytation 5 Cell Imaging Multimode Reader	Agilent Technologies	
Illumina NovaSeq X 25B	Illumina	
Leica STELLARIS 5 Confocal Microscope	Leica Microsystems	
Zeiss Axio Observer Automated Microscope	Zeiss	
MESO QuickPlex SQ 1300	Meso Scale Discovery	
Bioanalyzer	Agilent Technologies	

### Animal strains and housing

APPtg mice express a human *APP*751 transgene carrying the double Swedish (K670N/M671L) and Indiana (V717L) mutations under the Thy1 promoter in neurons on a C57BL/6J background and were kindly provided by Dr. Claudio Cuello (Ferretti et al, 2011). The CETPtg strain was purchased from Jackson Laboratory (Jackson Laboratory, Strain #003904) on a C57BL/6J background (Jiang et al, 1992). Husbandry for both strains was heterozygous, and mice were genotyped using Transnetyx. Both strains were crossed to produce CETPtg, APPtg, and double transgenic APPtg/CETPtg mice with WT littermate controls. Heterozygous mice of both sexes were used in this study. Mice were housed in the McGill University Goodman Cancer animal facility. The protocol was approved by the Animal Compliance Office at McGill University (ACO approval #2013-7359) and conducted in compliance with ethical regulations. ARRIVE guidelines have been followed for reporting. Based on pilot experiments, it was estimated that 6–8 mice per group should be sufficient to yield statistical significance in the NOR or Y-maze test.

### Study design

Mice were fed a standard chow from Teklad Global Diets T.2920X (Envigo, USA). The diet was changed at 11 weeks of age for the modified low-fat RD Western diet with 1% (w/w) cholesterol (#D16121201 without choline supplementation, Research Diets, USA) until the end of the trial. Mice were randomly divided into the vehicle group (Kolliphor EL-glucose) and the evacetrapib-treatment group receiving daily intraperitoneal injections at 30 mg/kg for 11 weeks (Cao et al, 2011). Mice had continuous access to food and water. Behavioral tests were performed at week 11. Tissue collection was performed at week 12.

### Drug preparation

Evacetrapib (LY2484595) was purchased from Selleckchem (Selleckchem, USA). The drug was dissolved in one part Kolliphor EL (Sigma-Aldrich, USA) to stabilize the emulsion of the non-polar compound, and then four parts of 50 g/L D-(+)-glucose (Sigma-Aldrich, USA) were added. The drug solution was kept at 4 °C until use.

### Animal exposure and sample collection

Capillary blood collection was performed at week 12 to determine CETP inhibition in the plasma. Post-mortem, blood was collected using cardiac puncture 4 h after the last injection, and plasma was extracted and frozen. For non-perfused mice, the cerebrum was split into four quadrants and frozen in liquid nitrogen. Two lobes of the liver were collected and frozen in liquid nitrogen. Tissues were kept at −80 °C. For phosphate-buffered saline-perfused mice, left brain hemispheres were fixed in ice-cold 4% paraformaldehyde solutions (Fisher, USA) for 48 h followed by storage at 4 °C in sucrose cryoprotective solution until sectioned.

### CETP activity assay

Mouse plasma CETP activity was measured using the CETP activity assay kit (Sigma-Aldrich, USA) using a modified version of the manufacturer’s instructions optimized to a sixfold reduced volume in a half-area microplate (Greiner Bio-One, Austria). Fluorescence intensity was measured (*λ*_ex_ = 465/*λ*_em_ = 535 nm) using the BioTek Cytation 5 Cell Imaging Multimode Reader (Agilent Technologies, USA).

### Plasma lipid analysis

Mouse plasma analysis was conducted by the Diagnostic Laboratory of Comparative Medicine at the Animal Resources Center at McGill University using a panel for cholesterol, triglycerides, and HDL. The Friedewald equation was used to estimate LDL-cholesterol from HDL-cholesterol and triglycerides (Friedewald et al, 1972). VLDL cholesterol was calculated using the Friedewald formula, followed by the equation: VLDL cholesterol = triglycerides/2.2 mmol/l (Obineche et al, 2002).

### Novel object recognition (NOR) test

A modified version of the NOR test based on Ennaceur et al was used at 20-21 weeks of age (Ennaceur and Delacour, 1988). Mice were habituated to handling for 3 days prior to the experiment. The experiment was carried out in a soundproof dark room in an open field arena, and with an infrared video camera recording mouse behavior. On day one, each mouse was habituated to the box for five minutes. On day two, the familiarization phase, each mouse was put into the same box with two identical objects placed at opposite ends of the box at an equal distance from the nearest corner. Mice were allowed to freely explore the two objects for 5 min. On day three, two objects were placed in the box, one identical to the familiarization phase, and the other object was replaced by a novel object. Mice were tested for 5 min while being recorded. For NOR, each mouse and each recording was labeled with a number different from the mouse identification number to prevent bias during NOR, video recording, and analyses; no formal blinding procedure was used. Time spent on each object was quantified using the ODLog (Macropod Software, Australia). Mice that spent less than one second on each object were excluded from the analysis. Objects in this study were previously tested for bias. Equipment was cleaned with 70% ethanol and dried between each test to avoid olfactory bias. The novel object discrimination ratio was calculated as follows:

$${{{\rm{NOR}}}}\; {{{\rm{ratio}}}}( \% )=	\;{{{\rm{time}}}}\; {{{\rm{exploring}}}}\; {{{\rm{the}}}}\; {{{\rm{novel}}}}\; {{{\rm{object}}}}\\ 	/{{{\rm{total}}}}\; {{{\rm{time}}}}\; {{{\rm{spent}}}}\; {{{\rm{on}}}}\; {{{\rm{both}}}}\; {{{\rm{objects}}}}* 100$$

### Y-maze test

An adapted version of the Y-maze test based on the method by Kraeuter et al was performed in the mice at 20-21 weeks of age to assess short-term spatial memory (Kraeuter et al, 2019). Mice were habituated to handling for 3 days prior to the experiment. The experiment was carried out in a soundproof dark room in an open field arena, and with an infrared video camera recording mouse behavior. At the start of the experiment, mice were placed in the middle of the 3-arm maze. Mice were allowed to freely explore the maze for five minutes while being recorded and then placed back in their home cage. The Y-maze and the examiner's PPE were cleaned with 70% ethanol thoroughly and dried before each test to avoid olfactory bias.

Spontaneous alteration was measured as follows:

$${{{\rm{Spontaneous}}}}\; {{{\rm{alteration}}}}( \% )={{{\rm{Number}}}}\; {{{\rm{of}}}}\; {{{\rm{entries}}}}\; {{{\rm{into}}}}\; {{{\rm{a}}}}\; {{{\rm{novel}}}}\; {{{\rm{arm}}}} \\ /({{{\rm{total}}}}\; {{{\rm{alterations}}}}-2)* 100$$

Each arm was denoted by a number (1, 2, or 3). The series of numbers corresponding to each arm entry was noted for each mouse. The first entry was discarded to avoid directional bias by the examiner in the maze. An entry into a novel arm was defined as entering an arm (e.g., arm 1) after having previously entered two different arms (e.g., arm 2 and arm 3) in any order. Mice with fewer than 8 entries into any arms of the maze were excluded from the analysis. The data was analyzed and plotted on GraphPad Prism (GraphPad Software Inc., USA).

### Filipin III staining

Fixed mouse brains were sagittally sectioned at 25 μm thickness using a cryostat (Leica, Germany). The Filipin III staining followed a previous protocol (Oestereich et al, 2022). Sections were mounted on Fisherbrand^TM^ SuperFrost^TM^ (ThermoFisher Scientific, USA) slides using Aqueous Fluoroshield Mounting Medium with DAPI (Abcam, UK). Images were acquired using the widefield Zeiss Axio Observer Fully Automated Inverted Microscope with objective ×20 and the ZENPro software v.3.0 (Zeiss, Germany). For PECAM1/CD31 staining, free-floating sections were permeabilized and blocked for 1 h (PBS, 1% Triton X-100, 5% BSA, 5% normal goat serum). Anti-PECAM1/CD31 (Abcam ab28364, 1:125) was incubated overnight at 4 °C, followed by secondary antibody for 1 h (goat anti-rabbit Alexa Fluor 488, ThermoFisher Scientific A-11008, 1:1000). Sections were stained with Hoechst 33342 for 2 min (Abcam ab228551, 1:5000 in PBS), and mounted. Images were acquired on a Leica STELLARIS 5 confocal laser scanning microscope using a ×20 objective and LAS X software. All images were analyzed with Fiji (ImageJ, USA) using maximum intensity projections.

### BODIPY665/676 staining

For the BODIPY665/676 staining, a similar protocol as for the filipin III was used. The difference is that the 25 μM-thick free-floating slices were incubated in 20 μM BODIPY665/676 in a 0.3% phosphate buffered saline-Triton X solution for 2 h. BODIPY665/676 was chosen over BODIPY493/503 to reduce the autofluorescence from brain slices in the green channel.

### Amyloid-beta (Aβ) ELISA

Human Aβ40 and Aβ42 were analyzed in the mice using an electrochemiluminescence multiplex ELISA using the 6E10 antibody following the manufacturer’s instructions (Meso Scale Discovery, USA). Soluble Aβ was extracted from the brain using dimethylamine, followed by ultracentrifugation. Insoluble fractions were then extracted from the pellet using formic acid, followed by ultracentrifugation to remove debris, and neutralized prior to loading onto the plate. Plasma samples were diluted fourfold. The ELISA was read using the MESO Quickplex SQ 1300 (Meso Scale Discovery, USA).

### Sterol quantification by gas chromatography-mass spectrometry (GC–MS)

Brains and livers were dried in a SpeedVac concentrator (12 mbar, Thermo Scientific, Germany) and weighed. Sterols were extracted using chloroform/methanol (2:1; v:v) followed by alkaline hydrolysis. Concentrations of sialylated sterols were measured following previous methods (Sosic-Jurjevic et al, 2019), see Appendix (Tables S1 and S2).

The non-cholesterol sterols (epicoprostanol, ISTD) and the oxysterols (^2^H_x_-oxysterols, ISTD) were detected by MS-selected ion monitoring (MS-SIM). GC separation and detection of cholesterol, cholestanol, and 5α-cholestane (ISTD) were performed on a DB-XLB column with a 30 m × 0.25 mm i.d. × 0.25 µm film (J&W Scientific Alltech, USA) in a Hewlett-Packard (HP) 6890 series GC system (Agilent Technologies, USA), equipped with a flame ionization detector (FID) using 5α-cholestane as an internal standard (ISTD). Non-cholesterol sterols and authentic and deuterium-labeled oxysterols were separated on a second DB-XLB column in an HP 6890 N Network GC system connected with a direct capillary inlet system to an inert quadruple mass selective detector HP5975B (Agilent Technologies, Germany). Several internal controls for the plasma or tissue extract were used (Table S1). To avoid autoxidation, 50 µL of a 2.6.-di-tert.-butylmethylphenol/methanol solution (Sigma-Aldrich, USA) were added. After saponification with 2 mL of 1 M 95% ethanolic sodium hydroxide solution (Merck, Germany) (60 °C, 1 h) free sterols and oxysterols were extracted three times with 3 mL of cyclohexane each. Organic solvent was evaporated by a nitrogen stream (60 °C). Residues were dissolved in 80 µL n-decane (Merck, Germany). An aliquot (40 µL) was incubated (70 °C, 1 h) in addition to 20 µL of trimethylsilylating (TMSi) reagent-comprising chlortrimethylsilane (Merck, Germany), 1.1.1.3.3.3-hexamethyldisilasane (Sigma-Aldrich, USA), and pyridine (Merck, Germany, 9:3:1) in a GC vial for GC–mass selective detector non-cholesterol and oxysterol analysis. Another aliquot (40 µL) was incubated in addition to 40 µL of the TMSi-reagent and diluted with 300 µL of n-decane in a GC vial for GC–FID cholesterol analysis. In total, 2 µL were injected by automated injection in splitless mode using helium (1 mL/min) for GC–MS-SIM and hydrogen (1 mL/min) for GC–FID analysis (injection temperature 280 °C). The temperature was kept at 150 °C for 3 min, then heated at 20 °C/min, and kept at 290 °C for 34 min. For mass selective detection, electron-impact ionization was applied with 70 eV. MS-SIM was performed by cycling the quadruple mass filter between different m/z values at a rate of 3.7 cycles/s. TMSi derivatives of the non-cholesterol sterols and di-TMSi-derivatives of the oxysterols monitoring compounds and specific m/z were listed (Table S2).

Peak integration was performed manually. Cholesterol and cholestanol were directly quantified by multiplying the ratios of the area under the curve of cholesterol or cholestanol to 5α-cholestane by 50 µg (ISTD amount). Non-cholesterol sterols and oxysterols were quantified by comparing the area under the curve ratios of each to internal standards, using MS-SIM analyses and standard curves for the respective sterols/oxysterols. Identification of all sterols was verified by comparison with the full-scan mass spectra of authentic compounds. Additional qualifiers (characteristic fragment ions) were used for structural identification (*m/z* values not shown).

### RNA extraction and RNA-sequencing analysis

Total RNA was extracted from bulk brain tissue of CETPtg and APPtg/CETPtg mice using the Qiagen RNeasy Plus Universal Mini Kit following the manufacturer's instructions (Qiagen, Germany). RNA integrity number (RIN) factor was measured for samples ranging between 7.3 and 8.6 (Agilent Technologies, USA). mRNA was enriched from 100 to 500 ng total RNA using NEBNext Poly(A) mRNA Magnetic Isolation Module Kit, and libraries were generated using NEBNext Ultra Directional RNA library kit (New England Biolabs, USA). These cDNA libraries were sequenced using the Illumina NovaSeq X 25B sequencer (Illumina, USA), with 150-nucleotide paired-end reads. RNA-seq fastq reads were trimmed using Trimmomatic (version 0.39) to remove low-quality bases and adapters (Bolger et al, 2014). Trimmed reads were aligned to the *mus musculus* 10 (mm10) reference genome using STAR (version 2.7.8a) with default parameters to generate .bam files (Dobin et al, 2013). Alignments were sorted and duplicates were marked using Picard (version 2.0.1). Raw and normalized reads were quantified using HTSeq-count and the StringTie (version 1.3.5), respectively (Pertea et al, 2015). Differentially expressed genes (DEGs) with a maximum adjusted *P* value of 0.05 and at least 1.05 absolute value fold change were identified using DEseq2 (Bioconductor release 3.19). Samples were hierarchically clustered according to their gene expression profiles across the DEGs. Gene expression was normalized by calculating the z-scores of FPKMs within each gene. Clustering was performed by Euclidean distance using Ward’s method.

Gene ontology (GO) terms significantly enriched in the DEGs were determined using clusterProfiler (Bioconductor release 3.19) with a maximum Benjamini–Hochberg adjusted *P* value (*P*.adj) of 0.05 with the biological process, cellular component, and molecular function gene sets. Redundant GO-terms were removed using the simplify function in clusterProfiler. DEGs were analyzed for enrichment in GO ontologies for biological process, cellular component, and molecular function. Gene set enrichment analysis (GSEA) (version 4.3.3) was performed using the normalized read counts produced from DESeq2 (Subramanian et al, 2005). The analysis used default parameters, including 1000 permutations, and the biological process gene sets in the Molecular Signatures Database (MSigDB), showing significant enrichment in the GOBP_BLOOD_VESSEL_REMODELING and GOBP_REGULATION_OF_ENDOTHELIAL_CELL_DIFFERENTIATION sets.

### Cell culture and western blot analysis

The immortalized brain endothelial cell line bEnd.3 was used (ATCC CRL-2299), and were frequently tested to be mycoplasma negative. In total, 300,000 cells were seeded per six-well in Dulbecco’s Modified Eagle Medium (DMEM) media (Wisent), supplemented with 10% fetal bovine serum (FBS, Gibco), and kept at 37 °C, 5% CO_2_ for 48 h. Then, medium was changed to DMEM supplemented with 2% lipoprotein-deficient serum (LPDS) to deplete extracellular lipoproteins, plus 1% female plasma from either WT or CETPtg mice (12–14 weeks of age on 1% cholesterol diet) for 1 h. Then, tumor necrosis factor-α (TNF-α) was added for 2 h. Cells were lysed in lysis buffer (1% NP40, 50 mM Tris, pH 7.4, 150 mM NaCl, 2 mM EDTA, and 2x protease inhibitors). Proteins were separated by SDS-PAGE under reducing conditions (DTT), and detected by western blot using the rabbit anti-CD31/PECAM1 antibody (Abcam, AB28364). Signal intensity was normalized to ponceau S, averaged for 2 technical replicates, and presented as %-decrease relative to WT. For western blots in the Appendix, the primary antibodies were anti- FDFT1 (Abcam, USA, Catalog #ab195046), anti-FDPS (Abcam, USA, Catalog # ab189874), and anti-GGPS1 (Abcam, USA, Catalog # ab167168). Signals were recorded on the Amersham Imager 600 (GE Healthcare Life Sciences, USA).

### qPCR analysis

Reverse transcription from RNA to complementary deoxyribonucleic acid (cDNA) was performed using the Applied Biosystems High-Capacity cDNA Reverse transcription Kit following manufacturer instructions (Life Technologies, USA). Total mRNA from the brain was determined with SYBR Green qPCR mastermix (BioRad, USA) quantification using mouse-specific primers. GAPDH and RSP18 were utilized as internal controls. RIN factor was measured for samples ranging between 7.3 and 8.6 (Agilent Technologies, USA).

Primer sequences:

FDPS:

5’-GGTGGTTCAGTGTCTGCTACGA-3’, 5’-CGCCTCATACAGTGCTTTCACC-3’.

FDFT1:

5’-GGATGTGACCTCCAAACAGGAC-3’, 5’-CAGACCCATTGAGTTGGCACAC-3’.

GGPS1:

5’-CACAGGCATTTAATCACTGGCTG-3’, 5’-CGTCGGAGCTTTGAACTGTCTTC-3’.

GAPDH:

5’-CATCACTGCCACCCAGAAGACTG-3’, 5’ATGCCAGTGAGCTTCCCGTTCAG-3’.

RSP18:

5’-CGGAAAATAGCCTTCGCCATCAC-3’, 5’-ATCACTCGCTCCACCTCATCCT-3’.

### Principal component analysis (PCA)

PCA was performed using the plotPCA function of ggplot2 version 3.5.1 and rlog normalization of gene raw counts (Wickham, 2016).

### Human PREVENT-AD Cohort

The PREVENT-AD longitudinal cohort is composed of cognitively unimpaired individuals who have a first-degree relative with AD and therefore are at a higher risk of developing this dementia (Breitner et al, 2016). The majority of participants were over the age of 60 at recruitment. However, individuals aged 55–59 years were included if they were within 15 years of the onset of their youngest-affected relative’s symptoms. Over 370 participants have been monitored annually for the past 13 years with biochemical, clinical, and cognitive assessments, cerebrospinal fluid (CSF) and blood biomarkers, and neuroimaging modalities (structural and functional magnetic resonance imaging, and amyloid and tau positron emission tomography scans) (Breitner et al, 2016). The protocol was reviewed and ethically approved (IUSMD-02-34). Data used in the preparation of this article were obtained from the PREVENT-AD program (https://douglas.research.mcgill.ca/stop-ad-centre), data release 7.0. A complete listing of the PREVENT-AD Research Group can be found in the PREVENT-AD database (https://preventad.loris.ca/acknowledgements/acknowledgements.php?date=2024-10-01.). Informed consent was obtained from all participants. Analyses and experiments aligned with the principles set out in the WMA Declaration of Helsinki and the Department of Health and Human Services Belmont Report.

### CSF Aß42, total tau (t-tau), and phosphorylated tau (p-tau181) analysis

Lumbar punctures were performed in a subset of volunteers (*n* = 120) in the morning following an overnight fast, with a Sprotte 24-gauge atraumatic needle as described in Tremblay-Mercier et al (2021) (Tremblay-Mercier et al, 2021). CSF was centrifuged for 10 min at room temperature, cells and insoluble material were excluded, and aliquots were stored at –80 °C. AD biomarkers (p-tau181, t-tau, and Aß42) were measured according to procedures developed by the BIOMARKAPD consortium, using validated Innotest enzyme-linked immunosorbent assay kits (Fujirebio, Japan) (Lleó et al, 2019).

### The repeatable battery for the assessment of neuropsychological status (RBANS)

In the PREVENT-AD cohort, cognitive status is assessed annually by the RBANS scale. RBANS was developed with the purpose of detecting and characterizing early dementia in older adults as well as in younger individuals(Randolph et al, 1998). RBANS tests assess five cognitive domains: immediate memory, visuospatial/constructional skills, attention, language, and delayed memory. Scores are calculated individually for each cognitive domain and then combined to provide a total score. The development and validation of the RBANS is described in detail by Randolph et al (Randolph et al, 1998).

### Analysis of human CSF proteomic profile by SOMAscan

The SOMAscan panel measured ~7500 aptamers mapping to ~6600 unique protein targets. Protein measurements are reported in relative fluorescence units (RFU). Initial data normalization procedures were performed by SomaLogic. Briefly, hybridization normalization was performed at the sample level. Aptamers were then divided into three normalization groups: S1, S2, and S3; based on the observed signal-to-noise ratio in technical replicates and samples. This division was done to avoid combining features with different levels of protein signal for additional normalization steps. Median normalization was then performed to remove other assay biases such as protein concentration, pipetting variation, variation in reagent concentrations, and assay timing, among others (Candia et al, 2017). Finally, normalization to a reference was performed on individual samples to account for additional technical variance as well as biological variance. This normalization step was performed using iterative Adaptive Normalization by Maximum Likelihood, a modification of median normalization, until convergence is reached. Additional details on normalization procedures are documented in SomaLogic’s technical note (SomaLogic Operating Co., 2022).

### Statistical analyses for the mouse dataset

Statistical analyses were run using the GraphPad Prism software version 9, where statistical significance was considered *P* < 0.05. A two-way ANOVA was used to evaluate the effects of treatment and genotype in biochemical and behavioral analyses. When significant main effects or interactions were identified, Tukey’s multiple comparison post hoc tests were conducted.

### Statistical analyses for the human SOMAscan dataset

Relationships between CSF protein levels at participants’ baseline visit were analyzed using general linear models (GLMs), adjusting for participant age, *APOE* ε4 status, and sex. Further GLMs were performed to test the interaction between sex and baseline protein levels on the dependent variable. To assess sex-specific effects, analyses were then stratified by sex using simple effects tests, regardless of the interaction term’s statistical significance.

The trajectory of participants’ cognitive status by RBANS was determined by calculating the slope of scores from their baseline visit to their most recent assessment within a seven-year period. GLMs were run to investigate the effect CETP or AD markers (p-tau181, t-tau, Aβ42) in the CSF at baseline on the trajectory of cognition, adjusting for participant age, *APOE* ε4 status, sex, and years of education. GLMs testing the interaction between sex and baseline CETP or AD marker levels in the CSF were performed, followed by sex-stratified simple effects analyses. To assess if baseline CETP and/or sex moderate the effect of baseline CSF p-tau181/Aβ42 levels on cognitive trajectory, additional GLMs were run testing this three-way interaction (Baseline CSF Aβ42 levels x sex x high/low baseline CSF CETP levels), preceded by simple effects analyses stratified by sex and CETP grouping (regardless of the interaction term’s statistical significance).

Statistical analyses were run using the Jamovi software version 2.5, where statistical significance was considered *P* < 0.05 (Gallucci, 2019; Gallucci, 2020; Love et al, 2024; Lüdecke et al, 2020; The R Development Core Team, 2023). For all GLM analyses, if the model’s residuals did not meet the assumption of normal distribution, data were sinh-arcsinh (SHASH) transformed using JMP® Pro version 17.0.0. Robust standard errors (HC3) were used for analyses when the homoskedasticity assumption was not met (Tables S3 and S4).

### Albumin and IgG CSF/plasma ratios as biomarkers of BBB integrity

All analyses were part of the clinical routine diagnostic assessment of the PREVENT-AD participants between February 2008 and September 2017. Albumin and IgG levels in CSF and plasma were analyzed using immunoturbidimetric assays (kits NK004.OPT and NK032.L.OPT) on an Optilite analyser (Thermofisher Scientific, USA) at the department of clinical biochemistry at the McGill University Health Centre.

## Supplementary information

Appendix

Peer Review File

Source data Fig. 1

Source data Fig. 2

Source data Fig. 3

Source data Fig. 4

Source data Fig. 5

Source data Fig. 6

## Data Availability

The RNAseq dataset generated during the current study is available at the Geo repository with accession number GSE314221. The source data of this paper are collected in the following database record: biostudies:S-SCDT-10_1038-S44321-026-00482-w.
