# Supplementary material for: Reducing CETP activity prevents memory decline in an Alzheimer’s disease mouse model
Source: EMBO Mol Med. 2026 Aug 3;18(9):3597–618. doi: 10.1038/s44321-026-00482-w (PMC13562521; doi:10.1038/s44321-026-00482-w)
Supplement: Supplementary file 1 [file 44321_2026_482_MOESM1_ESM.pdf]

APPENDIX for

Reducing CETP activity prevents memory decline in an Alzheimer's disease mouse model

Table of Content

Appendix Figures S1 – S10 ……………………………………Page 2 - 15

Appendix Tables S1 – S4………………………………………..Page 16 - 21

The PREVENT-AD Research Group collaborators……………..Page 22 - 23

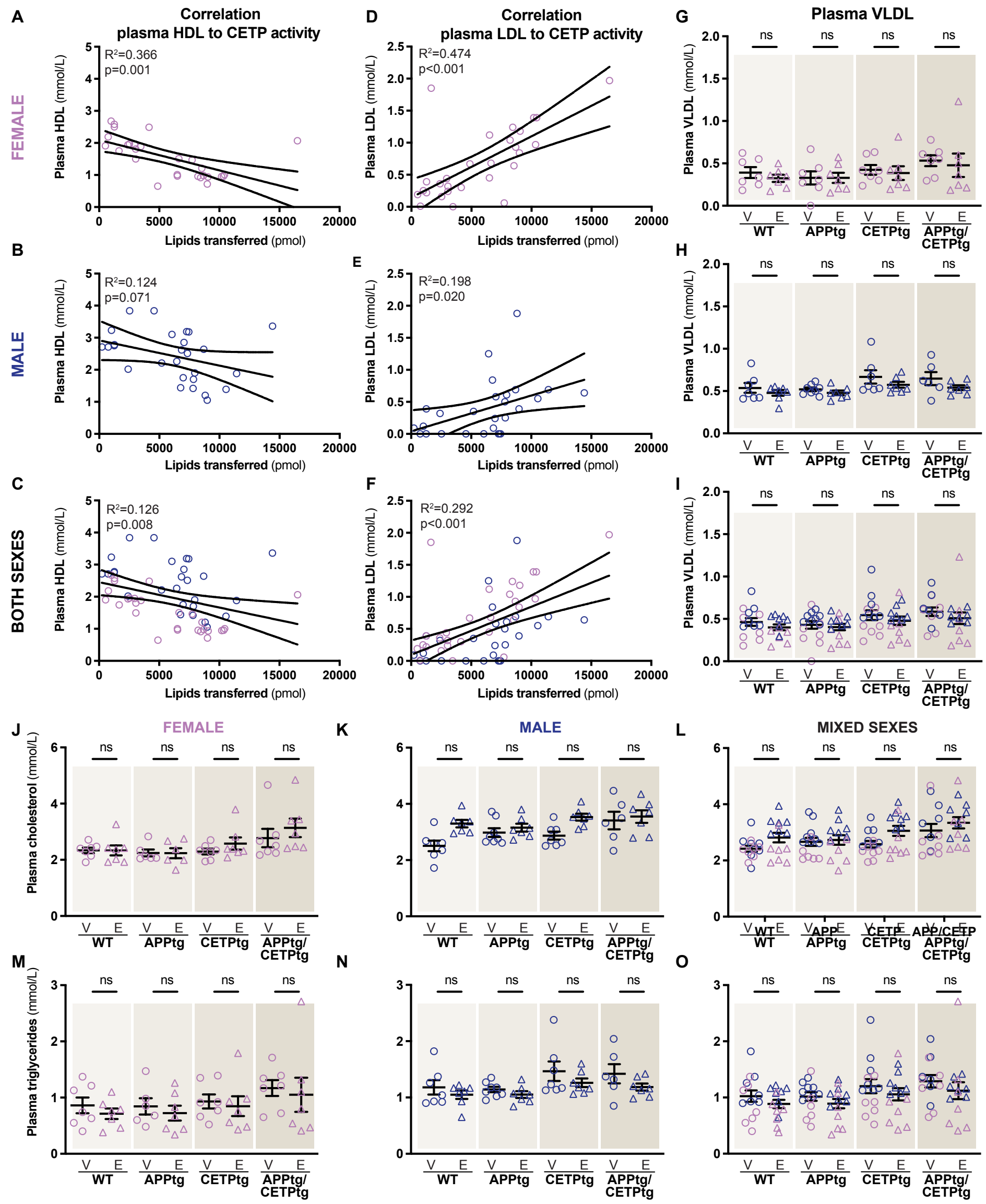

Appendix Figure S1. Correlations between plasma lipoproteins and CETP activity, VLDL levels, as well as total plasma cholesterol and triglycerides. (**A-F**) Linear correlations were calculated to compare CETP activity against HDL and LDL levels, *n=27-28*, in female and male mice, and combined for both sexes. Data is shown as scatter plots with a simple linear regression line. Correlations were analyzed using Pearson's correlation and the 95% confidence interval is shown on the plot as dotted lines. R-squared (R^2) and p-values (p) are indicated for each correlation. (**G-I**) Plasma lipoprotein VLDL, (**J-L**) total cholesterol, and (**M-O**) total triglycerides levels. Data are shown stratified by sex (*n=6-8)* and with sexes combined (*n=12-14)* for visualization. Statistical inference was based on a three-way ANOVA including sex (p<0.0001 for VLDL, cholesterol, and triglycerides), genotype (p=0.01 for VLDL, p<0.0001 for cholesterol, p=0.018 for triglycerides), and treatment (p=0.08 for VLDL, p=0.04 for cholesterol, p=0.046 for

triglycerides), followed by Tukey's post-hoc test. Observed differences were largely driven by sex. (G-O) Data is shown as scatter dot plots with indicated mean ± SEM, each point is data from one mouse. Circles: vehicle (V); triangles: evacetrapib (E); female: violet; male: indigo.

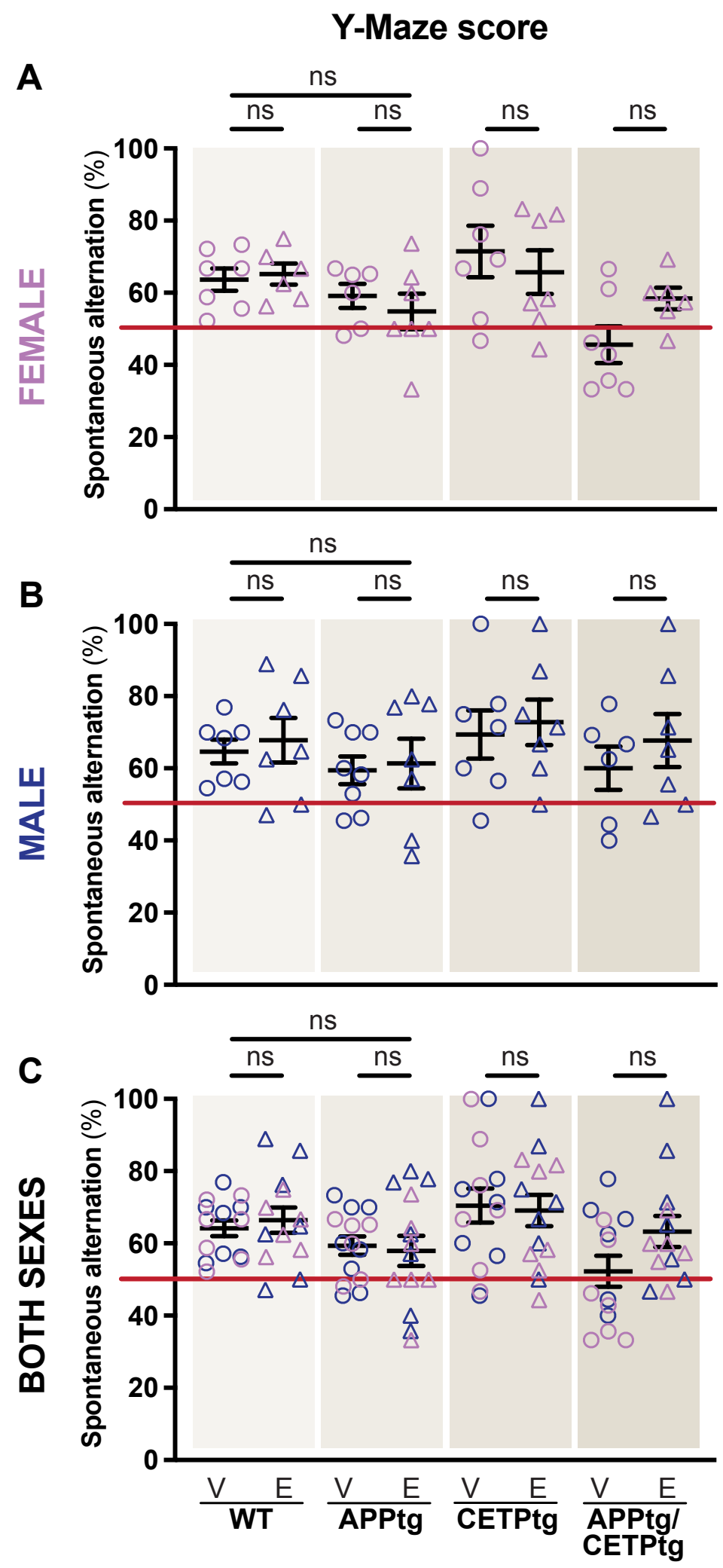

Appendix Figure S2. Y-maze test. Y-maze scores with a red line indicating the 50% alternation ratio at which mice cannot differentiate between a recently entered and a new arm suggesting memory impairment. (**A-B**) Data shown stratified by sex (*n=6-8).* Statistical inference was based on a three-way ANOVA including sex (p=0.06), genotype (p=0.007), and treatment (p=0.39) followed by Tukey's post-hoc test. (**C**) Data from A, B combined for visualization only (*n=12-14)* analyzed by two-way ANOVA and Tukey's post-hoc test. Scatter dot plots with indicated mean ± SEM; Circles: vehicle (V); triangles: evacetrapib (E); female: violet; male: indigo.

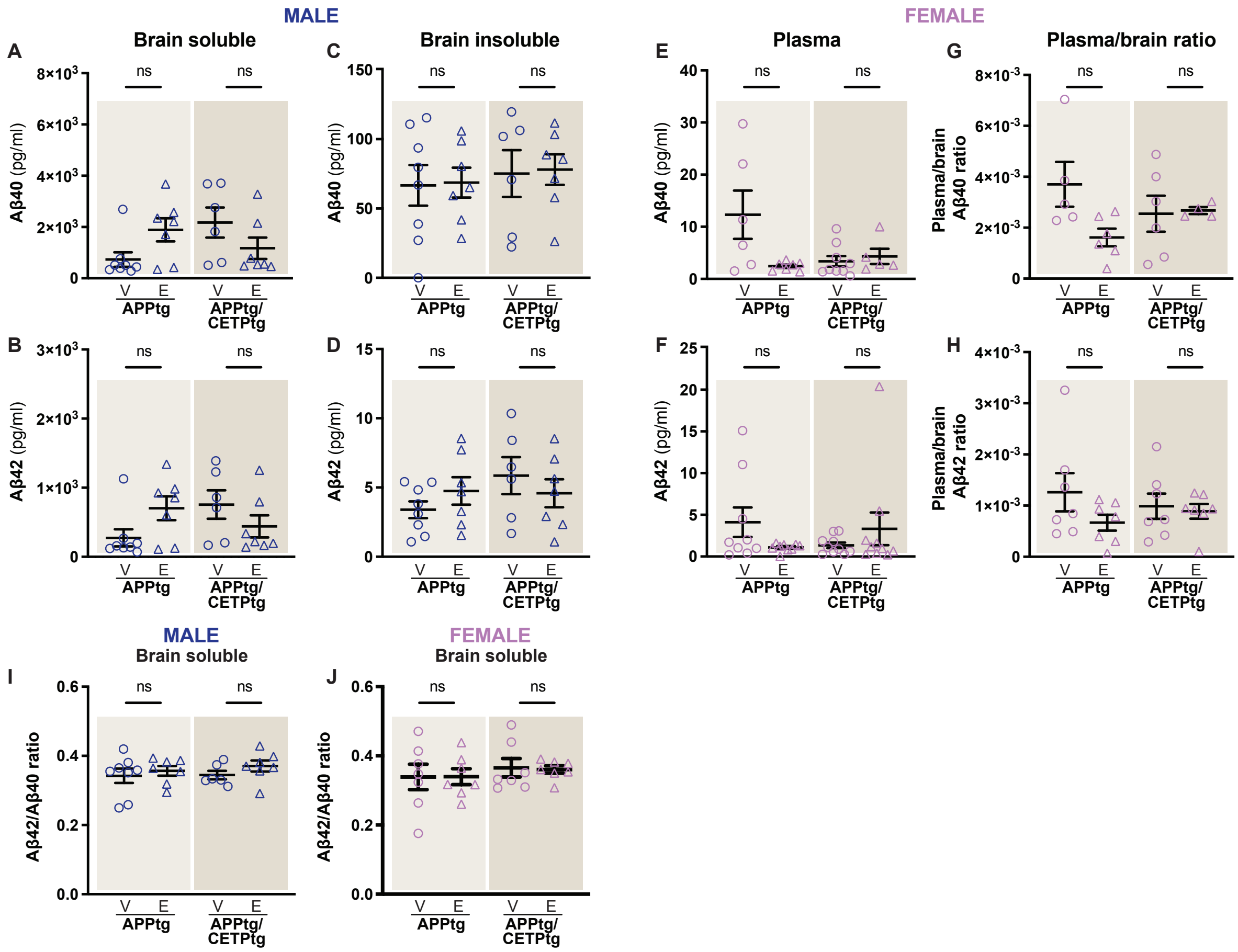

Appendix Figure S3. Aβ40 and Aβ42 quantification in male brains, female plasma, as well as plasma to brain and Aβ42/Aβ40 ratios. (**A-D**) Levels of soluble and insoluble Aβ40 and Aβ42 peptides were quantified in brain homogenates of male mice using ELISA. (**E, F**) Aβ40 and Aβ42 peptides were quantified in plasma of female mice using ELISA. (**G, H**) Plasma to brain ratio for Aβ40 and Aβ42 were plotted to identify potential blockages in excretion. (**I, J**) Aβ40/Aβ42 ratio plotted in male and female for soluble Aβ peptides. (A-J) Data is shown as scatter dot plots with indicated mean ± SEM, *n=6-8*. Statistical significance was measured using two-way ANOVA followed by Tukey's post-hoc test. Circles: vehicle (V); triangles: evacetrapib (E); female: violet; male: indigo.

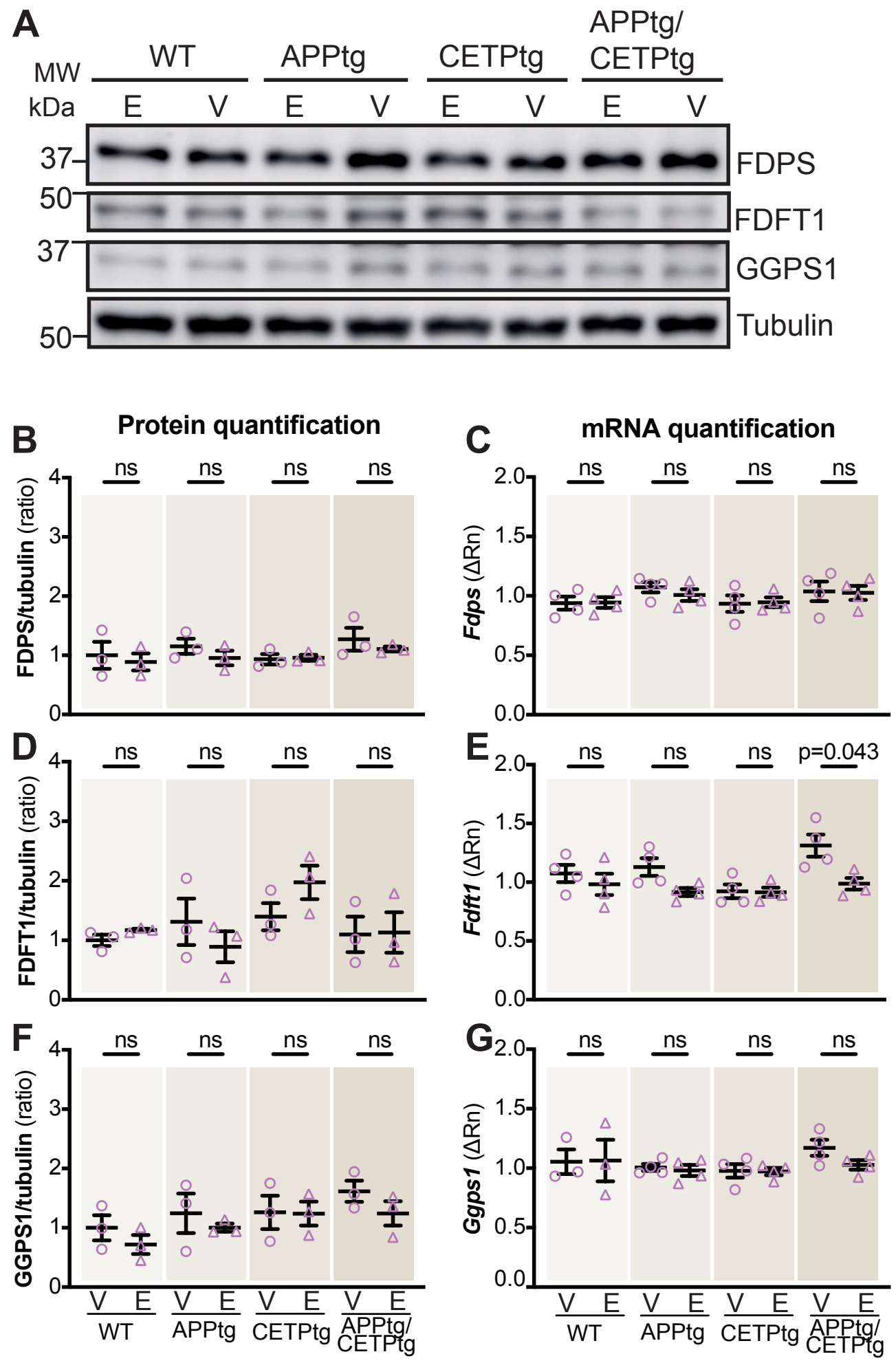

Appendix Figure S4. Enzymes in the mevalonate/isoprenoid pathway in brain tissue. (**A**) Female mouse brain was compared for enzymes FDPS, FDFT1, GGPS1, and tubulin as loading control. Representative images from three biological replicates are shown for each immunoblot panel. Protein quantification from western blot and mRNA quantification was done for (**B, C**) FDPS, (**D, E**) FDFT1, and (**F, G**) GGPS1. MW: molecular weight. mRNA measurements are reported in normalized reporter value (ΔRn). Data is shown as scatter dot plots with indicated mean ± SEM, *n=3-4*. Statistical significance was measured using two-way ANOVA followed by Tukey's post-hoc test. Circles: vehicle (V); triangles: evacetrapib (E); female: violet; male: indigo.

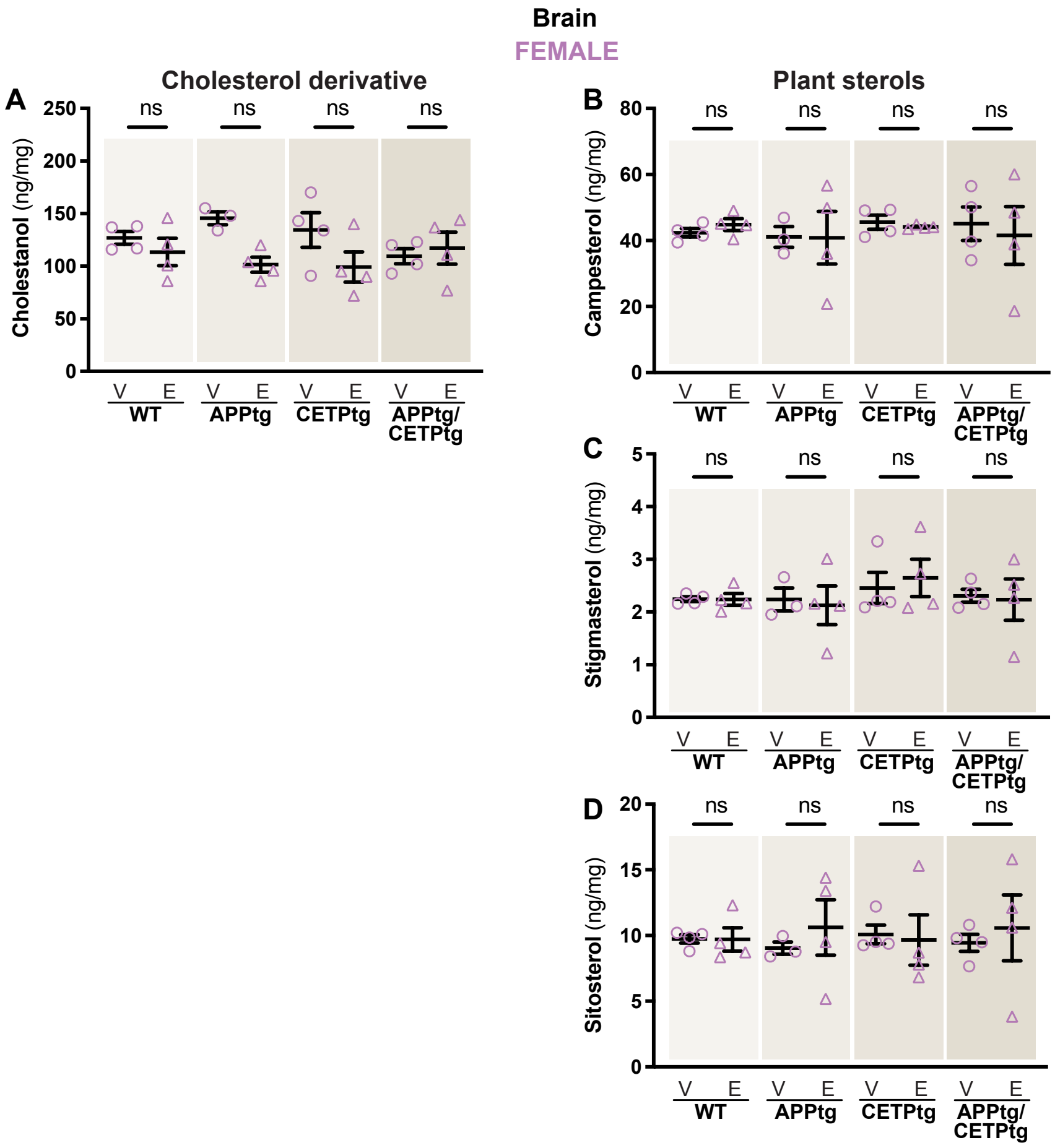

Appendix Figure S5. Food-derived plant sterols and cholesterol derivative in brain tissue. (**A**) Cholestanol and (**B-D**) Plant sterols campesterol, stigmasterol and sitosterol were quantified using GC-MS from brain tissue from female mice. (A-D) Data is shown as scatter dot plots with indicated mean ± SEM, *n=3-4*. Statistical significance was measured using two-way ANOVA followed by Tukey's post-hoc test. Circles: vehicle (V); triangles: evacetrapib (E); female: violet; male: indigo.

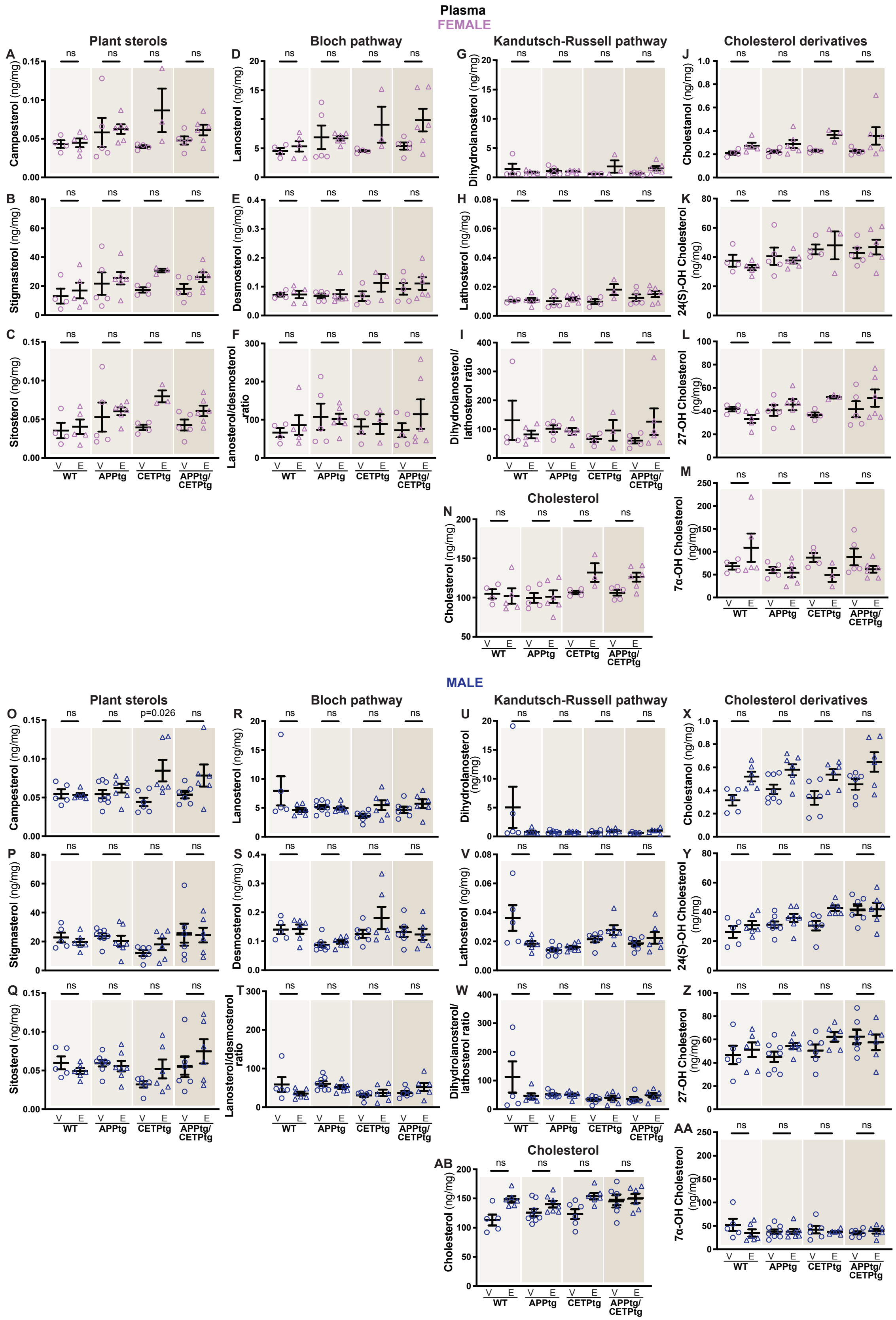

Appendix Figure S6. Plant sterols, cholesterol precursors, cholesterol, and cholesterol derivatives analysis in plasma. Cholesterol analyses from plasma of (**A-M**) female and (**N-Z**)

male mice. Analysis includes plant sterols campesterol, stigmasterol and sitosterol. Cholesterol precursors lanosterol and desmosterol, dehdrolanosterol and lathosterol, total cholesterol and cholesterol derivatives 24(S)-OH cholesterol, 27-OH cholesterol, and 7α-OH cholesterol. (F, I, S, V) Ratios of lanosterol to desmosterol, and 24.25-dihydrolanosterol to lathosterol were plotted. (A-Z) Data is shown as scatter dot plots with indicated mean ± SEM, *n=6-8*. Statistical significance was measured using two-way ANOVA followed by Tukey's post-hoc test. Circles: vehicle (V); triangles: evacetrapib (E); female: violet; male: indigo.

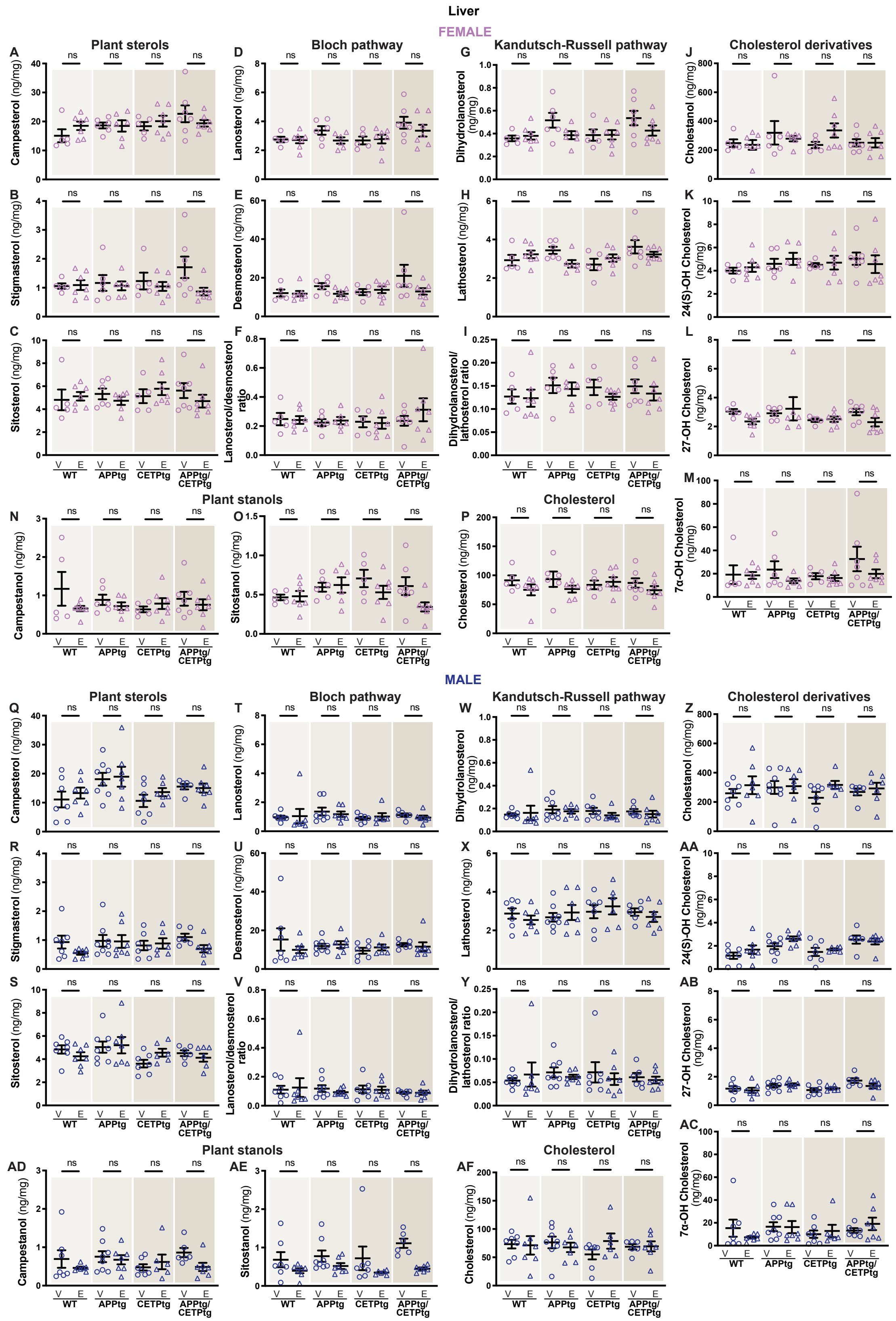

Appendix Figure S7. Plant sterols, plant stanols, cholesterol precursors, cholesterol, and cholesterol derivatives analysis in liver tissue. (**A-AF**) Cholesterol analyses from liver tissue of female (A-P) and male (Q-AF) mice. Analyses include plant sterols campesterol, stigmasterol and

sitosterol. Cholesterol precursors lanosterol and desmosterol, dehdrolanosterol and lathosterol, total cholesterol and cholesterol derivatives 24(S)-OH cholesterol, 27-OH cholesterol, and 7α-OH cholesterol. (F, I, V, Y) Ratios of lanosterol to desmosterol, and 24.25-dihydrolanosterol to lathosterol were plotted to identify potential blockages in the pathway. (A-AF) Data is shown as scatter dot plot with indicated mean ± SEM, *n=6-8*. Statistical significance was measured using two-way ANOVA followed by Tukey's post-hoc test. Circles: vehicle (V); triangles: evacetrapib (E); female: violet; male: indigo.

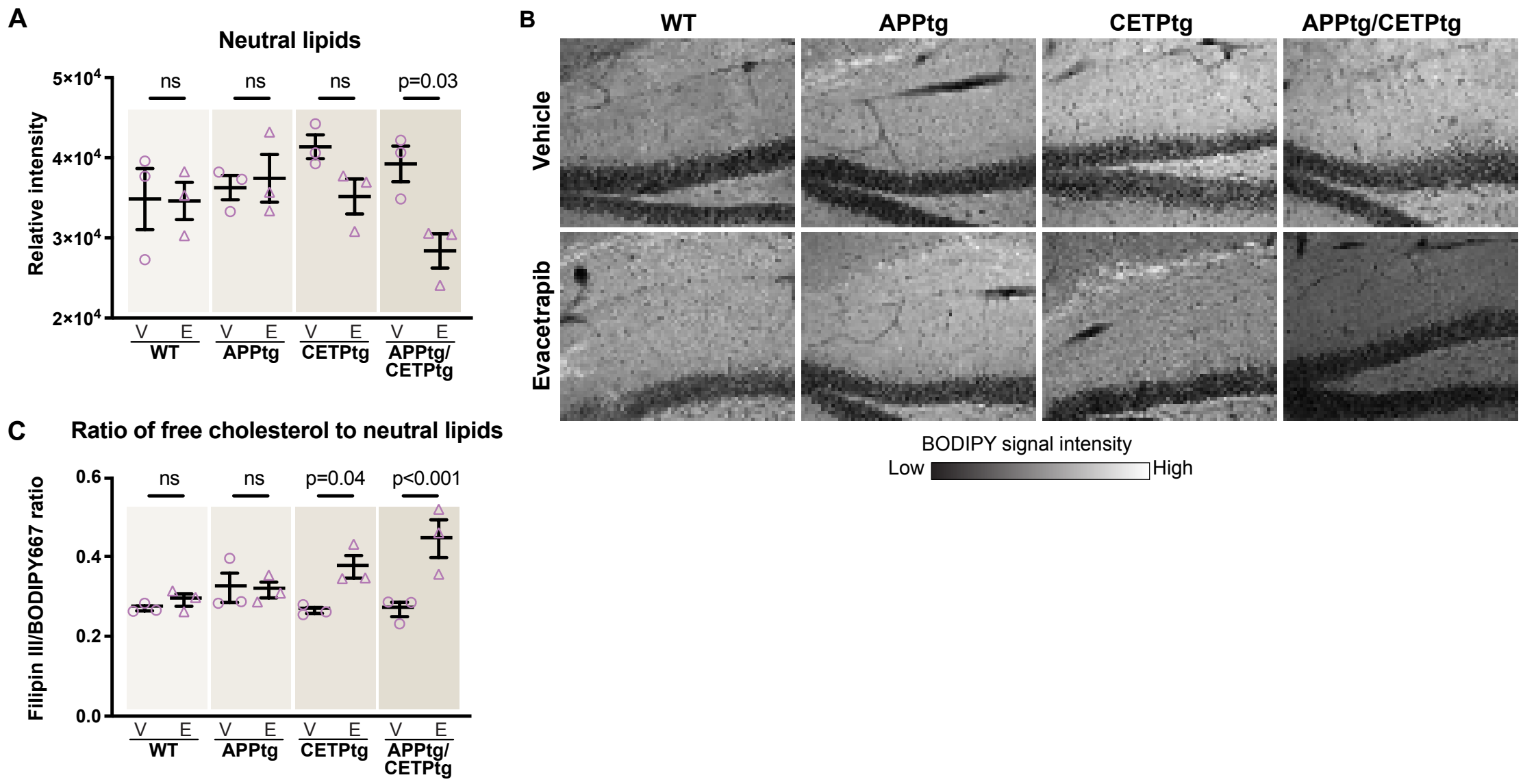

Appendix Figure S8. Neutral lipids distribution in the dentate gyrus region of the hippocampus. (**A, B**) Quantification of BODIPY665/676 staining in the dentage dyrus of the hippocampus of female mice. (**B**) Representative images of sagital sections at 20X magnification in female mouse brains with signal intensity is depicted using a scale bar, 200 µm, with lighter shades representing higher intensity and darker shades indicating lower intensity. Representative images of three biological replicates are shown for each microscopy panel. (**C**) Free cholesterol to neutral lipids signal is represented as a ratio. (A, C) Data is shown as scatter dot plot with indicated mean ± SEM, *n=3*. Statistical significance for all analyses was measured using two-way ANOVA followed by Bonferroni's multiple comparisons test. Circles: vehicle (V); triangles: evacetrapib (E); female: violet; male: indigo.

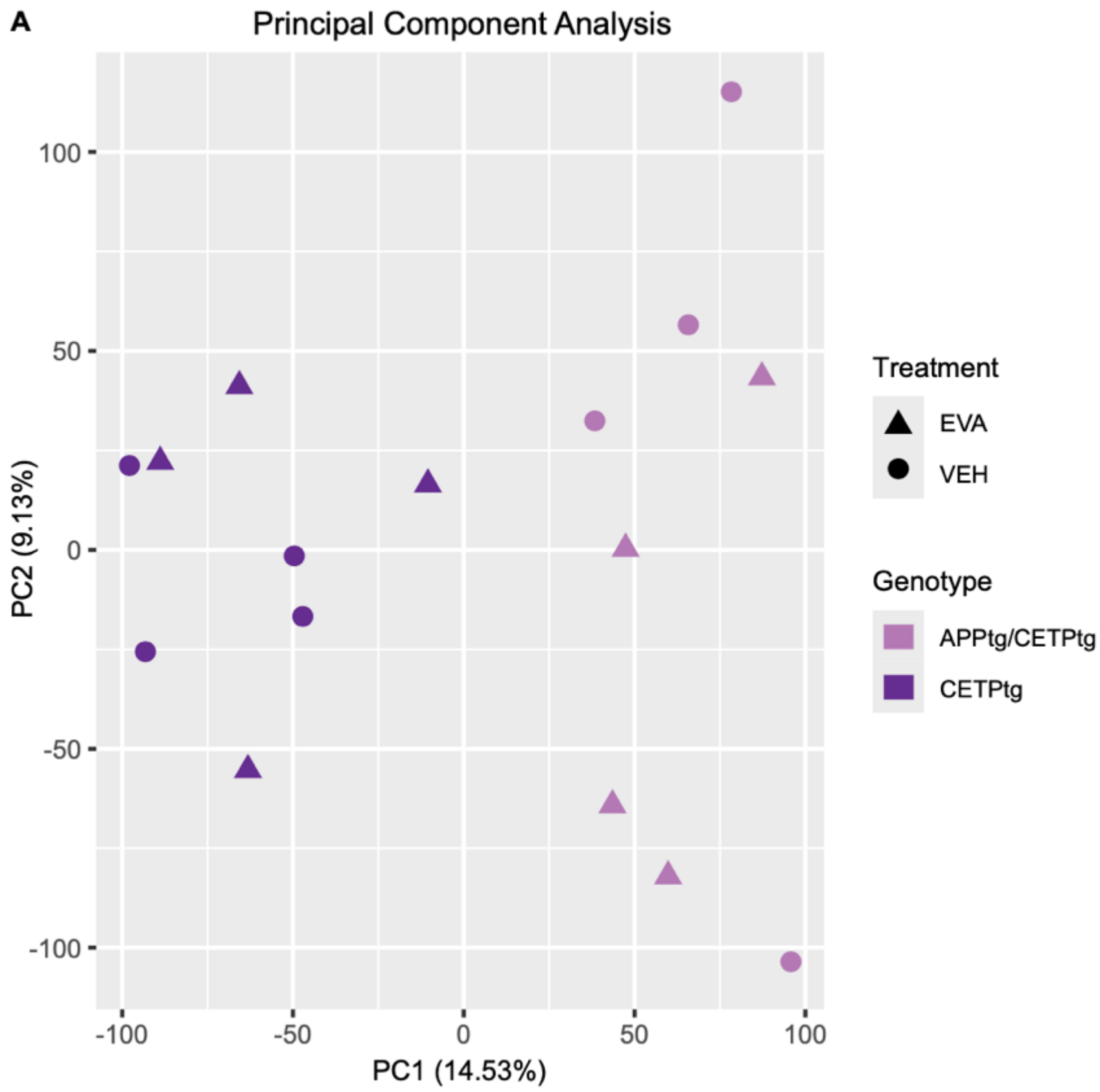

Appendix Figure S9. Principal component analysis. (**A**) PCA analysis. Principal components PC1 and PC2 are shown with 14.53% and 9.13% variance explained, respectively. Circles represent the vehicle condition while triangles denote the evacetrapib condition. Dark purple is set for CETPtg and violet for APPtg/CETPtg replicates.

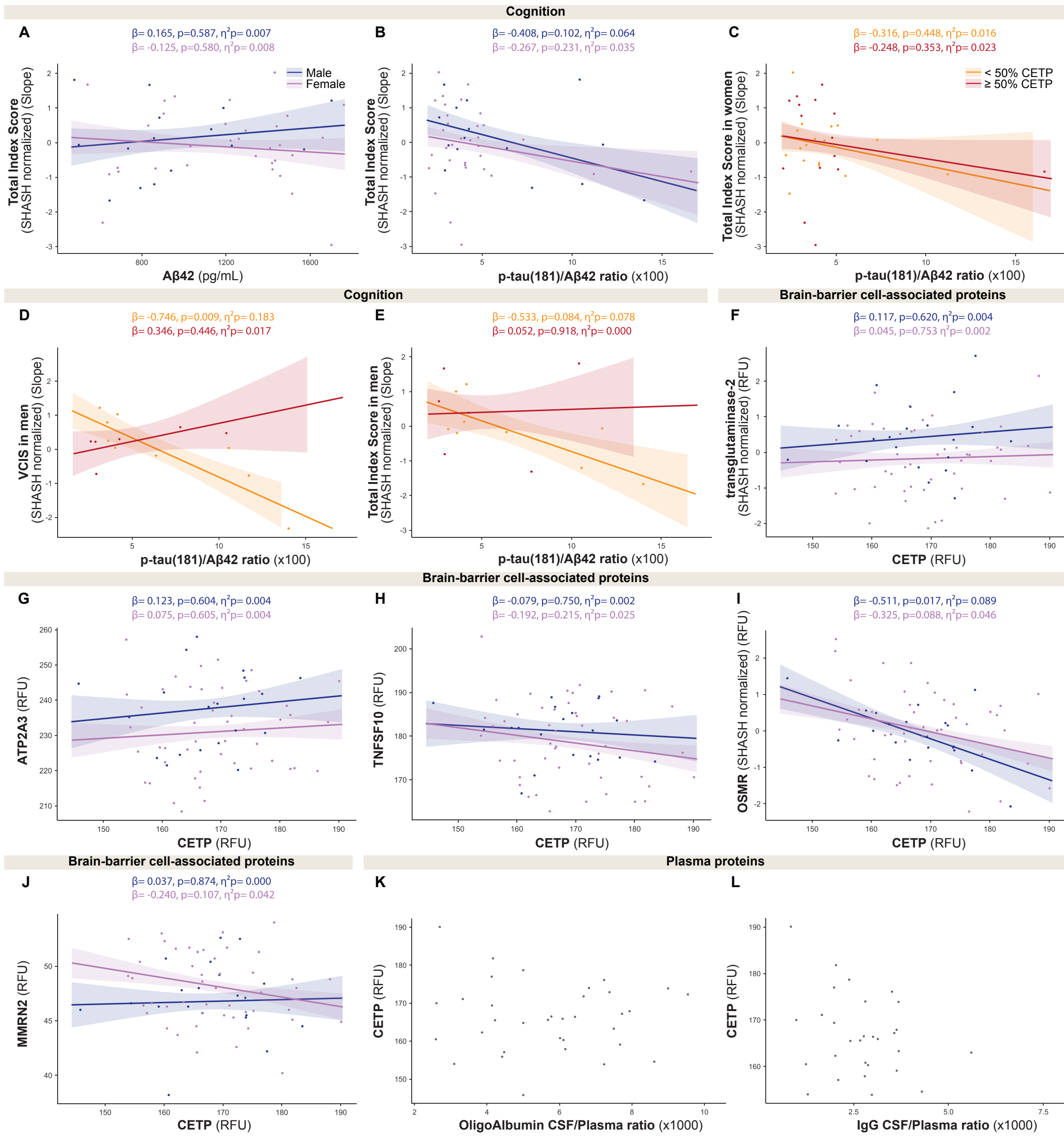

Appendix Figure S10. CSF CETP effects on cognition and CSF proteins. SOMAscan analysis of CSF from human subjects *n=7-46*. (**A-B**) Total Index Score slopes in relation to classic AD biomarker Aβ42, or p-tau(181)/Aβ42 ratio were plotted. (**C-E**) Relation between three parameters, p-tau(181)/Aβ42 , Total Index Score or VCIS, and CETP, showing either bottom < 50% or top ≥ 50% CETP expressors at baseline in men or women. (**F-J**) From the SOMAscan data, brain barrier proteins including transglutaminase-2, ATP2A3, TNFSF10, OSMR, and MMMRN2 were plotted in relation to CSF CETP. (**K, L**) Plasma to brain albumin and IgG ratio were analyzed as indicator of BBB integrity. Data is shown as scatter plots with a simple linear regression line. Relationships were analyzed using GLM stratified by sex, controlling for participant age, *APOE* ε4 status, and

years of education (cognition analyses only). Standard error of the regression line is shown on the plot as full lines surrounding the linear regression. (A-L) Data points from female subjects are represented in violet and from male subjects in indigo, with the regression line with the corresponding shade. β-coefficient (β), p-value (p) and partial eta squared (ηp^2) are denoted for each relationship.

APPENDIX TABLES:

Appendix Table S1. Internal controls for cholesterol, sterol precursors, plant sterols, and oxysterols in 100 µL plasma or to a chloroform/methanol tissue extract (5 mL chloroform/methanol, 2:1, v/v, per 10 mg dry adipose tissue).

Reagent	Provider	Stock
50 µg of 5α-cholestane	Sigma-Aldrich, USA	50 µL of a 1 mg/ml stock solution in cyclohexane from Merck, USA
1 µg of epicoprostanol	Sigma-Aldrich, USA	10 µL of a 100 µg/mL stock solution in cyclohexane
50 ng of racemic [23,23,24,25-2H4]24(R,S)-OH cholesterol	Medical Isotopes, USA	
100 ng 26.26.26.27.27.27-[2H6]-7α-OH cholesterol	Medical Isotopes, USA	
100 ng [16,16,17,20,22-2H5]-(25R)27-OH cholesterol	Sugaris, USA	50 µL of a 2 µg/mL stock solution in toluene from Merck, USA

Appendix Table S2. TMSi derivatives of the non-cholesterol sterols and di-TMSi-derivatives of the oxysterols monitoring compounds and specific mass per change (m/z).

**Compound**	**m/z**
Lathosterol	458 (M^+)
Desmosterol	441 (M^+ -15, M^+ -CH_3)
Lanosterol	393 (M^+ -90-15, M^+ -OTMSi-CH_3)
24.25-dihydrolanosterol	395 (M^+ -90-15, M^+ -OTMSi-CH_3)
Campesterol	472 (M^+)
Sitosterol	486 (M^+)
Stigmasterol	484 (M+)
Campestanol	474 (M^+)
Sitostanol	m/z 488 (M^+)
26.26.26.27.27.27-[$^2H_6$]-7α-OH cholesterol	462 (M^+ -90, M^+-OTMSi)
7α-OH cholesterol	456 (M^+ -90, M^+-OTMSi)
[23,23,24,25-$^2H_4$]24(R,S)-OH cholesterol	416 (M^+ -90-44, M^+ -OTMSi-CD$(CH_3)_2$)
24(S)-OH cholesterol	413 (M^+ -90-43, M^+ -OTMSi-CH$(CH_3)_2$)
[16,16,17,20,22-$^2H_5$]-(25R)27 OH cholesterol	461 (M^+ -90)
(25R)27-OH cholesterol	456 (M^+ -90)

Appendix Table S3. Detailed statistical analyses of PREVENT-AD data in Figure 6.
N= sample size
β= regression coefficient
SE= standard error
p= p-value
β 95% CI= 95% confidence interval for β coefficient
η²p= effect size

Figure 6	Predictor/Interaction	N	β	SE	p	β 95% CI	η²p
**A (VCIS) (SHASH)**	Aβ42	45	0.167	0.001	0.359	[-0.196, 0.529]	0.022
	Aβ42*Sex	45	-0.391	0.001	0.303	[-1.149, 0.366]	0.028
**B (VCIS)**	p-tau(181)/Aβ42 ($x10^2$)	45	-0.468	0.010	0.009	[-0.810, -0.125]	0.164
	p-tau(181)/Aβ42 ($x10^2$) *Sex	45	-0.095	0.018	0.754	[-0.515, 0.705]	0.003
**C (VCIS) (SHASH) (and S10D)**	p-tau(181)/Aβ42 ($x10^2$) *CETP	45	-0.027	0.095	0.930	[-0.649, 0.594]	0.000
	p-tau(181)/Aβ42 ($x10^2$) *Sex*CETP	45	-1.997	0.216	0.007	[-3.416, -0.579]	0.194
**D (TAGLN) (log)**	CETP	68	-0.478	0.004	<0.001	[-0.681, -0.275]	0.261
	CETP*Sex	68	-0.412	0.009	0.068	[-0.855, 0.031]	0.053
**E (PECAM1)**	CETP	67	0.584	0.080	<0.001	[0.378, 0.790]	0.341
	CETP*Sex	67	0.164	0.176	0.472	[-0.288, 0.616]	0.009
**F (VEGFR1)**	CETP	68	-0.496	0.325	<0.001	[-0.695, -0.296]	0.281
	CETP*Sex	68	0.254	0.722	0.256	[-0.189, 0.697]	0.021
**G (VEGFR2)**	CETP	68	-0.697	8.487	<0.001	[-0.869, -0.525]	0.511
	CETP*Sex	68	0.114	18.983	0.555	[-0.270, 0.499]	0.006
**H (CDH5) (ROBUST SE)**	CETP	68	-0.686	1.218	<0.001	[-0.858, -0.514]	0.501
	CETP*Sex	68	0.155	2.493	0.443	[-0.230, 0.540]	0.010
**I (PPP1R10)**	CETP	68	0.292	0.037	0.017	[0.055, 0.530]	0.088
	CETP*Sex	68	0.271	0.082	0.309	[-0.257, 0.799]	0.017

**J (CD59) (SHASH)**	CETP	68	-0.368	0.013	0.003	[-0.603, -0.132]	0.134
	CETP*Sex	68	-0.146	0.029	0.581	[-0.673, 0.381]	0.005
**K (PPP1R9B)**	CETP	68	0.744	0.103	<0.001	[0.580, 0.908]	0.565
	CETP*Sex	68	-0.087	0.231	0.638	[-0.455, 0.281]	0.004
**L (WWC1) (SHASH)**	CETP	68	-0.517	0.012	<0.001	[-0.734, -0.301]	0.266
	CETP*Sex	68	-0.035	0.027	0.887	[-0.520, 0.451]	0.027

**Figure 6**	**Simple Effects**	**Sex**	**N**	**β**	**SE**	**p**	**η²p**
**A (VCIS) (SHASH)**	Aβ42	Male	15	0.400	0.000	0.170	0.049
		Female	30	0.009	0.000	0.969	0.000
**B (VCIS)**	p-tau(181)/Aβ42 ($x10^2$)	Male	15	-0.520	0.014	0.036	0.111
		Female	30	-0.425	0.013	0.058	0.091
**C (VCIS) (SHASH)**	p-tau(181)/Aβ42 ($x10^2$)	Female <50% CETP	19	0.257	0.116	0.498	0.014
		>50% CETP	16	-0.648	0.074	0.010	0.179
**D (TAGLN) (log)**	CETP	Male	20	-0.176	0.007	0.357	0.014
		Female	48	-0.589	0.149	<0.001	0.294
**E (PECAM1)**	CETP	Male	20	0.467	0.095	0.018	0.089
		Female	47	0.631	0.011	<0.001	0.305
**F (VEGFR1)**	CETP	Male	20	-0.682	0.620	<0.001	0.171
		Female	48	-0.428	0.378	<0.001	0.180
**G (VEGFR2)**	CETP	Male	20	-0.781	16.299	<0.001	0.265
		Female	48	-0.667	9.936	<0.001	0.414
**H (CDH5) (ROBUST SE)**	CETP	Male	20	-0.800	2.037	<0.001	0.276
		Female	48	-0.644	1.476	<0.001	0.321
**I (PPP1R10)**	CETP	Male	20	0.094	0.070	0.680	0.003
		Female	48	0.365	0.043	0.011	0.101
**J (CD59) (SHASH)**	CETP	Male	20	-0.261	0.025	0.254	0.021
		Female	48	-0.407	0.015	0.004	0.123
**K (PPP1R9B)**	CETP	Male	20	0.808	0.198	<0.001	0.296
		Female	48	0.721	0.121	<0.001	0.474
**L**	CETP	Male	20	-0.492	0.023	0.021	0.082

**(WWC1) (SHASH)**		Female	48	-0.527	0.014	<0.001	0.217

Appendix Table S4. Detailed statistical analyses of PREVENT-AD data in Appendix Figure 10.

Appendix Figure 10	Predictor/Interaction	N	β	SE	p	β 95% CI	η^2p
**A (RBANS total) (SHASH)**	Aβ42	48	-0.031	0.001	0.863	[-0.396, 0.333]	0.001
	Aβ42*Sex	48	-0.290	0.001	0.441	[-1.042, 0.462]	0.015
**B (RBANS total) (SHASH)**	p-tau(181)/Aβ42 ($x10^2$)	48	-0.329	0.057	0.060	[-0.672, 0.014]	0.082
	p-tau(181)/Aβ42 ($x10^2$) *Sex	48	0.141	0.103	0.652	[-0.484, 0.766]	0.005
**C, E (RBANS total) (SHASH)**	p-tau(181)/Aβ42 ($x10^2$)*CETP	48	0.289	0.102	0.353	[-0.333, 0.912]	0.022
	p-tau(181)/Aβ42 ($x10^2$) *Sex*CETP	48	-0.517	0.259	0.511	[-2.093, 1.060]	0.012
**D (VCIS) (SHASH)**	Refer to Table S3, Figure 6C						
**F (TGM2) (SHASH)**	CETP	68	0.065	0.013	0.599	[-0.180, 0.310]	0.004
	CETP*Sex	68	-0.072	0.030	0.794	[-0.621, 0.477]	0.001
**G (ATP2A3)**	CETP	68	0.088	0.160	0.477	[-0.157, 0.333]	0.008
	CETP*Sex	68	-0.048	0.359	0.861	[-0.598, 0.502]	0.000
**H (TNFSF10) (Robust SE)**	CETP	68	-0.162	0.111	0.186	[-0.407, 0.084]	0.027
	CETP*Sex	68	-0.113	0.268	0.700	[-0.663, 0.438]	0.003
**I (OSMR) (SHASH) (Robust SE)**	CETP	68	-0.375	0.016	0.013	[-0.609, -0.141]	0.140
	CETP*Sex	68	0.186	0.031	0.510	[-0.338, 0.709]	0.008
**J (MMRN2)**	CETP	67	-0.161	0.045	0.200	[-0.409, 0.088]	0.026
	CETP*Sex	67	-0.276	0.099	0.312	[-0.818, 0.266]	0.017
**K (CETP)**	OligoAlbumin CSF/Plasma Ratio ($x10^3$)	35	-0.125	0.829	0.516	[-0.514, 0.263]	0.014

**L (CETP)**	IgG CSF/Plasma Ratio (x10^3)	30	-0.245	1.252	0.262	[-0.685, 0.195]	0.050

**Appendix Figure 10**	**Simple Effects**	**Sex**		**N**	**β**	**SE**	**p**	**η^2p**
**A (RBANS total) (SHASH)**	Aβ42	Male		15	0.165	0.001	0.587	0.007
		Female		33	-0.125	0.001	0.580	0.008
**B (RBANS total) (SHASH)**	p-tau(181)/Aβ42 (x10^2)	Male		15	-0.408	0.081	0.102	0.064
		Female		33	-0.267	0.073	0.231	0.035
**C, E (RBANS total) (SHASH)**	p-tau(181)/Aβ42 (x10^2)	Male	<50% CETP	9	-0.533	0.100	0.084	0.078
			>50% CETP	6	0.052	0.166	0.918	0.000
		Female	<50% CETP	19	-0.316	0.137	0.448	0.016
			>50% CETP	16	-0.248	0.088	0.353	0.023
**D (VCIS) (SHASH)**	p-tau(181)/Aβ42 (x10^2)	Male	<50% CETP	9	-0.746	0.083	0.009	0.183
			>50% CETP	6	0.346	0.139	0.446	0.017
**F (TGM2) (SHASH)**	CETP	Male		20	0.117	0.026	0.620	0.004
		Female		48	0.045	0.016	0.753	0.002
**G (ATP2A3)**	CETP	Male		20	0.123	0.308	0.604	0.004
		Female		48	0.075	0.188	0.605	0.004
**H (TNFSF10) (Robust SE)**	CETP	Male		20	-0.079	0.229	0.750	0.002
		Female		48	-0.192	0.141	0.215	0.025
**I (OSMR) (SHASH) (Robust SE)**	CETP	Male		20	-0.511	0.023	0.017	0.089
		Female		48	-0.325	0.021	0.088	0.046
**J (MMRN2)**	CETP	Male		20	0.037	0.084	0.874	0.000
		Female		47	-0.240	0.054	0.107	0.042

♦The PREVENT-AD Research Group collaborators:

Dr. J. Breitner: Centre for Studies on Prevention of Alzheimer's Disease Centre, Douglas Mental Health University Institute, McGill University, Montreal, QC, Canada john.breitner@mcgill.ca

Dr. J. Poirier : Centre for Studies on Prevention of Alzheimer's Disease Centre, Douglas Mental Health University Institute, McGill University, Montreal, QC, Canada jude.poirier@mcgill.ca

Dr. S. Villeneuve : Centre for Studies on Prevention of Alzheimer's Disease Centre, Douglas Mental Health University Institute, McGill University, Montreal, QC, Canada Sylvia.villeneuve@mcgill.ca

Dr. H. Zetterberg : Department of Psychiatry and Neurochemistry, Institute of Neuroscience & Physiology, the Sahlgrenska Academy at the University of Gothenburg, Mölndal, Sweden, Box 100 S-405 30, henrik.zetterberg@clinchem.gu.se

Dr. K. Blennow : Department of Psychiatry and Neurochemistry, Institute of Neuroscience & Physiology, the Sahlgrenska Academy at the University of Gothenburg, Mölndal, Sweden, Box 100 S-405 30, kaj.blennow@neuro.gu.se

Dr. A.A. Baril: Centre for Studies on Prevention of Alzheimer's Disease Centre, Douglas Mental Health University Institute, McGill University, Montreal, QC, Canada, baril.andreeann@gmail.com

Dr. M. Geddes: Douglas Mental Health University Institute, McGill University, Montreal, QC, Canada, maiya.geddes@mcgill.ca

Dr. S. Ducharme : Centre for Studies on Prevention of Alzheimer's Disease Centre, Douglas Mental Health University Institute, McGill University, Montreal, QC, Canada, simon.ducharme@mcgill.ca

Dr. M. Montembeault: Douglas Mental Health University Institute, McGill University, Montreal, QC, Canada, maxime.montembeault@mcgill.ca

Dr. N. Spreng: Montreal Neurological Institute and Hospital, Montreal, QC, Canada nathan.spreng@mcgill.ca

Dr. A. Pichet-Bessette: Centre de recherche de l'Institut Universitaire de Gériatrie de Montreal, Montreal, QC, Canada; Université de Montréal, Montreal, QC, Canada apichetbinette@gmail.com

Dr. L. Munter: (Department of Pharmacology, McGill University, Montreal, QC, Canada lisa.munter@mcgill.ca

Dr. C. Picard: Centre for Studies on Prevention of Alzheimer's Disease Centre, Douglas Mental Health University Institute, McGill University, Montreal, QC, Canada cynthia.picard@mail.mcgill.ca

Mrs. A. Labonté: Centre for Studies on Prevention of Alzheimer's Disease Centre, Douglas Mental Health University Institute, McGill University, Montreal, QC, Canada anne.labonte@douglas.mcgill.ca

Mr. G. Aumont-Rodrigue: Centre for Studies on Prevention of Alzheimer's Disease Centre, Douglas Mental Health University Institute, McGill University, Montreal, QC, Canada gabriel.aumont-rodrigue@mail.mcgill.ca

Mrs J. Ao: Centre for Studies on Prevention of Alzheimer's Disease Centre, Douglas Mental Health University Institute, McGill University, Montreal, QC, Canada jiarui.ao@mail.mcgill.ca

Mrs. I. Sarty: Centre for Studies on Prevention of Alzheimer's Disease Centre, Douglas Mental Health University Institute, McGill University, Montreal, QC, Canada isabel.sarty@mail.mcgill.ca

Mrs J. Loncke: Centre for Studies on Prevention of Alzheimer's Disease Centre, Douglas Mental Health University Institute, McGill University, Montreal, QC, Canada julia.loncke@mail.mcgill.ca

Dr. A. Poirier: Centre for Studies on Prevention of Alzheimer's Disease Centre, Douglas Mental Health University Institute, McGill University, Montreal, QC, Canada alexandre.poirier@mail.mcgill.ca
