# Supplementary material for: Reducing CETP activity prevents memory decline in an Alzheimer’s disease mouse model
Source: EMBO Mol Med. 2026 Aug 3;18(9):3597–618. doi: 10.1038/s44321-026-00482-w (PMC13562521; doi:10.1038/s44321-026-00482-w)
Supplement: Supplementary file 2 [file 44321_2026_482_MOESM2_ESM.pdf]

EMBO Molecular Medicine

Reducing CETP activity prevents memory decline in an Alzheimer's disease mouse model

Jasmine Phénix, Isabel Sarty, Megan Katz, Antonio Vázquez Cobá, Aarón Marure Rojano, Hannah Nie, Anja Kerksiek, Indie Jerry, Clara Berger, Robert Kiss, Dieter Lütjohann, William Pastor, Judes Poirier, and Lisa Munter

Corresponding author: Lisa Munter (lisa.munter@mcgill.ca)

Review Timeline:

Submission Date:	25th Mar 25
Editorial Decision:	28th May 25
Revision Received:	29th Apr 26
Editorial Decision:	28th May 26
Revision Received:	25th Jun 26
Accepted:	25th Jun 26

Editor: Jingyi Hou

Transaction Report:

(Note: With the exception of the correction of typographical or spelling errors that could be a source of ambiguity, letters and reports are not edited. Depending on transfer agreements, referee reports obtained elsewhere may or may not be included in this compilation. Referee reports are anonymous unless the Referee chooses to sign their reports.)

1st Editorial Decision **28th May 2025**

29th May 2025

Dear Dr. Munter,

Thank you again for submitting your work to EMBO Molecular Medicine. I apologize for the significant delay in the review process, which was due to the late arrival of the referees' reports. We have now received feedback from the two referees who agreed to evaluate your manuscript. As you will see in the reports below, the referees find the manuscript to be of interest. They raise, however, several important points, which should be convincingly addressed in a revision of this work.

I think the referees' recommendations are clear and need not be repeated here. All the issues they raised should be thoroughly addressed to strengthen the mechanistic insights and enhance the overall conclusiveness of the work.

We would welcome the submission of a revised version within six months for further consideration. As you may already know, our editorial policy allows in principle a single round of major revision, and it is therefore essential to provide responses to the referees' comments that are as complete as possible.

EMBO Molecular Medicine has a "scooping protection" policy, whereby similar findings that are published by others during review or revision are not a criterion for rejection. Should you decide to submit a revised version, we do ask that you get in touch after six months if you have not completed it, to update us on the status.

Please also contact us as soon as possible if similar work is published elsewhere. If other work is published, we may not be able to extend the revision period beyond three months.

Please read below for important editorial formatting and consult our author's guidelines for proper formatting of your revised article for EMBO Molecular Medicine.

I look forward to receiving your revised manuscript as soon as possible.

Sincerely,
Jingyi

Jingyi Hou
Senior Editor
EMBO Molecular Medicine

*****

When submitting your revised manuscript, please carefully review the instructions that follow below. We perform an initial quality control of all revised manuscripts before re-review; failure to include requested items will delay the evaluation of your revision.

We require:

1) A .docx formatted version of the manuscript text (including legends for main figures, EV figures and tables). Please make sure that the changes are highlighted to be clearly visible.

2) Individual production quality figure files as .eps, .tif, .jpg (one file per figure). For guidance, download the 'Figure Guide PDF': (https://www.embopress.org/page/journal/17574684/authorguide#figureformat).

3) A .docx formatted letter INCLUDING the reviewers' reports and your detailed point-by-point responses to their comments. As part of the EMBO Press transparent editorial process, the point-by-point response is part of the Review Process File (RPF), which will be published alongside your paper.

4) A complete author checklist, which you can download from our author guidelines (https://www.embopress.org/page/journal/17574684/authorguide#submissionofrevisions). Please insert information in the checklist that is also reflected in the manuscript. The completed author checklist will also be part of the RPF.

5) Please note that all corresponding authors are required to supply an ORCID ID for their name upon submission of a revised manuscript.

6) It is mandatory to include a 'Data Availability' section after the Materials and Methods. Before submitting your revision, primary datasets produced in this study need to be deposited in an appropriate public database, and the accession numbers and database listed under 'Data Availability'. Please remember to provide a reviewer password if the datasets are not yet public (see https://www.embopress.org/page/journal/17574684/authorguide#dataavailability).

In case you have no data that requires deposition in a public database, please state so in this section. Note that the Data Availability Section is restricted to new primary data that are part of this study.

7) For data quantification: please specify the name of the statistical test used to generate error bars and P values, the number (n) of independent experiments (specify technical or biological replicates) underlying each data point and the test used to calculate p-values in each figure legend. The figure legends should contain a basic description of n, P and the test applied. Graphs must include a description of the bars and the error bars (s.d., s.e.m.). See also 'Figure Legend' guidelines: https://www.embopress.org/page/journal/17574684/authorguide#figureformat

8) At EMBO Press we ask authors to provide source data for the main manuscript figures. You will receive a separate email with instructions for providing source data with your revised manuscript, including how to upload and organize the files.

9) Our journal encourages inclusion of *data citations in the reference list* to directly cite datasets that were re-used and obtained from public databases. Data citations in the article text are distinct from normal bibliographical citations and should directly link to the database records from which the data can be accessed. In the main text, data citations are formatted as follows: "Data ref: Smith et al, 2001" or "Data ref: NCBI Sequence Read Archive PRJNA342805, 2017". In the Reference list, data citations must be labeled with "[DATASET]". A data reference must provide the database name, accession number/identifiers and a resolvable link to the landing page from which the data can be accessed at the end of the reference. Further instructions are available at .

10) We replaced Supplementary Information with Expanded View (EV) Figures and Tables that are collapsible/expandable online. A maximum of 5 EV Figures can be typeset. EV Figures should be cited as 'Figure EV1, Figure EV2" etc... in the text and their respective legends should be included in the main text after the legends of regular figures.

- For the figures that you do NOT wish to display as Expanded View figures, they should be bundled together with their legends in a single PDF file called *Appendix*, which should start with a short Table of Content. Appendix figures should be referred to in the main text as: "Appendix Figure S1, Appendix Figure S2" etc.

- Additional Tables/Datasets should be labeled and referred to as Table EV1, Dataset EV1, etc. Legends have to be provided in a separate tab in case of .xls files. Alternatively, the legend can be supplied as a separate text file (README) and zipped together with the Table/Dataset file.

See detailed instructions here:

.

11) The paper explained: EMBO Molecular Medicine articles are accompanied by a summary of the articles to emphasize the major findings in the paper and their medical implications for the non-specialist reader. Please provide a draft summary of your article highlighting

- the medical issue you are addressing,

- the results obtained and

- their clinical impact.

This may be edited to ensure that readers understand the significance and context of the research. Please refer to any of our published articles for an example.

12) Author contributions: You will be asked to provide CRediT (Contributor Role Taxonomy) terms in the submission system. These replace a narrative author contribution section in the manuscript.

13) A Conflict of Interest statement should be provided in the main text.

14) Every published paper now includes a 'Synopsis' to further enhance discoverability. Synopses are displayed on the journal

webpage and are freely accessible to all readers. They include a short stand first (maximum of 300 characters, including space) as well as 2-5 one-sentences bullet points that summarizes the paper. Please write the bullet points to summarize the key NEW findings. They should be designed to be complementary to the abstract - i.e. not repeat the same text. We encourage inclusion of key acronyms and quantitative information (maximum of 30 words / bullet point). Please use the passive voice. Please attach these in a separate file or send them by email, we will incorporate them accordingly.

Please also suggest a visual abstract to illustrate your article as a PNG file 550 px wide x 300-600 px high.

15) All Materials and Methods need to be described in the main text using our 'Structured Methods' format. According to this format, the Methods section includes a Reagents and Tools Table (listing key reagents, experimental models, software and relevant equipment and including their sources and relevant identifiers) followed by a Methods and Protocols section describing the methods, ideally using a step-by-step protocol format. The aim is to facilitate adoption of the methodologies across labs.

Please download and fill our Reagents and Tools Table template (.docx), which you can find in our author guidelines: https://www.embopress.org/page/journal/17574684/authorguide#structuredmethods

When submitting your revised manuscript, please do not include the Reagents and Tools Table in the Methods section of the manuscript but upload it as a separate file choosing the file type "Reagent Table".

EMBO Molecular Medicine has a "scooping protection" policy, whereby similar findings that are published by others during review or revision are not a criterion for rejection. Should you decide to submit a revised version, I do ask that you get in touch after three months if you have not completed it, to update us on the status.

***** Reviewer's comments *****

Referee #1 (Comments on Novelty/Model System for Author):

The medical impact is low unless the authors figure out whether there is a direct impact of CETP expression or inhibition in the CNS. If it is only a peripheral lipid metabolism story, it will not be interesting beyond what we already know about statins. Along the same line, there is not much understanding of why CETP inhibition is protective in the APP mouse model, if the amyloid burden is unchanged. This also limits the impact of the work.

Referee #1 (Remarks for Author):

In this manuscript under consideration, Phenix et al explore the role of cholesteryl ester transfer protein (CETP) in Alzheimer's disease (AD) and in an AD mouse model based on epidemiological studies showing correlations between reduced CETP activity/levels and reduced AD risk. CETP is best characterized in the field of atherosclerosis and has been shown to transfer CE from HDL to LDL, leading to development of small molecule CETP inhibitors (like evacetrapib, which is used in this study). These inhibitors have been largely deprioritized mostly based on evidence they are not superior to statins. However, CETP and their inhibitors have not been investigated in the context of AD models. Because CETP is not expressed in mice, its study requires the use of transgenic models expressing human CETP, which were previously generated in the field. The authors therefore crossed these CETP transgenic mice to an APP mouse model and assessed the impact of evacetrapib on AD relevant endpoints. Their study provides evidence that CETP inhibition confers cognitive benefits based on behavioral studies, while they have no effect on amyloid beta markers assessed biochemically. The authors show some brain cholesterol redistribution based on filipin stains (which label free cholesterol) and neutral lipid stains, possibly assessing CE levels. evacetrapib treatment in CETP transgenic mice caused transcriptomic changes in BBB-related genes. Finally, human CSF proteomic datasets from PREVENT-AD were generally in line with preclinical data. Overall the study makes a case that CETP inhibitors should be considered for AD.

This is an interesting study highlighting a possible role of CETP in AD and a potential clinical use of CETP inhibitors in AD. While the study shows potential benefits of evacetrapib in an AD mouse model, there are still some fundamental questions that need to be addressed for a greater impact.

Main comments:

1. While the authors do a good job assessing the impact of CETP expression and inhibition in mice in the periphery, it is very much unclear whether target engagement in the CNS is required for the beneficial effects of the drug. This is particularly important because the drug used is claimed to be CNS-penetrant. Generally, unbound drug levels are to be measured in the blood and in the brain to understand whether the drug is actually brain-penetrant. At the very least, there should be an attempt to assess target engagement in the CNS. Given the role of CETP in CE metabolism, it seems surprising that this metabolite is not included in the sterol characterization, which is otherwise very thorough. The authors should therefore assess CE levels in

the brain of control and transgenic mice expressing CETP in the presence of absence of mutant APP expression and assess also the impact of evacetrapib. This could be done in the mouse CSF as well, although it may be more technically challenging.

2. The authors do a good job assessing soluble and insoluble Abeta levels in the mouse brain, and based on these analyses, it does not appear that CETP expression or inhibition affects these levels. However, for a study of this scope, it is also important to examine plaque counts and morphology and include assessments of neuronal damage. Specifically, does CETP expression or inhibition affect amyloid plaque size distribution, morphology (e.g. compaction) and neuritic dystrophy?

3. Related to the previous point, how is CETP inhibition beneficial in the APP mouse model if plaques are unaffected? Is synaptic loss rescued? Are microglia or inflammatory responses affected in any ways? Are the neutral lipid stains reduced by CETP inhibition restricted to microglia?

Minor comments:

4. Growing evidence suggests brain cholesteryl esters are linked to AD and AD models (both amyloid and tau); this is not discussed in the manuscript and perhaps a missed opportunity to build a case for the potential significance of the CETP pathway. Perhaps the authors should do a more thorough job discussing this evidence in the introduction and discussion sections.

5. While the physiological function of CETP is well established in peripheral lipid metabolism, it is unclear, at least to this reviewer, what CETP might do in the CSF/ISF. As the authors are likely aware of, there are HDL-like particles in the CSF and their size is generally larger than peripheral HDL lipoproteins. What CE transfer reactions do the authors hypothesize might be mediated by CETP? They should comment on this or at the very describe the unknown as a key question.

Referee #2 (Comments on Novelty/Model System for Author):

My notes on statistics are noted in my review. This should be addressed and/or clarified but not a major concern.

Referee #2 (Remarks for Author):

This study by Phénix explores the preclinical use of the CETP inhibitor evacetrapib on Alzheimer's disease mouse model. Since mice don't express CEPT, they crossed a CEPT expressing mouse with APP mouse model of amyloidogenesis. They discovered that evacetrapib effectively altered plasma lipoprotein profile in favour of the protective HDL particles. This drug also improved memory in females. They conclude this is due to a redistribution of lipids in the brain without any leakage of the blood-brain barrier, Finally, they performed a proteomic analysis and confirmed that their model system recapitulates early in the disease progression, and that many alterations in proteins converge on the vasculature.

I found this study very interesting. The rationale outlining the reasons why CEPT is an ideal target for Alzheimer's disease therapy is extremely compelling. And their data supports this as a promising target for therapy in early stages of the disease to prevent or limit its progression. Overall, I am quite enthusiastic about the study. Below I have outlined some points to clarify before the paper would be ready for publication:

1. Is expression of CETP in the CETPtg line sufficient to increase risk of cognitive decline in aged animals? Perhaps previous studies have looked at this? If so, it would be useful information to include when the introducing the animal lines.

2. It would be helpful to mention that in the CETPtg, that CETP is under the expression of its endogenous promoter. This is important given the role peripheral lipoproteins can have on cognition. This information could prevent confusion as to what can and cannot be concluded from the experiments.

3. It would be helpful to better describe the relevance of the 1% cholesterol diet. How much cholesterol is in the regular chow? Is 1% associated with any notable health outcomes in the mice?

4. I find the description of Figure 2 confusing. In figure 2, the authors claim that CEPT expression increased LDL and decreased HDL in vehicle treated CEPT-expressing mice compared to control. But go on to clarify that is only in the female mice. But it doesn't seem like this comparison was made using statistics as they only show the comparisons for vehicle vs evacetrapib. Looking at the graphs, I do not see a difference between plasma HDL in control vs CETP mice in the females. (The correlation graphs in supplemental Fig 1 look good but answer a slightly different question.)

5. I have the same issue with Figure 3. I can see the effect, that the APP, CETP and APP-CETP mice don't discriminate as well as the WT. But I only see the comparisons being made between the vehicle and evacetrapib in the females, which indeed is convincing.

6. I find it curious that evacetrapib shows a slight but nonsignificant decrease in soluble Abeta given that CEPT is not expressed (Fig3E and G). Could this be due to a slightly under powered analysis? Could evacetrapib have off-target effects. Seeing this does not diminish my enthusiasm for the study, as the results are very promising from a preclinical standpoint. But perhaps something to consider from a fundamental perspective.

7. Fig 4. Why do you think you see more hippocampal cholesterol with filipin staining but not in the whole brain? Could this be validated by dissecting out the hippocampus and looking at cholesterol levels biochemically?
8. In figure S8 the changes in neutral lipids (or lack thereof) seems underpowered. It seems like evacetrapib does in fact lower neutral lipids in S8A.

9. In Figure S8, the authors look at the intensity of BODIPY665/676 to analyze neutral lipids. This lipid dye difficult to use in brain tissue because it can often stain other lipid species non-specifically. It also doesn't distinguish between TAGs and CEs. The authors mentioned they don't detect any changes with bulk GC-MS. I think it would strengthen the manuscript to include the omics data for TAGs and CEs separately.

10. I find it a very surprising that you have alterations in genes converging on the blood brain barrier and yet no leakiness. Especially given the defects in the BBB in later stages of Alzheimer's disease. It could be interesting to discuss this direction a little more.

Point-by-point response to the reviewer's comments

In the following, we address the reviewer's concerns point-by-point. Based on the reviewer's comments, we have gained additional, new data in support of our conclusions strengthening overall our manuscript, for which we would like to thank the reviewers and for their time and commitment providing us with their feedback.

Referee #1 (Comments on Novelty/Model System for Author):
The medical impact is low unless the authors figure out whether there is a direct impact of CETP expression or inhibition in the CNS. If it is only a peripheral lipid metabolism story, it will not be interesting beyond what we already know about statins. Along the same line, there is not much understanding of why CETP inhibition is protective in the APP mouse model, if the amyloid burden is unchanged. This also limits the impact of the work.

We respectfully disagree with this concern of reviewer 1. The strongest argument for the potential impact of CETP inhibition in the prevention of Alzheimer's disease was provided during the revision process by the company New Amsterdam Pharma (Davidson et al., *J Prev Alzheimers Dis* 2026; PMID: 41109840, (1)). In a pre-specified substudy of the BROADWAY trial, patients received the CETP inhibitor obicetrapib versus placebo. AD plasma biomarker analyses revealed that obicetrapib treatment lowered p-tau217 across all subjects, and with strongest effects in APOE4 carriers (see copied figure from their paper in **A**). When focusing on APOE4 carriers only, all AD plasma biomarkers decreased (see copied figure in **B**), suggesting that CETP inhibition will be particularly effective in APOE4 carriers, which will have profound effects on the Alzheimer's population as over 60% of Alzheimer's patients carry at least one *APOE4* allele. CETP inhibition has demonstrated beneficial effects in humans, is superior to statins, and does not at all resemble statins (see below). CETP inhibition as a prevention strategy for Alzheimer's disease has huge medical potential.

Regarding the comparison of statins to evacetrapib: It is important to note CETP is a superior drug target as compared to HMGCR: **1)** While polymorphisms for both, HMGCR and CETP, are known to associate with human traits, only human subjects with a complete loss of CETP are known (2, 3), showing that CETP is a safer drug target than HMGCR since its complete loss is not detrimental. **2)** HMGCR is ubiquitously expressed, while CETP shows a restricted expression pattern: CETP is predominantly expressed by the liver and active in the blood stream, showing that targeting CETP will have better selectivity than HMGCR. Drugs targeting CETP will be superior in avoiding side effects as compared to drugs targeting HMGCR. The side effects of statins are well documented in many patients and are a main reason for non-compliance. CETP inhibitors, especially evacetrapib and obicetrapib, are well-tolerated and have almost no reported off-target effects. **3)** While both, CETP and HMGCR, are druggable, CETP is much easier accessible since it is mainly active in the blood stream.

Regarding the amyloid biomarker: In humans, plasma amyloid did not change with CETP inhibition, only when data were stratified for APOE4 carriers. It is therefore not surprising that we saw no change in amyloid in our current APPxCETP mouse model, since it lacks the representation of human APOE isoforms. We are currently developing the APOE3/4xCETP mouse model to investigate CETP inhibition in an APOE-dependant manner.

A – plasma p-tau217

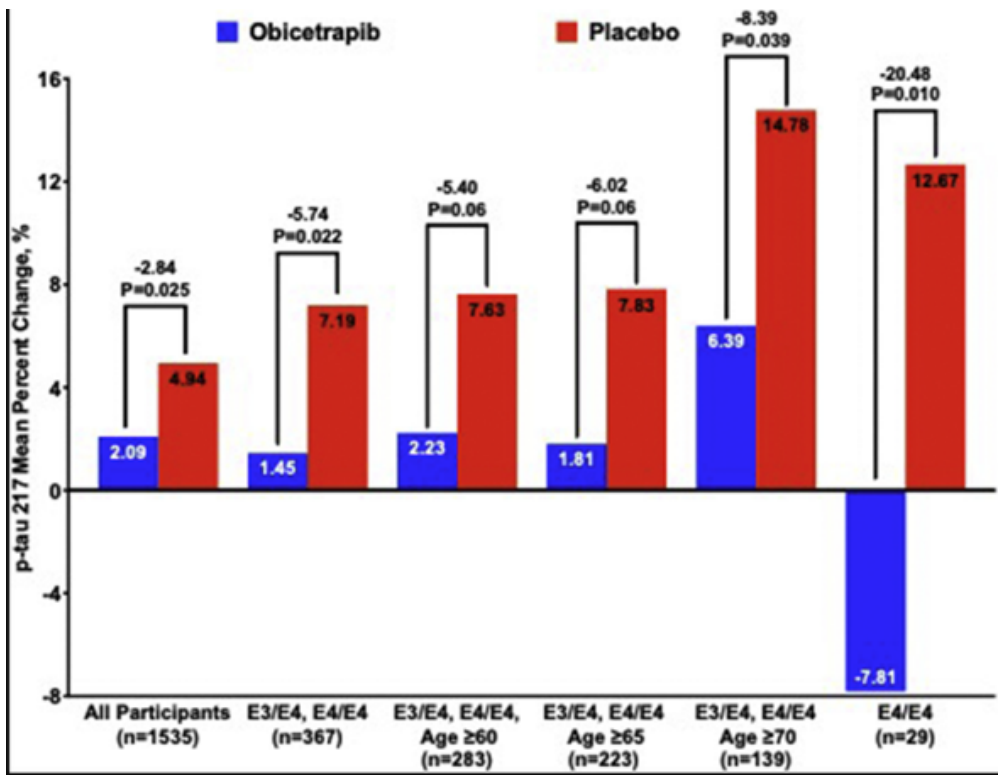

B – plasma biomarkers in APOE4/4 carriers

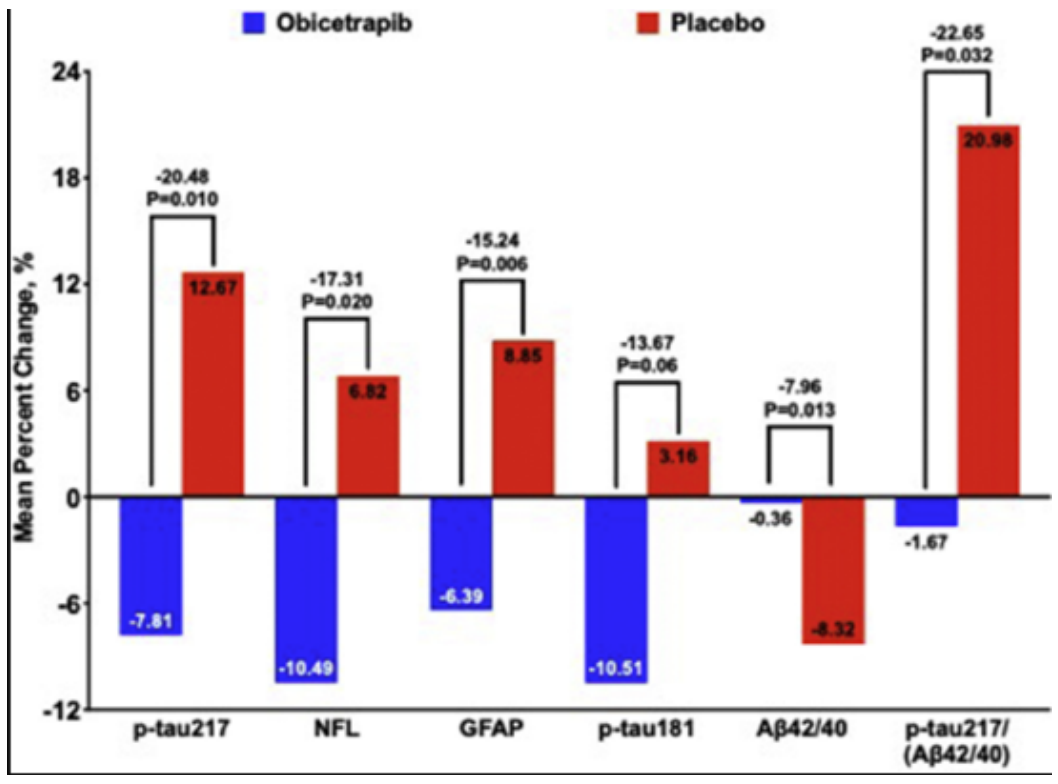

Images taken from Davidson et al., *J Prev Alzheimers Dis* 2026; PMID: 41109840, (1).

Regarding peripheral lipid changes: There are other reports supporting the notion that peripheral lipid regulation impacts cognitive performance. A strong example is a publication by Gujon Bu, a leader in the Alzheimer's field on research for the apolipoprotein E (APOE), who recently showed that peripheral expression of APOE4 impacts cognitive performance through affecting the cerebrovascular function in line with results from our manuscript (Liu et al., *Nature Neuroscience*, 2022, PMID: 35915180 (4)).

Further, please see the confidential data presented below to the first response to reviewer 1: We show unpublished data that CETP expression *only* in the liver is sufficient to induce cognitive impairment, which demonstrates that peripheral CETP can be a disease-modifying drug target.

Edits to the manuscript to address this concern:

We included the new biomarker study by New Amsterdam Pharma in the introduction, which now reads: " A recent pre-specified clinical substudy of the BROADWAY trial showed that CETP inhibitor obicetrapib significantly slowed AD biomarker progression with the most pronounced impact on p-tau217 in APOE4 carriers (*30*)."

We edited the discussion section to include the clinical study, which reads now: "Our findings are supported by the BROADWAY study, a recent AD plasma biomarker study with CETP inhibitor obicetrapib showing that plasma Aβ levels did not change, except in APOE4 carriers (*30*). As our mouse model does not recapitulate the different APOE isoforms, this may explain why we did not observe changes in Aβ levels. Future studies will have to address the interaction of CETP with APOE isoforms in mouse models. Whether CETP inhibition in the periphery, the brain, or both contributed to memory preservation remains to be determined as well (*38, 42, 78*). Our results together with the clinical AD biomarker BROADWAY study of CETP inhibition (*30*) ….".

And further in the discussion: "The above mentioned recent clinical data are the first trial results demonstrating a delay in AD plasma biomarker progression, especially in APOE4 carriers, providing first human evidence of the benefits of CETP inhibition in AD (*30*). Given that CETP is mainly active only in plasma…"

Referee #1 (Remarks for Author):

In this manuscript under consideration, Phenix et al explore the role of cholesteryl ester transfer protein (CETP) in Alzheimer's disease (AD) and in an AD mouse model based on epidemiological studies showing correlations between reduced CETP activity/levels and reduced AD risk. CETP is

best characterized in the field of atherosclerosis and has been shown to transfer CE from HDL to LDL, leading to development of small molecule CETP inhibitors (like evacetrapib, which is used in this study). These inhibitors have been largely deprioritized mostly based on evidence they are not superior to statins. However, CETP and their inhibitors have not been investigated in the context of AD models. Because CETP is not expressed in mice, its study requires the use of transgenic models expressing human CETP, which were previously generated in the field. The authors therefore crossed these CETP transgenic mice to an APP mouse model and assessed the impact of evacetrapib on AD relevant endpoints. Their study provides evidence that CETP inhibition confers cognitive benefits based on behavioral studies, while they have no effect on amyloid beta markers assessed biochemically. The authors show some brain cholesterol redistribution based on filipin stains (which label free cholesterol) and neutral lipid stains, possibly assessing CE levels. evacetrapib treatment in CETP transgenic mice caused transcriptomic changes in BBB-related genes. Finally, human CSF proteomic datasets from PREVENT-AD were generally in line with preclinical data. Overall the study makes a case that CETP inhibitors should be considered for AD.
This is an interesting study highlighting a possible role of CETP in AD and a potential clinical use of CETP inhibitors in AD. While the study shows potential benefits of evacetrapib in an AD mouse model, there are still some fundamental questions that need to be addressed for a greater impact.

We thank this reviewer for the overall positive assessment and interest of our work.

Main comments:
1. While the authors do a good job assessing the impact of CETP expression and inhibition in mice in the periphery, it is very much unclear whether target engagement in the CNS is required for the beneficial effects of the drug. This is particularly important because the drug used is claimed to be CNS-penetrant. Generally, unbound drug levels are to be measured in the blood and in the brain to understand whether the drug is actually brain-penetrant. At the very least, there should be an attempt to assess target engagement in the CNS. Given the role of CETP in CE metabolism, it seems surprising that this metabolite is not included in the sterol characterization, which is otherwise very thorough. The authors should therefore assess CE levels in the brain of control and transgenic mice expressing CETP in the presence of absence of mutant APP expression and assess also the impact of evacetrapib. This could be done in the mouse CSF as well, although it may be more technically challenging.

We thank the reviewer for this comment and agree with it.

A) **Addressing the critique regarding peripheral or central CETP inhibition**: We have invested considerable work prior to the revision to test for target engagement in the brain, aiming to dissect peripheral from central CETP inhibition. Throughout our attempts, we faced experimental difficulties, which I will elaborate below. However, we can address this concern with a different approach: We recently expressed CETP only in the liver of wild type mice using an adeno-associated virus (AAV) encoding for CETP under the liver-specific TBG promoter (initially developed by the lab of Dr. Rader, Tanigawa et al., *Circulation*, 2007 (11)). This way, we can ensure that CETP is only expressed in the periphery. We measured cognitive performance in these mice at the same age as in the present manuscript, and found peripheral CETP-expressing mice cognitively impaired as well (see figure below). These data are confidential and will be published in a separate manuscript. We conclude that peripheral CETP expression is sufficient to induce cognitive impairment. Consequently, target engagement in the periphery – which has been widely demonstrated, also shown in this manuscript - will impact cognitive

performance, too. Thus, target engagement in the brain may happen, but may not be needed to improve cognition.

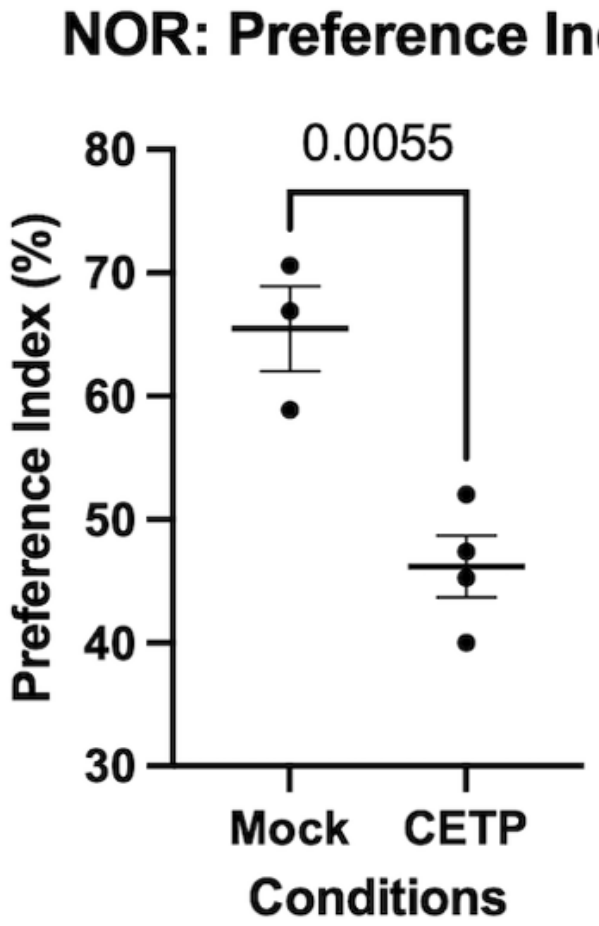

Expression of CETP in wild type mice only in the liver using AAV-TBG-CETP vectors compared to AAV-TBG-mock. At 5.5 months of age, mock mice perform well at 65% as observed previously, while CETP-expressing mice show cognitive impairment comparable to that of CETP-transgenic mice used in the present manuscript. Confidential data.

In the following, we list experiments that we conducted to dissect peripheral from central effects, and their experimental issues:

1) From the trial mice of this manuscript, we collected brain hemispheres and liver samples for quantification of evacetrapib by mass spectrometry as we have done previously (Phénix and Coté et al., Front Pharmacol, 2023; PMID: 41109840). *Please note that the latter paper is also the one in which we demonstrated that evacetrapib crosses into the brain, in collaboration with Dr. Michèle Bouchard.* While we could detect evacetrapib in liver tissues, the concentration was below the detection limit for the brain samples. Noteworthy, the trial mice of the present manuscript were injected i.p., while the published pharmacokinetic study used i.v. injections. We conclude that the route of administration prevented the drug to peak in the brain, and the repeated dosing did not lead to drug accumulation in the brain.
2) To date, we were not able to detect CETP protein (western blot, IP, or IHC) or CETP activity from brain samples, while we could detect CETP mRNA {Oestereich, 2022 #41}. CETP is a very hydrophobic protein, which will not easily extract from lipid-rich brain tissue. Being lipid bound may also mask antibody epitopes. Commercial antibodies are good, but not excellent. CETP is a low-abundant protein in the brain, contributing to the detection limit of our available assays.
3) An important consideration in the dissection of peripheral and central effects of CETP inhibition is, that a recent study by Tony Whyss-Coray showed that over 1400 proteins present in the brain have no transcripts, suggesting that way more proteins cross the blood-brain barrier than previously assumed (Yang et al., Nature 2020; PMID: 32612231 (12)). Since the study was conducted in wild type mice, CETP was not part of that protein list, and it is unknown if CETP may cross the blood-brain-barrier. The apolipoprotein A1 (APOA1) is one of these proteins efficiently crossing from blood to the brain (Stukas et al., J Am Heart Assoc., 2014 (13)). CETP likely associates with APOA1 and nascent HDL particles, so that CETP could potentially cross into the brain as a complex, but it is currently unknown. Therefore, any study the aims to show target engagement in the brain will need to consider this potential dynamic transport. However, in light of our new, confidential data that peripheral CETP expression alone is sufficient to cause cognitive impairment, this knowledge may not even be needed. We addressed this issue in the

conclusion section where we now state: “Furthermore, it is currently unclear if CETP crosses the blood brain barrier.”

B) **Regarding cholesteryl esters (CE) in the brain:** For the very same reasons outlined by the reviewer, we aimed to quantify CEs from brain tissue using MALDI-imaging mass spectrometry (data not in the manuscript) as well as ESI-MS (data included). However, the concentration of CEs is quite low in the brain and both sensitive mass spectrometry techniques did not allow to detect CEs from either brain sections or bulk lipid extracts. Further, mouse CSF collection is technically challenging and yields very limited volumes, typically ≤10 µL per animal, which poses significant challenges for lipidomics, particularly for low-abundance species such as CEs.

2. The authors do a good job assessing soluble and insoluble Abeta levels in the mouse brain, and based on these analyses, it does not appear that CETP expression or inhibition affects these levels. However, for a study of this scope, it is also important to examine plaque counts and morphology and include assessments of neuronal damage. Specifically, does CETP expression or inhibition affect amyloid plaque size distribution, morphology (e.g. compaction) and neuritic dystrophy?

We thank the reviewer for this comment. We indeed stained brain sections for plaques using thioflavin T or the 6E10 antibody, however, we could not detect any plaques, which is likely due to the early age of the mice (5.5 months) at which we determined cognitive performance and then sacrificed the mice. As an alternative to plaque detection by immunohistochemistry, so-called “insoluble Abeta” can be extracted using formic acid. Formic acid will solubilize Abeta even from beta-sheet aggregates (pre-plaques or plaques). Abeta peptides can then be quantified using ELISA, which we showed in Figures 3F, 3H for Abeta40 and Abeta42, respectively. The data show that, overall, much less insoluble Abeta is detected as compared to soluble Abeta, consistent with the immunohistochemistry results that showed no Abeta plaques. We detected no differences between genotypes or treatments for insoluble Abeta.

It should further be noted that it is well-established that plaques do not correlate with cognitive performance, and ideally, interference with the disease process happens well before plaques are formed. Please also consider our first response above regarding the human trial data using the CETP inhibitor obicetrapib. The amyloid biomarker in blood decreased only in APOE4 carriers with obicetrapib. Since our mouse model of the present manuscript does not allow us to compare APOE3 to APOE4, the current model may not be suitable for the amyloid marker, which is why we are currently establishing an APOE-APP-CETP model. We address this point explicitly now in the discussion (see this citation above on the BROADWAY trial as well): “As our mouse model does not recapitulate the different APOE isoforms, this may explain why we did not observe changes in Aβ levels. Future studies will have to address the interaction of CETP with APOE isoforms in mouse models.”

3. Related to the previous point, how is CETP inhibition beneficial in the APP mouse model if plaques are unaffected? Is synaptic loss rescued? Are microglia or inflammatory responses affected in any ways? Are the neutral lipid stains reduced by CETP inhibition restricted to microglia?

We tested many different hypotheses deduced from the literature on how CETP inhibition may benefit memory, and found no supporting evidence for any (for example the hypothesis that prenylation is affected; answered in Supplemental Figure 4). We therefore turned to unbiased methodology and conducted bulk RNA-sequencing, which revealed that CETP inhibition primarily changed the transcriptome of ***endothelial cells*** in the brain (Figure 5). We did not anticipate this observation at all, however, reflecting on these results now, it makes much sense that a peripheral lipid-shuttling protein affects the health of endothelial cells. To emphasize this conclusion of our work and to address this question, we have now added new data to the manuscript showing the PECAM1/CD31 staining by immunohistochemistry in brain sections of our mice **(Figure 5G)**. Indeed, consistent with the increase of PECAM1 transcripts identified by RNA-sequencing through CETP inhibition, we find that evacetrapib significantly increased PECAM1 staining intensity in APPtg/CETPtg mice as compared to vehicle. We then developed an *in vitro* assay, in which we cultivated mouse endothelial cells activated by TNF-α, and treated them with plasma from either wildtype or CETPtg mice, now added as a new figure to the manuscript (**Figure 5H**). Here, we found that plasma from CETPtg mice downregulated PECAM1 expression by approx. 22% after 2 h only, consistent with PECAM1 upregulation when CETP is inhibited. This *in vitro* assay confirms that CETP activity impacts PECAM1 expression, which is a marker of endothelial health through several mechanisms (14). We find that this new data strongly supports our conclusions. Future research will assess the length and branching of the vessels as well as their functionality, as this level of assessment is beyond the scope of the present manuscript.

Further, we stained brain sections for GFAP as a marker of astrocytes, but found no obvious differences between genotypes and treatments (data not shown).
We also analyzed brain lysates by SDS-PAGE and western blot for PSD-95 levels, but again, with this method, we found no differences between genotypes and treatments (data not shown). Similarly, we could not yield cellular resolution of the neutral lipid stain.

In conclusion, our data suggest that cognitive maintenance through evacetrapib was achieved by maintaining vascular health and possibly proper perfusion, which we will investigate in further projects. We edited the manuscript results section accordingly, which now reads:
"Considering that endothelial cells make only about 0.2-7 % of all cerebral cells (*70*), the detection of endothelial-specific genes in this bulk analysis was unexpected. To substantiate this, we quantified PECAM1 protein levels using immunohistochemistry (Fig. 5G). Indeed, double transgenic APPtg/CETPtg mice on evacetrapib exhibited increased PECAM1 signal intensity as compared to the vehicle group (Fig. 5G). To validate the regulation of PECAM1 by CETP activity *in vitro*, mouse endothelial bEND.3 cells were activated with tumor necrosis factor-a (TNF-a) in the absence of lipoproteins. Then plasma from WT or CETPtg mice was added for 2 h and PECAM1 detected by western blot. Plasma from CETPtg mice downregulated PECAM1 compared to WT plasma (Fig. 5H). This finding is consistent with the in-vivo observation that PECAM1 increased following CETP inhibition in mouse brains. Given that PECAM1 is a well-established marker of endothelial health (*71*), these results suggest CETP inhibition maintains endothelial health, which may be a key mechanism underlying the memory benefits of CETP inhibition."

We also edited the abstract: "Using CETP transgenic mice crossed to a mouse model of amyloidosis and administering evacetrapib, we provide evidence that CETP inhibition maintained memory independent of classic AD markers*, likely through maintained vascular health, while increasing* hippocampal cholesterol, *and altering* plasma lipoproteins."

Minor comments:
4. Growing evidence suggests brain cholesteryl esters are linked to AD and AD models (both amyloid and tau); this is not discussed in the manuscript and perhaps a missed opportunity to build a case for the potential significance of the CETP pathway. Perhaps the authors should do a more thorough job discussing this evidence in the introduction and discussion sections.

We thank the reviewer for this insightful suggestion. We agree and have now expanded the Discussion to summarize evidence that brain cholesteryl esters are increased in AD brains. The respective section now reads: "Since we did not focus our analyses on cholesteryl esters, further investigation is required to establish if CETP activity alters the cholesteryl ester content or quality in the brain and/or plasma. Other studies observed that cholesteryl esters change during early AD stages and track with the progression of the disease, potentially serving as plasma biomarkers (*53, 120, 121*)."

5. While the physiological function of CETP is well established in peripheral lipid metabolism, it is unclear, at least to this reviewer, what CETP might do in the CSF/ISF. As the authors are likely aware of, there are HDL-like particles in the CSF and their size is generally larger than peripheral HDL lipoproteins. What CE transfer reactions do the authors hypothesize might be mediated by CETP? They should comment on this or at the very describe the unknown as a key question.

We agree with this comment, it is indeed important to discuss that aspect. Again, as elaborated above, CETP does not need to be expressed in the brain or cross the blood-brain-barrier to elicit the positive effects on the endothelial cells when inhibited. Our attempts to detect CETP in the brain at the protein or activity level did not yield positive results. CETP was clearly detected in the CSF of the PREVENT-AD patient cohort, suggesting that CETP can cross the blood-brain barrier or is expressed in the brain. One publication suggested that CETP can extract lipids directly from the plasma membrane (19), so it could be a possibility that CETP exchanges lipids between plasma membranes and HDL-like particles. However, this is speculative at this point, so that we decided to add this point of discussion in the "limitations" section, which now reads:

"While brain and CSF lipids are primarily transported by HDL-like particles, the exact role of CETP in brain lipid transfer remains unclear."

Referee #2 (Comments on Novelty/Model System for Author):

My notes on statistics are noted in my review. This should be addressed and/or clarified but not a major concern.

Referee #2 (Remarks for Author):

This study by Phénix explores the preclinical use of the CETP inhibitor evacetrapib on Alzheimer's disease mouse model. Since mice don't express CEPT, they crossed a CEPT expressing mouse with APP mouse model of amyloidogenesis. They discovered that evacetrapib effectively altered plasma lipoprotein profile in favour of the protective HDL particles. This drug also improved memory in females. They conclude this is due to a redistribution of lipids in the brain without any leakage of the blood-brain barrier, Finally, they performed a proteomic analysis and confirmed that their model system recapitulates early in the disease progression, and that many alterations in

proteins converge on the vasculature.

I found this study very interesting. The rationale outlining the reasons why CEPT is an ideal target for Alzheimer's disease therapy is extremely compelling. And their data supports this as a promising target for therapy in early stages of the disease to prevent or limit its progression. Overall, I am quite enthusiastic about the study. Below I have outlined some points to clarify before the paper would be ready for publication:

We thank this reviewer for their enthusiasm and positive evaluation of the manuscript.

1. Is expression of CETP in the CETPtg line sufficient to increase risk of cognitive decline in aged animals? Perhaps previous studies have looked at this? If so, it would be useful information to include when the introducing the animal lines.

We thank this reviewer for their comment, we agree. In fact, it has *not* been previously assessed if CETP expression in mice affects cognitive performance. Our data in Figure 3B, 3C are the *first to report* on cognitive performance of CETPtg mice. CETPtg mice at 21 weeks of age show cognitive impairment in the novel object recognition test, while administration of evacetrapib rescued cognitive performance.

The cognitive results of CETPtg mice are well described in the results section, we find. However, to emphasize the novelty of our data, we added “CETPtg” to the subsection heading as well as added a subordinate clause in the results section: “which was not reported to our knowledge thus far”.

2. It would be helpful to mention that in the CETPtg, that CETP is under the expression of its endogenous promoter. This is important given the role peripheral lipoproteins can have on cognition. This information could prevent confusion as to what can and cannot be concluded from the experiments.

We agree and thank for this comment. The information was provided in the introduction, which we edited to emphasize this point. It now reads: “To address this challenge, a mouse strain expressing the human *CETP* gene under its own *human endogenous* promoter was previously generated…”

3. It would be helpful to better describe the relevance of the 1% cholesterol diet. How much cholesterol is in the regular chow? Is 1% associated with any notable health outcomes in the mice?

We appreciate this reviewer comment. However, the choice of diet is not a central research question of this manuscript, and was assessed by our lab and others previously. The citation from our lab is Oestereich et al., JLR 2022, cited in the manuscript text when introducing the cholesterol diet. In Oestereich et al., we compared a standard chow (standard chow contains very little cholesterol), to 1% (w/w) cholesterol diet, and to 1% cholesterol plus high fat diet in CETPtg mice. As elaborated in Oestereich et al., we found CETP expression to be higher with 1% cholesterol diet as compared to the cholesterol plus fat diet. Additionally, mice gained weight with the cholesterol plus fat diet, which was absent in the 1% cholesterol chow only. Thus, we chose 1% cholesterol to avoid confounding effects such as obesity and to only express CETP. We tested a 0.4% cholesterol diet which did not fully induce CETP expression (unpublished). The cholesterol

diet leads to lipid accumulation in the liver, which we consider acceptable since the duration is limited, based on our unpublished observations. Since all mice of the study received the same diet, and wild type mice performed well in the cognitive tests, we are not concerned that the diet induced any form of toxicity. We would not give the 1% cholesterol diet for more than 12 weeks (while there are published studies giving the diet for 1 year). We added to the text an additional explanation as to why the diet is necessary referring to the sterol responsive elements which now reads: "via nuclear LXR receptors elements" and added citation Luo et al., JCI, 2000.

4. I find the description of Figure 2 confusing. In figure 2, the authors claim that CEPT expression increased LDL and decreased HDL in vehicle treated CEPT-expressing mice compared to control. But go on to clarify that is only in the female mice. But it doesn't seem like this comparison was made using statistics as they only show the comparisons for vehicle vs evacetrapib. Looking at the graphs, I do not see a difference between plasma HDL in control vs CETP mice in the females. (The correlation graphs in supplemental Fig 1 look good but answer a slightly different question.)

We thank the reviewer for this comment and catching our oversight. It is correct that the statistics as presented did not allow any conclusion on lipoprotein particles between WT and CETPtg mice on vehicle. We observed indeed mostly trends. To support the lipoprotein remodelling effect as a function of sex, we now included a three-way ANOVA test. The results section now reads: "…CETP activity decreased HDL and increased LDL levels by trend in vehicle-treated CETP-expressing mice as compared to WT or APPtg mice (Fig. 2A-F). Using a three-way Anova test on the combined data, we found that sex is a major driver of HDL and LDL differences, in addition to genotype and treatment (Fig. 2A-D). Accordingly, CETP inhibition in female CETPtg mice showed statistically significant changes in HDL and LDL levels as compared to their respective vehicle controls (Fig. 2A, B)."

And Figure 2 Figure legends now reads: "(**A-D**) Plasma lipoproteins HDL and LDL were quantified. Data are shown stratified by sex (*n=6-8)*. Statistical inference was based on a three-way ANOVA including sex, genotype, and treatment, followed by Tukey's post-hoc test (genotype HDL $p=0.05$, LDL $p<0.001$; sex HDL/LDL $p<0.001$; treatment HDL/LDL $p<0.001$). (**E, F**) Data from A/C or B/D shown and with sexes combined for visualization only *(n=12-14);* two-way ANOVA followed by Tukey's post-hoc test. Scatter dot plots with indicated mean ± SEM with each point depicting a sample from one mouse."

5. I have the same issue with Figure 3. I can see the effect, that the APP, CETP and APP-CETP mice don't discriminate as well as the WT. But I only see the comparisons being made between the vehicle and evacetrapib in the females, which indeed is convincing.

We agree, the reviewer is correct that we did not indicate the significances between vehicle groups. We now added "*" symbols in Figure 3B and report the p values between WT vehicle, and the respective genotype-vehicle in the figure legend (2-way ANOVA). The Figure legend 3 now reads: "(**B**) NOR scores combined for both sexes *(n=12-14)*. Red line indicates the 50% discrimination ratio at which mice cannot differentiate between the familiar and the novel object suggesting memory impairment. Two-way ANOVA followed by Tukey's post-hoc test. *depict significant differences to the WT vehicle group (for APPtg-V $p=0.03$; CETPtg-V $p=0.035$; APPtg/CETPtg-V $p=0.025$). (**C-D**) NOR scores for each sex for visualization. Two-way ANOVA followed by Tukey's post-hoc test."

We further added in the results section a 2-way ANOVA test that allowed us to conclude that the cognitive impairment of APPtg/CETPtg double transgenic mice is indeed based on a superadditive effect. The results section now reads: "The double transgenic APPtg/CETPtg mice exhibited memory impairment as well, due to a superadditive effect of both transgenes (p=0.04, two-way Anova test)."

6. I find it curious that evacetrapib shows a slight but nonsignificant decrease in soluble Abeta given that CEPT is not expressed (Fig3E and G). Could this be due to a slightly under powered analysis? Could evacetrapib have off-target effects. Seeing this does not diminish my enthusiasm for the study, as the results are very promising from a preclinical standpoint. But perhaps something to consider from a fundamental perspective.

We understand the reviewer's point. We looked at the statistics of Abeta levels from both sexes combined and the levels appear even with no differences or trends. We conclude that this apparent effect of evacetrapib in APPtg females is not a true effect. The first generation CETP inhibitor torcetrapib had reported off-target effects in humans and mouse models lacking CETP. However, these off-target effects have *not* been reported for evacetrapib (21). It should be noted that thousands of subjects in the ACCELERATE trial took evacetrapib over two years which was overall well tolerated (22). We are planning on repeating a similar mouse trial with a different APP transgene, and we will monitor Abeta levels again, which may reveal more insight into potential off-target effects.

7. Fig 4. Why do you think you see more hippocampal cholesterol with filipin staining but not in the whole brain? Could this be validated by dissecting out the hippocampus and looking at cholesterol levels biochemically?

Cholesterol is not evenly distributed in the brain as shown by others (23) and our lab (5, 24). The figure below is taken from Nezhad et al., *Anal Bioanal Chem* 2022, showing MALDI-imaging mass spectrometry data with the spatial distribution of cholesterol in the brain as a heatmap. The corpus callosum is especially rich in cholesterol. Depending on how accurately the brains are dissected, slight differences in the amount of corpus callosum may average out any cholesterol differences after biochemical lipid extraction and ESI-MS quantification. We tried to do MALDI-imaging MS with brain sections from our cohort, however, only 5 brains can be images in parallel increasing batch-to-batch variation between our 8-group cohort, and additionally, the brain sections did not show comparable regions and therefore complicated quantification. With the filipin staining, we gained great quality results which were quantifiable with spatial resolution. However, filipin quenches quickly, so that we only imaged the hippocampus in greater depth.
To account for these clarifications in the manuscript text, we edited the results section, which now reads: "Thus, while we detected no lipid changes with bulk GC-MS analysis*, which was most likely due to the naturally uneven distribution of cholesterol in the brain (42) and variability during brain dissections,* the spatial information gained by analyzing brain sections revealed that evacetrapib leads to a significant redistribution of cholesterol and a remodelling of lipid stores, *at least* in the hippocampus, providing an additional *potential* explanation for the maintenance of memory by CETP inhibition."

To dissect the hippocampus, we would need to breed and age a colony again to gain fresh material, as all collected materials from this trial go to an end. We hope that the reviewer understands that this is tremendous effort which we did not prioritize for this revision.

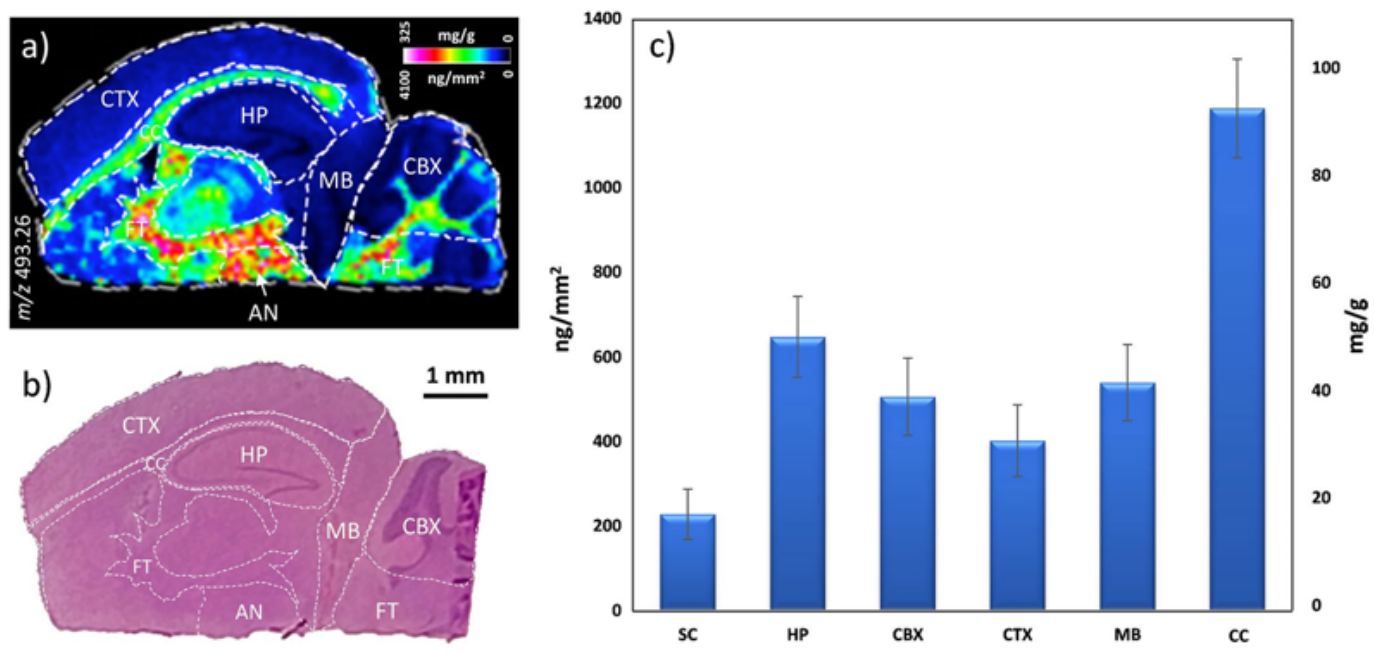

Fig. 4 Cholesterol absolute quantification within the mouse brain and specific brain substructures. (**a**) Sagittal MALDI MSI ion image for cholesterol (*m/z* 493.26), and (**b**) matching H&E staining from a serial section. (**c**) Cholesterol amounts within the sections are provided in areal density (ng/mm^2) and weight per weight of wet brain tissue (mg/g). Average values were derived from the six sagittal sections mounted on slides 1, 2, and 3. The error bars represent the standard deviation. Detailed values can be found in Electronic Supplementary Material Table S2. SC, sagittal cut; Hp, hippocampus; CBX, cerebellum; CTX, iso cortex; MB, mid brain; CC, corpus callosum; FT, fiber tracts; AN, amygdalar nucleus

8. In figure S8 the changes in neutral lipids (or lack thereof) seems underpowered. It seems like evacetrapib does in fact lower neutral lipids in S8A.

We agree with this concern. We ran the statistical testing again on the quantification data using the GraphPad Prism software, and we found significant neutral lipid differences in APPtg/CETPtg mice on vehicle versus evacetrapib. Likewise, the ratios of cholesterol to neutral lipids gained significance in CETPtg and APPtg/CETPtg mice between vehicle and evacetrapib treatment. We are unclear why we did not indicate such differences in the initial submission, so we thank the reviewer for catching our oversight. Also, it should be noted that one data point in the figure represents the average staining intensity from one animal derived from several brain sections.

Change: New Figure S8 with statistical significances in Panels A and C.

9. In Figure S8, the authors look at the intensity of BODIPY665/676 to analyze neutral lipids. This lipid dye difficult to use in brain tissue because it can often stain other lipid species non-specifically. It also doesn't distinguish between TAGs and CEs. The authors mentioned they don't detect any changes with bulk GC-MS. I think it would strengthen the manuscript to include the omics data for TAGs and CEs separately.

We agree with this reviewer's request in principle, however, we are limited by the methodology. The total amount of cholesteryl esters in the brain is small, and we did not detect it by our GC-MS analysis. It is likely that BODIPY detected mostly TAGs. However, TAGs are large and not well ionizable, limiting their detection by GC-MS, too. Cholesteryl esters and TAG can be measured by mass spectrometry, which would require some extra steps and derivatization during the lipid extraction and sample preparation procedure. Since we are limited in the amount of brain material coming from these aged mice, we focused on answering the hypotheses on cholesterol synthesis and excretion, and not on neutral lipids. So as much as we would like to have such data, we were limited by sample amount and methodology, and hope the reviewer can appreciate the demonstration of the spatial neutral lipid data.

10. I find it a very surprising that you have alterations in genes converging on the blood brain barrier and yet no leakiness. Especially given the defects in the BBB in later stages of Alzheimer's disease. It could be interesting to discuss this direction a little more.

We appreciate this comment. We sacrificed the mice at a very early stage, when BBB leakiness had not yet occurred. However, we detected early endothelial dysfunction by RNA-sequencing which likely occurs prior to leakiness, supported by PECAM-1 levels in brain sections and in cell

culture models (new data added in Fig. 5G, H during revisions). We discuss this point now in the discussion section which reads: “Leakiness of the blood-brain-barrier is another established pathology in AD. However, its absence in our cohort of relatively young mice suggests that we are observing an earlier stage of pathology, whereby more detailed analyses will be needed. Rather, our findings suggest that endothelial dysfunction, which we detect here, precedes overt blood-brain-barrier breakdown. For example, PECAM-1 levels serve as an indicator of endothelial integrity through several pathways (*71*).” And “Further projects will investigate the functionality of endothelial cells and angiogenesis with CETP inhibition.”

Comments by the Authors:

As a result of the revision process, we have 4 additional authors, i.e., Antonio Vazquez Coba, a senior graduate student in the lab, who conducted the PECAM1/CD31 staining and quantifications in collaboration with Clara Berger, visiting student. Indie Jerry, undergraduate student in pharmacology, conducted the in vitro assay for PECAM1/CD31. Aaron Marure, Master’s student in the lab, supported the revision of statistical analyses of the data in the revised version.

We also edited the Acknowledgements by adding: *“We thank Gege Mei and Rose Rodrigues for guidance with immunohistochemistry experiments.”*

We clarified the description for Figure 6 regarding the p-tau181/Ab42 ratio in the results section, which now reads: “However, we observed a significant effect of the CSF p-tau(181)/Aβ42 ratio on VCIS suggesting that this ratio is a strong predictor of worsening cognition and indicates conversion to MCI and potentially AD in these individuals (Fig. 6B). Taking the relationship of the p-tau(181)/Aβ42 ratio over cognition, and separating the top 50% CETP levels from the bottom 50%, the data revealed that low CETP levels were significantly associated with better cognitive performance (VCIS) like in our mouse model (*73*) (Fig. 6C).”

We further found two labelling mistakes in Figure 5 and Supplemental Figure 4, which we corrected and which do not impact the results and conclusions. In Figure 5B, the genotypes were annotated inversed, i.e., dark violet on the left side of the heatmap is from double transgenic APPtg/CETPtg mice and light violet on the right side of the heatmap is from CETPtg mice. In Supplemental Figure 4, we created a new Western blot figure since there was a discrepancy between the labelling of the raw data and the labelling in the Figure, which is now corrected.

Figure 6A was labelled on the x-axis as “Ab42 ratio” which we corrected to “Ab42”.

Supplemental Figure 10A was labelled on the x-axis as “Ab42 ratio” which we corrected to “Ab42”.

1. Davidson, M. H., Szarek, M., Scheltens, P., Vijverberg, E., Hsieh, A., Ditmarsch, M. *et al.* (2026) Effect of obicetrapib, a potent cholesteryl ester transfer protein inhibitor, on p-tau217 levels in patients with cardiovascular disease J Prev Alzheimers Dis **13**, 100394 10.1016/j.tjpad.2025.100394

2. Yamashita, S., Hui, D. Y., Sprecher, D. L., Matsuzawa, Y., Sakai, N., Tarui, S. *et al.* (1990) Total deficiency of plasma cholesteryl ester transfer protein in subjects homozygous and heterozygous for the intron 14 splicing defect Biochem Biophys Res Commun **170**, 1346-1351 10.1016/0006-291x(90)90542-u
3. Yokoyama, S. (2015) Unique features of high-density lipoproteins in the Japanese: in population and in genetic factors Nutrients **7**, 2359-2381 10.3390/nu7042359
4. Liu, C. C., Zhao, J., Fu, Y., Inoue, Y., Ren, Y., Chen, Y. *et al.* (2022) Peripheral apoE4 enhances Alzheimer's pathology and impairs cognition by compromising cerebrovascular function Nat Neurosci **25**, 1020-1033 10.1038/s41593-022-01127-0
5. Oestereich, F., Yousefpour, N., Yang, E., Phenix, J., Nezhad, Z. S., Nitu, A. *et al.* (2022) The cholesteryl ester transfer protein (CETP) raises cholesterol levels in the brain J Lipid Res **63**, 100260 10.1016/j.jlr.2022.100260
6. Jiang, X. C., Agellon, L. B., Walsh, A., Breslow, J. L., andTall, A. (1992) Dietary cholesterol increases transcription of the human cholesteryl ester transfer protein gene in transgenic mice. Dependence on natural flanking sequences J Clin Invest **90**, 1290-1295 10.1172/JCI115993
7. Bjorkhem, I., andMeaney, S. (2004) Brain cholesterol: long secret life behind a barrier Arterioscler Thromb Vasc Biol **24**, 806-815 10.1161/01.ATV.0000120374.59826.1b
8. Tall, A. R. (1993) Plasma cholesteryl ester transfer protein J Lipid Res **34**, 1255-1274, https://www.ncbi.nlm.nih.gov/pubmed/8409761
9. Barter, P. J., Brewer, H. B., Jr., Chapman, M. J., Hennekens, C. H., Rader, D. J., andTall, A. R. (2003) Cholesteryl ester transfer protein: a novel target for raising HDL and inhibiting atherosclerosis Arterioscler Thromb Vasc Biol **23**, 160-167 10.1161/01.atv.0000054658.91146.64
10. Barter, P. J., Hopkins, G. J., andCalvert, G. D. (1982) Transfers and exchanges of esterified cholesterol between plasma lipoproteins Biochem J **208**, 1-7 10.1042/bj2080001
11. Tanigawa, H., Billheimer, J. T., Tohyama, J., Zhang, Y., Rothblat, G., andRader, D. J. (2007) Expression of cholesteryl ester transfer protein in mice promotes macrophage reverse cholesterol transport Circulation **116**, 1267-1273 10.1161/CIRCULATIONAHA.107.704254
12. Yang, A. C., Stevens, M. Y., Chen, M. B., Lee, D. P., Stahli, D., Gate, D. *et al.* (2020) Physiological blood-brain transport is impaired with age by a shift in transcytosis Nature **583**, 425-430 10.1038/s41586-020-2453-z
13. Stukas, S., Robert, J., Lee, M., Kulic, I., Carr, M., Tourigny, K. *et al.* (2014) Intravenously injected human apolipoprotein A-I rapidly enters the central nervous system via the choroid plexus J Am Heart Assoc **3**, e001156 10.1161/JAHA.114.001156
14. Privratsky, J. R., andNewman, P. J. (2014) PECAM-1: regulator of endothelial junctional integrity Cell Tissue Res **355**, 607-619 10.1007/s00441-013-1779-3
15. Kimble, A. L., Silva, J., Omar, O. M., Murphy, M., Hensel, J. A., Nicholas, S. E. *et al.* (2022) A method for rapid flow-cytometric isolation of endothelial nuclei and RNA from archived frozen brain tissue Lab Invest **102**, 204-211 10.1038/s41374-021-00698-z
16. Cunnane, S. C., Schneider, J. A., Tangney, C., Tremblay-Mercier, J., Fortier, M., Bennett, D. A. *et al.* (2012) Plasma and brain fatty acid profiles in mild cognitive impairment and Alzheimer's disease J Alzheimers Dis **29**, 691-697 10.3233/JAD-2012-110629

17. Tajima, Y., Ishikawa, M., Maekawa, K., Murayama, M., Senoo, Y., Nishimaki-Mogami, T. *et al.* (2013) Lipidomic analysis of brain tissues and plasma in a mouse model expressing mutated human amyloid precursor protein/tau for Alzheimer's disease Lipids Health Dis **12**, 68 10.1186/1476-511X-12-68
18. Proitsi, P., Kim, M., Whiley, L., Pritchard, M., Leung, R., Soininen, H. *et al.* (2015) Plasma lipidomics analysis finds long chain cholesteryl esters to be associated with Alzheimer's disease Transl Psychiatry **5**, e494 10.1038/tp.2014.127
19. Wanschel, A., Guizoni, D. M., Lorza-Gil, E., Salerno, A. G., Paiva, A. A., Dorighello, G. G. *et al.* (2021) The Presence of Cholesteryl Ester Transfer Protein (CETP) in Endothelial Cells Generates Vascular Oxidative Stress and Endothelial Dysfunction Biomolecules **11**, 10.3390/biom11010069
20. Agellon, L. B., Walsh, A., Hayek, T., Moulin, P., Jiang, X. C., Shelanski, S. A. *et al.* (1991) Reduced high density lipoprotein cholesterol in human cholesteryl ester transfer protein transgenic mice J Biol Chem **266**, 10796-10801,
21. Kumar, A., Patel, D. R., Brennan, D. M., Wolski, K. E., Lincoff, A. M., Ruotolo, G. *et al.* (2019) Plasma Aldosterone Levels Are Not Associated With Cardiovascular Events Among Patients With High-Risk Vascular Disease: Insights From the ACCELERATE Trial J Am Heart Assoc **8**, e013790 10.1161/JAHA.119.013790
22. Lincoff, A. M., Nicholls, S. J., Riesmeyer, J. S., Barter, P. J., Brewer, H. B., Fox, K. A. A. *et al.* (2017) Evacetrapib and Cardiovascular Outcomes in High-Risk Vascular Disease N Engl J Med **376**, 1933-1942 10.1056/NEJMoa1609581
23. Angelini, R., Yutuc, E., Wyatt, M. F., Newton, J., Yusuf, F. A., Griffiths, L. *et al.* (2021) Visualizing Cholesterol in the Brain by On-Tissue Derivatization and Quantitative Mass Spectrometry Imaging Anal Chem **93**, 4932-4943 10.1021/acs.analchem.0c05399
24. Nezhad, Z. S., Salazar, J. P., Pryce, R. S., Munter, L. M., andChaurand, P. (2022) Absolute quantification of cholesterol from thin tissue sections by silver-assisted laser desorption ionization mass spectrometry imaging Anal Bioanal Chem **414**, 6947-6954 10.1007/s00216-022-04262-6

28th May 2026

Dear Dr. Munter,

Thank you for the submission of your revised manuscript to EMBO Molecular Medicine. We have now received the enclosed report from the two referees who were asked to re-assess it. As you will see, the referees are now supportive, and I am pleased to inform you that we will be able to accept your manuscript pending the following amendments:

1. Remove "Authors' contribution" section from the manuscript file.

2. The PREVENT-AD Research Group collaborators should be moved to the Appendix and removed from the main manuscript.

3. Main figures should be provided as individual production quality Figure files and the legends should be placed in the manuscript file.

4. Remove" SENTENCE SUMMARY" from the manuscript file.

5. "COMPETING INTERESTS" should be renamed to "Disclosure and Competing Interests Statement".

6. The references need to be formatted according to the EMBO Molecular Medicine reference style. Please list up to 10 co-authors of a paper before adding et al. in the reference list. Citations should be listed in alphabetical order.

7. Data availability:
- the heading should be renamed to "Data availability".
- "All other data are available in the main text or the supplementary materials." should be removed.
- Please provide specific URL for GSE314221 dataset in this section.
- Please note that this statement applies only to the primary large-scale datasets generated in this study. If the PREVENT-AD datasets were not generated in this study, please remove the reference to them in this section.

8. Funding
- Funding information should be included under the Acknowledgments section. The "GENERAL" and "FUNDING" headings should be removed.
- The following funding information appears to be missing from the submission system and should be entered: Canada First Research Excellence Fund awarded to McGill University for Healthy Brains, Healthy Lives; Master's Fellowship and PhD Fellowship (JP); McGill University Goodman Cancer Institute Entrance Award; Fonds de recherche du Québec - Santé Master's Award (MSK, HN).

9. "Supplementary Material" should be renamed to "Appendix" throughout the file, since "Supplementary" and "Supplemental" should not be used; Add a title page with a table of contents that lists each item and its page number. The title should be "Appendix for [Manuscript title]". The correct nomenclature should be updated to Appendix Figure S1, etc. and Appendix Table S1, etc. in all places, including callouts; Merge the "Supplementary Methods" with the "Methods" section in the manuscript file.

10. The Source data need to be provided. At EMBO Press we ask authors to provide source data for the main manuscript figures. We have sent you a separate email with instructions for providing source data with your revised manuscript, on June 2nd 2025, including how to upload and organize the files.

11. Please provide a 'Synopsis' to further enhance discoverability. Synopses are displayed on the journal webpage and are freely accessible to all readers. They include a short stand first (maximum of 300 characters, including space) as well as 2-5 one-sentences bullet points that summarizes the paper. Please write the bullet points to summarize the key NEW findings. They should be designed to be complementary to the abstract - i.e. not repeat the same text. We encourage inclusion of key acronyms and quantitative information (maximum of 30 words / bullet point). Please use the passive voice.

Please provide the synopsis as a separate Word (DOCX) file

12. Please provide a visual abstract to illustrate your article as a PNG file exactly 550 px wide x 300 px high.

13. Please download and fill our Reagents and Tools Table template (.docx), which you can find in our author guidelines: https://www.embopress.org/page/journal/17574684/authorguide#structuredmethods

When submitting your revised manuscript, please do not include the Reagents and Tools Table in the Methods section of the manuscript but upload it as a separate file choosing the file type "Reagent Table".

14. Please provide "The paper explained"in the manuscript file : EMBO Molecular Medicine articles are accompanied by a summary of the articles to emphasize the major findings in the paper and their medical implications for the non-specialist reader. Please provide a draft summary of your article highlighting

- the medical issue you are addressing,
- the results obtained and
- their clinical impact.

This may be edited to ensure that readers understand the significance and context of the research. Please refer to any of our published articles for an example.

15. Please address the following issues in figure legends:
- Please note that the exact p values are not provided in the legends of figures 1D-H; 6D-H,K, L.
- Please indicate the statistical test used for data analysis in the legends of figures 5A, C, D; 6F, G, H, K, L; 6A-J.
- Please note that information related to n is missing in the legend of figure 1D
- Please note that the error bars are not defined in the legend of figure 5H.

16. Please correct the order and headings of the sections in the manuscript text file to: Abstract / The Paper Explained /Introduction / Results / Discussion / Methods / Data Availability / Acknowledgements / Disclosure and Competing Interests Statement / References / Figure Legends / Tables / Expanded View Figure Legends

I look forward to receiving a new revised version of your manuscript as soon as possible.

Kind regards,
Jingyi

Jingyi Hou
Senior Editor
EMBO Molecular Medicine

*** Instructions to submit your revised manuscript ***

*** PLEASE NOTE *** As part of the EMBO Publications transparent editorial process initiative (see our Editorial at https://www.embopress.org/doi/pdf/10.1002/emmm.201000094), EMBO Molecular Medicine will publish online a Review Process File to accompany accepted manuscripts.

In the event of acceptance, this file will be published in conjunction with your paper and will include the anonymous referee reports, your point-by-point response and all pertinent correspondence relating to the manuscript. If you do NOT want this file to be published, please inform the editorial office at contact@embomolmed.org.

***** Reviewer's comments *****

Referee #1 (Remarks for Author):

The authors have addressed all my questions

Referee #2 (Comments on Novelty/Model System for Author):

My recommendations have all been addressed in the revised manuscript.

Referee #2 (Remarks for Author):

The authors have addressed all my comments. I recommend the study for publication.

The authors addressed the remaining editorial issues.

25th Jun 2026

Dear Dr. Munter,

We are pleased to inform you that your manuscript is accepted for publication and is now being sent to our publisher to be included in the next available issue of EMBO Molecular Medicine.

Your manuscript will be processed for publication by EMBO Press. It will be copy edited and you will receive page proofs prior to publication. Please note that you will be contacted by Springer Nature Author Services to complete licensing and payment information.

You may qualify for financial assistance for your publication charges - either via a Springer Nature fully open access agreement or an EMBO initiative. Check your eligibility: https://link.springer.com/journal/44321/how-to-publish-with-us

Should you be planning a Press Release on your article, please notify the production team via this form in order to coordinate publication and release dates: https://forms.cloud.microsoft/e/eH4A8JVP6g

If you have any questions, please do not hesitate to contact the Editorial Office. Thank you for your contribution to EMBO Molecular Medicine.

Sincerely,
Jingyi

Jingyi Hou
Senior Editor
EMBO Molecular Medicine

>>> Please note that it is EMBO Molecular Medicine policy for the transcript of the editorial process (containing referee reports and your response letter) to be published as an online supplement to each paper. If you do NOT want this, you will need to inform the Editorial Office via email immediately. More information is available here: https://link.springer.com/partners/embo-press/editorial-policies#Peer%20review

EMBO Press Author Checklist

Corresponding Author Name: Lisa Marie Munter	
Journal Submitted to: EMBO Molecular Medicine	
Manuscript Number: EMM-2025-21688-V2	

USEFUL LINKS FOR COMPLETING THIS FORM
The EMBO Journal - Author Guidelines
EMBO Reports - Author Guidelines
Molecular Systems Biology - Author Guidelines
EMBO Molecular Medicine - Author Guidelines

Reporting Checklist for Life Science Articles (updated January
This checklist is adapted from Materials Design Analysis Reporting (MDAR) Checklist for Authors. MDAR establishes a minimum set of requirements in transparent reporting in the life sciences (see Statement of Task: 10.31222/osf.io/9sm4x). Please follow the journal's guidelines in preparing your
Please note that a copy of this checklist will be published alongside your article.

Abridged guidelines for figures
1. Data
The data shown in figures should satisfy the following conditions:
- the data were obtained and processed according to the field's best practice and are presented to reflect the results of the experiments in an accurate and unbiased manner.
- ideally, figure panels should include only measurements that are directly comparable to each other and obtained with the same assay.
- plots include clearly labeled error bars for independent experiments and sample sizes. Unless justified, error bars should not be shown for technical
- if n<5, the individual data points from each experiment should be plotted. Any statistical test employed should be justified.
- Source Data should be included to report the data underlying figures according to the guidelines set out in the authorship guidelines on Data

2. Captions
Each figure caption should contain the following information, for each panel where they are relevant:
- a specification of the experimental system investigated (eg cell line, species name).
- the assay(s) and method(s) used to carry out the reported observations and measurements.
- an explicit mention of the biological and chemical entity(ies) that are being measured.
- an explicit mention of the biological and chemical entity(ies) that are altered/varied/perturbed in a controlled manner.
- the exact sample size (n) for each experimental group/condition, given as a number, not a range;
- a description of the sample collection allowing the reader to understand whether the samples represent technical or biological replicates (including how many animals, litters, cultures, etc.).
- a statement of how many times the experiment shown was independently replicated in the laboratory.
- definitions of statistical methods and measures:
  - common tests, such as t-test (please specify whether paired vs. unpaired), simple χ2 tests, Wilcoxon and Mann-Whitney tests, can be unambiguously identified by name only, but more complex techniques should be described in the methods section;
  - are tests one-sided or two-sided?
  - are there adjustments for multiple comparisons?
  - exact statistical test results, e.g., P values = x but not P values < x;
  - definition of 'center values' as median or average;
  - definition of error bars as s.d. or s.e.m.

Please complete ALL of the questions below.
Select "Not Applicable" only when the requested information is not relevant for your study.

Materials

Newly Created Materials	Information included in the manuscript?	In which section is the information available? (Reagents and Tools Table, Materials and Methods, Figures, Data Availability Section)
New materials and reagents need to be available; do any restrictions apply?	Yes	McGill-APP-Thy1 mice need to be obtained through Dr. Claudio Cuello, referenced in the introduction and acknowledgement section.

Antibodies	Information included in the manuscript?	In which section is the information available? (Reagents and Tools Table, Materials and Methods, Figures, Data Availability Section)
For **antibodies** provide the following information:<br>- Commercial antibodies: RRID (if possible) or supplier name, catalogue number and or/clone number<br>- Non-commercial: RRID or citation	Yes	Supplier and catalog numbers are indicated for each antibody in the materials and methods section (as well as supplemental information)

DNA and RNA sequences	Information included in the manuscript?	In which section is the information available? (Reagents and Tools Table, Materials and Methods, Figures, Data Availability Section)
**Short novel DNA or RNA including primers, probes**: provide the sequences.	Yes	The supplemental information contains qPCR primers. In the "data availability" section is the GEO accession number for the RNA sequencing data.

Cell materials	Information included in the manuscript?	In which section is the information available? (Reagents and Tools Table, Materials and Methods, Figures, Data Availability Section)
**Cell lines:** Provide species information, strain. Provide accession number in repository **OR** supplier name, catalog number, clone number, and/**OR** RRID.	Yes	Cell line number is provided in Materials; we acknowledge the lab who donated them for this manuscript.
**Primary cultures:** Provide species, strain, sex of origin, genetic modification status.	Not Applicable	n/a
Report if the cell lines were recently **authenticated** (e.g., by STR profiling) and tested for mycoplasma contamination.	Yes	Yes, cell lines were frequently tested to be mycoplasm negative, and this is reported in the methods section.

Experimental animals	Information included in the manuscript?	In which section is the information available? (Reagents and Tools Table, Materials and Methods, Figures, Data Availability Section)
**Laboratory animals or Model organisms:** Provide species, strain, sex, age, genetic modification status. Provide accession number in repository **OR** supplier name, catalog number, clone number, **OR** RRID.	Yes	Species and genetic modifications are detailed in the supplemental materials. RRID for CETPtg mice is provided. Sex and age is reported in the results.
**Animal observed in or captured from the field:** Provide species, sex, and age where possible.	Not Applicable	
Please detail **housing and husbandry conditions**.	Yes	Detailed in the supplemental information

Plants and microbes	Information included in the manuscript?	In which section is the information available? (Reagents and Tools Table, Materials and Methods, Figures, Data Availability Section)
**Plants:** provide species and strain, ecotype and cultivar where relevant, unique accession number if available, and source (including location for collected wild specimens).	Not Applicable	
**Microbes:** provide species and strain, unique accession number if available, and source.	Not Applicable	

Human research participants	Information included in the manuscript?	In which section is the information available? (Reagents and Tools Table, Materials and Methods, Figures, Data Availability Section)
If collected and within the bounds of privacy constraints report on age, sex and gender or ethnicity for all study participants.	Yes	Materials section of the main manuscript

Core facilities	Information included in the manuscript?	In which section is the information available? (Reagents and Tools Table, Materials and Methods, Figures, Data Availability Section)
If your work benefited from core facilities, was their service mentioned in the acknowledgments section?	Yes	In acknowledgement section

Design

Study protocol	Information included in the manuscript?	In which section is the information available? (Reagents and Tools Table, Materials and Methods, Figures, Data Availability Section)
If study protocol has been **pre-registered, provide DOI in the manuscript**. For clinical trials, provide the trial registration number **OR** cite DOI.	Not Applicable	
Report the **clinical trial registration number** (at ClinicalTrials.gov or equivalent), where applicable.	Not Applicable	

Laboratory protocol	Information included in the manuscript?	In which section is the information available? (Reagents and Tools Table, Materials and Methods, Figures, Data Availability Section)
Provide DOI OR other citation details if **external detailed step-by-step protocols** are available.	Not Applicable	

Experimental study design and statistics	Information included in the manuscript?	In which section is the information available? (Reagents and Tools Table, Materials and Methods, Figures, Data Availability Section)
Include a statement about **sample size** estimate even if no statistical methods were used.	Yes	Supplemental information
Were any steps taken to minimize the effects of subjective bias when allocating animals/samples to treatment (e.g. **randomization procedure**)? If yes, have they been described?	Yes	Yes, in the methods in the main manuscript and in the supplemental info.
Include a statement about **blinding** even if no blinding was done.	Yes	Yes, added to the supplemental info.
Describe **inclusion/exclusion criteria** if samples or animals were excluded from the analysis. Were the criteria pre-established?<br>If sample or data points were omitted from analysis, report if this was due to attrition or intentional exclusion and provide justification.	Yes	Methods section in main mansucript and supplemental information
For every figure, are **statistical tests** justified as appropriate? Do the data meet the assumptions of the tests (e.g., normal distribution)? Describe any methods used to assess it. Is there an estimate of variation within each group of data? Is the variance similar between the groups that are being statistically compared?	Yes	The statistical test used are the ones commonly used for mouse studies with our sample size. The tests are reported in the Figure legends and post-hoc analyzed for multiple comparisons.

Sample definition and in-laboratory replication	Information included in the manuscript?	In which section is the information available? (Reagents and Tools Table, Materials and Methods, Figures, Data Availability Section)
In the figure legends: state number of times the experiment was **replicated** in laboratory.	Yes	Is reported in the Figure legends.
In the figure legends: define whether data describe **technical or biological replicates**.	Yes	technical and biological replicates are clearly identified in the Figure legends.

Ethics

Ethics	Information included in the manuscript?	In which section is the information available? (Reagents and Tools Table, Materials and Methods, Figures, Data Availability Section)
Studies involving **human participants**: State details of **authority granting ethics approval** (IRB or equivalent committee(s), provide reference number for approval.	Yes	REB protocol number for biomarkers was added to the methods of the main manuscript.
Studies involving **human participants**: Include a statement confirming that **informed consent** was obtained from all subjects and that the experiments conformed to the principles set out in the WMA Declaration of Helsinki and the Department of Health and Human Services Belmont Report.	Yes	Yes, added to the main manuscript text.
Studies involving **human participants: For** publication of **patient photos**, include a statement confirming that consent to publish was obtained.	Not Applicable	
Studies involving experimental **animals**: State details of **authority granting ethics approval** (IRB or equivalent committee(s), provide reference number for approval. Include a statement of compliance with ethical regulations.	Yes	Methods in the Supplemental information
Studies involving **specimen and field samples:** State if relevant **permits** obtained, provide details of authority approving study; if none were required, explain why.	Not Applicable	

Dual Use Research of Concern (DURC)	Information included in the manuscript?	In which section is the information available? (Reagents and Tools Table, Materials and Methods, Figures, Data Availability Section)
Could your study fall under dual use research restrictions? Please check biosecurity documents and list of **select agents and toxins** (CDC): https://www.selectagents.gov/sat/list.htm	Not Applicable	I don't think so
If you used a select agent, is the security level of the lab appropriate and reported in the manuscript?	Not Applicable	
If a study is subject to dual use research of concern regulations, is the name of the **authority granting approval and reference number** for the regulatory approval provided in the manuscript?	Not Applicable	

Reporting

The MDAR framework recommends adoption of discipline-specific guidelines, established and endorsed through community initiatives. Journals have their own policy about requiring specific guidelines and recommendations to complement MDAR.

Adherence to community standards	Information included in the manuscript?	In which section is the information available? (Reagents and Tools Table, Materials and Methods, Figures, Data Availability Section)
State if relevant guidelines or checklists (e.g., **ICMJE, MIBBI, ARRIVE, PRISMA**) have been followed or provided.	Yes	I added a statement in the supplemental information that ARRIVE guidelines were followed.
For **tumor marker prognostic studies,** we recommend that you follow the **REMARK** reporting guidelines (see link list at top right). See author guidelines, under 'Reporting Guidelines'. Please confirm you have followed these guidelines.	Not Applicable	
For **phase II and III randomized controlled trials**, please refer to the **CONSORT** flow diagram (see link list at top right) and submit the CONSORT checklist (see link list at top right) with your submission. See author guidelines, under 'Reporting Guidelines'. Please confirm you have submitted this list.	Not Applicable	

Data Availability

Data availability	Information included in the manuscript?	In which section is the information available? (Reagents and Tools Table, Materials and Methods, Figures, Data Availability Section)
Have **primary datasets** been deposited according to the journal's guidelines (see 'Data Deposition' section) and the respective accession numbers provided in the Data Availability Section?	Yes	RNAseq data available on GEO, accession number is provided in the "data availability" section.
Were **human clinical and genomic datasets** deposited in a public access-controlled repository in accordance to ethical obligations to the patients and to the applicable consent agreement?	Yes	Statement for the PREVENT-AD cohort is included on how to obtain access.
Are **computational models** that are central and integral to a study available without restrictions in a machine-readable form? Were the relevant accession numbers or links provided?	Not Applicable	
If publicly available data were reused, provide the respective **data citations in the reference list**.	Yes	Citations for PREVENT AD were included.
