# Supplementary material for: Reducing CETP activity prevents memory decline in an Alzheimer’s disease mouse model
Source: EMBO Mol Med. 2026 Aug 3;18(9):3597–618. doi: 10.1038/s44321-026-00482-w (PMC13562521; doi:10.1038/s44321-026-00482-w)
Supplement: Supplementary file 3 [file 44321_2026_482_MOESM3_ESM.zip › Figure 1/1 D-H/Protocol for CETP activity assay from Sigma by Jasmine Phenix.docx]

**Protocol for CETP activity assay from Sigma by Jasmine Phenix**

Materials:

- Ice bucket
- 96-well U-bottom black plates for fluorescence assays (we use demi wells)
- 37C water bath incubator
- Isopropanol (also called 2-propanol)
- 20mM Evacetrapib in DMSO (I suggest a higher concentration or mix it well and use it fresh)

1. Get ice and aluminum foil.
2. Put the bottles (buffer, acceptor particle tube, donor particle tube) on ice.
3. Get your serum samples from the -80C and let them thaw on ice.
4. Get your Evacetrapib aliquot and let thaw at room temperature, covered with foil.
5. Start by diluting your Evacetrapib in DMSO by 1:100 dilution factor, then repeat the previous dilution to reach a final concentration of 1:10,000 (2uM final concentration), cover with foil.
6. Dilute your samples by a factor of 1:15 in buffer in fresh tubes and leave on ice

This dilution was found by me experimentally (I tested 1/5, 1/15 and 1/25 and 1/50), but it really depends on what samples you work with, but it is a good general rule for diluting samples without oversaturating the assay.

7. Prepare your master-master mix (containing buffer, acceptor and donor only) and leave the vial on ice covered by foil.
   a. Master-master mix will have:
      i. Buffer: 29ul x number of samples x 2 + extras
      ii. Acceptor: 0.6ul x number of samples x 2 +extras
      iii. Donor: 0.6ul x number of samples x 2 +extras
      iv. Sample dilution: 1ul x number of samples x 2 +extras
      v. Inhibitor: 0.3ul x number of samples x 2 +extras *optional*
8. Prepare your standards for your curve, vortex and spin between each tube, and leave on ice covered with foil
9. Prepare your individual submaster mixes for each sample (master-master mix, sample dilution, and in some case the Evacetrapib dilution. Mix by pipetting up and down.

DO NOT VORTEX SAMPLES, the protein CETP in it might be affected by vortexing and not be able to perform its function.

10. Pipette 31.2ul of the standard curve, the submaster mixes in duplicates, and the mixes with Evacetrapib into each well.

The reason for adding the master mix and THEN the sample (with additional drug or not) is because we suspect that the lipids are sticking to the pipette tips and reducing the intensity of the signal.

11. Cover the wells with a clear plastic film and roll over with the qPCR roller.
12. Cover in foil and put in the water bath for 3h.

For this step, make sure that the water is not going into your boat and that light is not shinning onto your samples. I also noticed that the outer wells with the standard curves are always very off in terms of values (too low). Perhaps leave 2 rows (start curve in row 3 instead of 2) to avoid this edge effect.

13. Remove the foil and spin the plate at 3000rpm for 30 seconds. Don’t forget to balance.
14. Remove the plastic film delicately and place the 96-well into the plate reader.
15. Read the plate by selecting the ‘Lotus modified’ protocol from Felix modified by Lisa and I (done for ½ wells)
16. Save file by exporting and clicking the Excel button.
