# Supplementary material for: Reducing CETP activity prevents memory decline in an Alzheimer’s disease mouse model
Source: EMBO Mol Med. 2026 Aug 3;18(9):3597–618. doi: 10.1038/s44321-026-00482-w (PMC13562521; doi:10.1038/s44321-026-00482-w)
Supplement: Supplementary file 4 [file 44321_2026_482_MOESM4_ESM.zip › Figure 2/Raw data from CMAR.pdf]

Comparative Medicine Animal Resources Centre

McGill

Diagnostic Laboratory
tel: (514) 398-8289
e-mail: drss@mcgill.ca

Date received: 21.02.10

Investigator: Dr. Lisa Munter | Department: Pharmacology | Bldg/Room: GCC
Sample date: 21.02.10 | Specimen: Mouse serum | Requested by: Jasmine Phenix

Animal ID	Units	**Values**	221	223	311	611	612	613	615	822
**Laboratory number**		**Mouse**	**21-0175**	**21-0176**	**21-0177**	**21-0178**	**21-0179**	**21-0180**	**21-0181**	**21-0182**
Glucose	mmol/L	**5.0-10.7**	19.0	17.9	19.4	21.8	25.9	23.7	19.7	17.4
Cholesterol	mmol/L	**0.93-2.48**	3.34	3.40	3.94	3.37	2.95	2.98	3.05	3.35
HDL	mmol/L		3.20	2.92	3.55	2.74	2.65	2.54	2.69	2.63
Triglycerides	mmol/L	**0.62-1.63**	1.17	1.22	1.11	1.17	1.08	0.93	0.65	1.04
LDL	mmol/L		*	*	*	0.09	*	0.02	0.06	0.24

Animal ID	Units	**Values**	823	1112	1113**	261	751	754	234	641
**Laboratory number**		**Mouse**	**21-0183**	**21-0184**	**21-0185**	**21-0186**	**21-0187**	**21-0188**	**21-0189**	**21-0190**
Glucose	mmol/L	**5.0-10.7**	28.2	24.0	20.1	17.6	15.1	15.3	12.6	12.8
Cholesterol	mmol/L	**0.93-2.48**	2.35	2.19	1.72	2.68	2.61	2.60	2.42	2.42
HDL	mmol/L		2.01	1.60	0.68	2.10	2.53	2.06	1.79	1.54
Triglycerides	mmol/L	**0.62-1.63**	1.26	1.82	1.37	0.92	0.90	0.93	0.38	0.62
LDL	mmol/L		*	*	0.41	0.15	*	0.11	0.45	0.61

Animal ID	Units	**Values**	415	652	745**	924	925	1033	1034	1041**
**Laboratory number**		**Mouse**	**21-0191**	**21-0192**	**21-0193**	**21-0194**	**21-0195**	**21-0196**	**21-0197**	**21-0198**
Glucose	mmol/L	**5.0-10.7**	13.6	21.0	17.4	22.7	29.4	13.7	15.8	21.8
Cholesterol	mmol/L	**0.93-2.48**	3.26	2.37	1.92	2.03	1.92	2.71	2.40	2.30
HDL	mmol/L		2.81	1.89	1.36	1.69	1.15	1.22	1.67	1.17
Triglycerides	mmol/L	**0.62-1.63**	0.46	0.76	1.10	0.79	0.88	0.55	0.40	1.37
LDL	mmol/L		0.24	0.14	0.06	*	0.37	1.24	0.55	0.50

Animal ID	Units	**Values**	1042	1143	1131	1133**	213	214**	614	622**
**Laboratory number**		**Mouse**	**21-0199**	**21-0200**	**21-0201**	**21-0202**	**21-0203**	**21-0204**	**21-0205**	**21-0206**
Glucose	mmol/L	**5.0-10.7**	20.9	20.3	14.0	28.3	20.2	13.5	18.6	27.2
Cholesterol	mmol/L	**0.93-2.48**	2.42	1.91	2.14	2.47	3.46	2.72	3.80	3.07
HDL	mmol/L		1.25	1.35	1.36	1.09	2.93	2.31	2.92	2.62
Triglycerides	mmol/L	**0.62-1.63**	1.13	0.74	0.63	1.21	0.93	1.16	0.84	1.32
LDL	mmol/L		0.65	0.22	0.50	0.83	0.10	*	0.50	*

* Unable to calculate.
** Slightly Hemolyzed

Reported by: [illegible]

Date: 21.03.08

Comparative Medicine Animal Resources Centre

McGill

Diagnostic Laboratory
tel: (514) 398-8289
e-mail: drss@mcgill.ca

Date received: 21.02.10

Investigator: Dr. Lisa Munter | Department: Pharmacology | Bldg/Room: GCC
Sample date: 21.02.10 | Specimen: Mouse serum | Requested by: Jasmine Phenix

Animal ID	Units	**Values**	722	724	732	821	911	913**	1013	662
**Laboratory number**		**Mouse**	**21-0207**	**21-0208**	**21-0209**	**21-0210**	**21-0211**	**21-0212**	**21-0213**	**21-0214**
Glucose	mmol/L	**5.0-10.7**	29.4	31.5	20.9	19.3	25.9	17.1	26.0	20.7
Cholesterol	mmol/L	**0.93-2.48**	2.85	3.14	2.99	2.76	3.72	2.83	2.66	3.58
HDL	mmol/L		2.42	2.31	2.74	2.25	2.84	1.81	2.22	3.16
Triglycerides	mmol/L	**0.62-1.63**	1.05	0.97	1.07	1.18	1.27	1.35	1.02	1.06
LDL	mmol/L		*	0.38	*	*	0.30	0.41	*	*

Animal ID	Units	**Values**	663	664	542	674	774	944	642	651**
**Laboratory number**		**Mouse**	**21-0215**	**21-0216**	**21-0217**	**21-0218**	**21-0219**	**21-0220**	**21-0221**	**21-0222**
Glucose	mmol/L	**5.0-10.7**	19.3	14.3	15.1	10.4	18.2	13.0	17.8	19.5
Cholesterol	mmol/L	**0.93-2.48**	2.97	2.61	1.87	2.75	2.67	2.37	1.99	2.00
HDL	mmol/L		2.41	2.04	1.46	1.77	2.22	2.08	1.46	1.38
Triglycerides	mmol/L	**0.62-1.63**	1.10	0.95	0.44	0.34	1.26	0.45	0.83	1.08
LDL	mmol/L		0.06	0.13	0.20	0.83	*	0.08	0.15	0.12

Animal ID	Units	**Values**	744	814**	1051	1024	1031	1134	832	833
**Laboratory number**		**Mouse**	**21-0223**	**21-0224**	**21-0225**	**21-0226**	**21-0227**	**21-0228**	**21-0229**	**21-0230**
Glucose	mmol/L	**5.0-10.7**	24.6	24.3	16.2	9.0	28.7	15.4	14.7	15.6
Cholesterol	mmol/L	**0.93-2.48**	1.64	2.65	2.07	2.33	2.06	2.13	2.05	2.83
HDL	mmol/L		1.35	2.37	1.45	1.44	1.28	1.27	1.24	1.82
Triglycerides	mmol/L	**0.62-1.63**	0.67	1.18	0.98	0.85	1.47	0.48	0.68	0.60
LDL	mmol/L		*	*	0.17	0.50	0.10	0.64	0.50	0.73

Animal ID	Units	**Values**	222	621	624	631	723	733	1121	812
**Laboratory number**		**Mouse**	**21-0231**	**21-0232**	**21-0233**	**21-0234**	**21-0235**	**21-0236**	**21-0237**	**21-0238**
Glucose	mmol/L	**5.0-10.7**	25.6	13.7	11.0	19.2	22.8	9.2	23.7	17.9
Cholesterol	mmol/L	**0.93-2.48**	3.70	3.10	3.20	3.47	4.08	3.57	3.53	2.63
HDL	mmol/L		2.78	2.71	2.75	3.18	2.26	1.20	3.10	1.70
Triglycerides	mmol/L	**0.62-1.63**	1.16	1.46	1.60	1.08	1.26	1.06	1.18	1.48
LDL	mmol/L		0.39	*	*	*	1.25	1.88	*	0.25

* Unable to calculate.
** Slightly Hemolyzed

Reported by: [signature]

Date: 21.03.08

Comparative Medicine Animal Resources Centre

McGill

Diagnostic Laboratory
tel: (514) 398-8289
e-mail: drss@mcgill.ca

Date received: 21.02.10

Investigator: Dr. Lisa Munter | Department: Pharmacology | Bldg/Room: GCC
Sample date: 21.02.10 | Specimen: Mouse serum | Requested by: Jasmine Phenix

Animal ID	Units	Values	813	824	111	914	1011	1111	235	241
**Laboratory number**		**Mouse**	**21-0239**	**21-0240**	**21-0241**	**21-0242**	**21-0243**	**21-0244**	**21-0245**	**21-0246**
Glucose	mmol/L	**5.0-10.7**	21.1	25.3	17.4	23.9	25.4	22.4	14.1	10.5
Cholesterol	mmol/L	**0.93-2.48**	2.51	2.70	3.07	3.53	3.08	2.53	2.39	2.39
HDL	mmol/L		1.75	1.42	1.90	2.36	1.88	1.05	2.19	1.97
Triglycerides	mmol/L	**0.62-1.63**	1.14	1.70	1.23	1.17	1.13	2.38	0.43	0.56
LDL	mmol/L		0.24	0.50	0.61	0.64	0.69	0.39	0.01	0.16

Animal ID	Units	Values	242	315**	742**	921	761**	1032	1035**	1135
**Laboratory number**		**Mouse**	**21-0247**	**21-0248**	**21-0249**	**21-0250**	**21-0251**	**21-0252**	**21-0253**	**21-0254**
Glucose	mmol/L	**5.0-10.7**	10.6	19.3	15.9	21.3	17.4	26.1	21.2	32.1
Cholesterol	mmol/L	**0.93-2.48**	2.32	2.27	2.82	2.08	3.79	2.34	1.94	1.98
HDL	mmol/L		1.91	1.50	1.95	1.89	2.58	0.98	0.65	0.72
Triglycerides	mmol/L	**0.62-1.63**	0.48	0.98	0.93	0.76	1.79	0.92	1.35	0.91
LDL	mmol/L		0.19	0.32	0.44	*	0.39	0.93	0.67	0.84

Animal ID	Units	Values	831	762	932	523	211	212	312	623
**Laboratory number**		**Mouse**	**21-0255**	**21-0256**	**21-0257**	**21-0258**	**21-0259**	**21-0260**	**21-0261**	**21-0262**
Glucose	mmol/L	**5.0-10.7**	26.9	14.8	12.9	14.2	21.6	26.2	22.6	18.3
Cholesterol	mmol/L	**0.93-2.48**	2.49	2.33	2.28	2.70	3.81	3.33	4.35	3.98
HDL	mmol/L		1.21	0.97	0.96	1.01	3.23	2.71	3.84	2.85
Triglycerides	mmol/L	**0.62-1.63**	1.39	0.52	0.77	0.66	1.01	1.16	1.13	1.20
LDL	mmol/L		0.64	1.12	0.97	1.39	0.12	0.09	*	0.58

Animal ID	Units	Values	632	633	731	811	1012	1122	1123	661
**Laboratory number**		**Mouse**	**21-0263**	**21-0264**	**21-0265**	**21-0266**	**21-0267**	**21-0268**	**21-0269**	**21-0270**
Glucose	mmol/L	**5.0-10.7**	20.7	22.1	20.1	20.8	27.6	22.4	23.5	21.5
Cholesterol	mmol/L	**0.93-2.48**	3.71	2.81	2.79	3.32	2.83	4.47	3.87	2.33
HDL	mmol/L		3.19	2.51	2.02	2.62	1.44	3.84	3.08	1.39
Triglycerides	mmol/L	**0.62-1.63**	1.37	1.42	0.98	1.32	1.20	1.72	1.35	0.85
LDL	mmol/L		*	*	0.32	0.09	0.84	*	0.17	0.55

* Unable to calculate.
** Slightly Hemolyzed

Reported by: [signature]

Date: 21.03.08

Comparative Medicine Animal Resources Centre

McGill

Diagnostic Laboratory
tel: (514) 398-8289
e-mail: drss@mcgill.ca

Date received: 21.02.10

Investigator: Dr. Lisa Munter
Department: Pharmacology
Bldg/Room: GCC
Sample date: 21.02.10
Specimen: Mouse serum
Requested by: Jasmine Phenix

Animal ID	Units	**Values**	263**	755	543	643	644	741	743**	922
**Laboratory number**		**Mouse**	**21-0271**	**21-0272**	**21-0273**	**21-0274**	**21-0275**	**21-0276**	**21-0277**	**21-0278**
Glucose	mmol/L	**5.0-10.7**	20.4	16.6	14.8	11.1	9.8	10.1	18.0	15.9
Cholesterol	mmol/L	**0.93-2.48**	3.49	3.96	3.39	2.44	2.48	2.94	4.85	2.41
HDL	mmol/L		2.21	2.64	2.49	1.51	1.94	2.50	1.75	1.81
Triglycerides	mmol/L	**0.62-1.63**	2.04	1.37	1.32	0.54	0.41	0.47	2.71	0.80
LDL	mmol/L		0.35	0.69	0.30	0.68	0.36	0.22	1.85	0.24

Animal ID	Units	**Values**	923	1021	1144**	931	933	934	521	522
**Laboratory number**		**Mouse**	**21-0279**	**21-0280**	**21-0281**	**21-0282**	**21-0283**	**21-0284**	**21-0285**	**21-0286**
Glucose	mmol/L	**5.0-10.7**	18.3	21.4	30.8	13.6	11.5	11.6	20.2	22.4
Cholesterol	mmol/L	**0.93-2.48**	3.44	2.17	2.24	2.67	2.50	2.30	4.66	2.88
HDL	mmol/L		2.68	1.47	1.01	0.94	0.92	0.96	2.07	0.95
Triglycerides	mmol/L	**0.62-1.63**	1.10	1.39	1.71	1.21	0.72	0.65	1.34	1.17
LDL	mmol/L		0.25	0.06	0.45	1.18	1.24	1.04	1.97	1.39

* Unable to calculate.
** Slightly Hemolyzed

Reported by:

Date: 21.03.08
